# Xenobiotic-metabolizing enzymes in the skin of rat, mouse, pig, guinea pig, man, and in human skin models

**DOI:** 10.1007/s00204-014-1382-8

**Published:** 2014-11-05

**Authors:** F. Oesch, E. Fabian, K. Guth, R. Landsiedel

**Affiliations:** 1 Oesch-Tox Toxicological Consulting and Expert Opinions GmbH&Co.KG, Rheinblick 21, 55263 Wackernheim, Germany; 2 Experimental Toxicology and Ecology, GV/TB, Z470, BASF SE, Carl-Bosch-Str. 38, 67056 Ludwigshafen, Germany

**Keywords:** Cutaneous xenobiotic metabolism, Species differences, Human skin models, Rat, Mouse, Pig, Guinea pig

## Abstract

The exposure of the skin to medical drugs, skin care products, cosmetics, and other chemicals renders information on xenobiotic-metabolizing enzymes (XME) in the skin highly interesting. Since the use of freshly excised human skin for experimental investigations meets with ethical and practical limitations, information on XME in models comes in the focus including non-human mammalian species and in vitro skin models. This review attempts to summarize the information available in the open scientific literature on XME in the skin of human, rat, mouse, guinea pig, and pig as well as human primary skin cells, human cell lines, and reconstructed human skin models. The most salient outcome is that much more research on cutaneous XME is needed for solid metabolism-dependent efficacy and safety predictions, and the cutaneous metabolism comparisons have to be viewed with caution. Keeping this fully in mind at least with respect to some cutaneous XME, some models may tentatively be considered to approximate reasonable closeness to human skin. For dermal absorption and for skin irritation among many contributing XME, esterase activity is of special importance, which in pig skin, some human cell lines, and reconstructed skin models appears reasonably close to human skin. With respect to genotoxicity and sensitization, activating XME are not yet judgeable, but reactive metabolite-reducing XME in primary human keratinocytes and several reconstructed human skin models appear reasonably close to human skin. For a more detailed delineation and discussion of the severe limitations see the “Overview and Conclusions” section in the end of this review.

## Introduction

Extrahepatic xenobiotic metabolism in organs of the body’s internal–external interfaces such as intestine, lung, and skin comes more and more into the center of interest in the field of xenobiotic metabolism studies (Gundert-Remy et al. 2014). In 2007, we published a review on xenobiotic-metabolizing enzymes (XME) in the skin of man, rat, and pig (Oesch et al. 2007). The purpose of the present review was to update the data on of the skin of man, rat, and pig in this rapidly developing field. The review addresses in addition XME in the skin of the two routinely used species for testing compounds for skin sensitization, the mouse and the guinea pig. For the human situation, we also included the XME in skin-derived cells and cell lines and human reconstructed skin models. In order not to force the reader to go back to our 2007 review (Oesch et al. 2007), we also include in short and succinctly the data on XME in the skin of man, rat, and pig published prior to 2007. However, the general considerations on the layers of the mammalian skin as well as the use of skin cell cultures presented in our original review (Oesch et al. 2007) are not repeated here, and the interested reader is referred to our original review (Oesch et al. 2007).

The main purpose of this review was to present information which—with all due caution because of limited comparability of data—may aid in selecting models—be it non-human mammalian species, be it human in vitro skin models—with hopefully sufficient metabolic closeness to human skin for adequately supporting the predictions of metabolism-dependent toxicities or desired pharmacological activities of xenobiotic compounds applied to the human skin. Obviously, a model which corresponds to the human skin with respect to all XME may never be found. Since the metabolic impact on different end points will not necessarily depend on the same XME, different models may turn out to be preferable for different end points. In the last chapter of this review “Summary and Conclusions,” an attempt to approximate an estimation of relative suitability of the individual patterns of various XME in models for human skin with respect to dermal absorption, genotoxicity/chemical carcinogenicity, sensitization, and skin irritation is presented.

As an interesting side line a general remark before going to details: despite extensive metabolism of many xenobiotics in rat skin as will be detailed below, some, such as coumarin (Beckley-Kartey et al. 1997; Yourick and Bronaugh 1997), caffeine, and DDT (Bronaugh et al. 1989), were reported not to be metabolized in rat skin. Also in pig skin, some xenobiotics appear not or essentially not to be metabolized including drugs very well metabolized in the pig liver, such as rivastigmine that leads by topical application to the minipig skin—bypassing the extensive first-pass metabolism in the liver—to a high systemic availability (Tse and Laplanche 1998). Some compounds which come into contact with the human skin in important consumer products and in topical therapeutic agents such as coumarin have been reported to be extensively absorbed through the (unoccluded or occluded) human skin without being metabolized (Beckley-Kartey et al. 1997; Yourick and Bronaugh 1997).

An important word of caution is in order. Many cutaneous xenobiotic-metabolizing enzyme activities, e.g., those of cytochrome P450 (CYP), are very low and therefore not easy to be measured accurately. The XME activities of species and models reported in this review stem from different laboratories. Unfortunately, in most cases, it is not indicated in the original publications whether linearity of the given activities with respect to time and with respect to the amount of protein/amount of tissue used has been ascertained or whether the obtained results just have been divided by the minutes (hours) and milligrams (grams) used in the experiments. Hence, a transformation to comparable standard units would be misleading, and the numerically given activities in many cases may not be strictly comparable. The units given in this review are therefore those given in the original publications. Moreover, in most cases, the limit of detection (LOD) and/or limit of quantification (LOQ) are not given in the original publications (and probably were not determined) such that very low activities versus zero activity or “not detected” may have different meanings between different original publications. Finally, quite obviously, the different assay methods used in different laboratories in addition make comparisons difficult. The main purpose of the numerical values given in this review was to show that numerically measurable activities were reported and may, with due caution, be considered as high, moderate, or low.

## Xenobiotic-metabolizing enzymes in the rat skin

### Cytochromes P450 (CYP)

#### CYP transcript expression

mRNA coding for CYP1A1, CYP1A2, CYP2B1/2, CYP2C11, CYP2E1, CYP3A1, and CYP4A1 was expressed in the skin of Sprague–Dawley rats between the ages of 6 and 8 weeks (Lee et al. 2001). As expected, expression of CYP1A2, CYP2B1/2, CYP2E1, CYP3A1, and CYP4A1 was lower in the skin than in the liver. However, expression of CYP1A1 and CYP2C11 was higher in the skin than in the liver.

#### CYP protein expression

CYP1A1 has been studied extensively in the rat skin because of its role in procarcinogen metabolism (Einolf et al. 1997; Khan et al. 1992; Raza et al. 1992). CYP reductase (NADPH cytochrome P-450 oxidoreductase), which is required for CYP activity, has been shown by immunohistochemical staining to be predominantly localized in the epidermis (Takahara et al. 1993).

In early studies, CYP b (now termed CYP2B1) protein was observed by radioimmunoassay of epidermal microsomes from untreated rats. CYP c (now termed CYP1A1) was observed in epidermal microsomes prepared from 3-methylcholanthrene (3-MC, an often-used experimental inducer of the CYP1 family)-treated rats. Histochemical staining of epidermis from untreated rats showed CYP2B1 protein and staining of epidermis from 3-MC-treated rats CYP1A1, respectively (Khan et al. 1989). Pendlington et al. (1994) observed constitutive CYP1A1/A2 and CYP2B1/B2 concentrated in the epidermis and sebaceous glands of the rat.

In more recent studies, the following CYP proteins were observed in the skin microsomal fraction from untreated rats by immunoblotting using a panel of mono-specific antibodies: CYP2B, CYP2C13, CYP2D1, CYP2D4, CYP2E1, CYP3A1, and CYP3A2 (Zhu et al. 2002).

CYP2D4 protein was observed in the rat skin but not in the rat liver while the other CYPs observed in the rat skin were also expressed in the rat liver. With the exception of CYP2D4, the levels of these CYPs were much lower in skin compared with liver: 2.3 % for CYP2B, 0.3 % for CYP2E1, 1.3 % for CYP2C13, 0.1 % for CYP2D1, 4.7 % for CYP3A1, and 0.4 % for CYP3A2. No CYP1A1, CYP1A2, or CYP2C12 protein was found, although these were clearly detected in rat liver samples (Zhu et al. 2002).

A novel CYP, termed CYP2B12, was found not to be expressed in the liver but in the preputial gland (Friedberg et al. 1990, 1992) and then discovered to be a keratinocyte-specific 11,12- and 8,9-epoxygenase of arachidonic acid having a high, constitutive expression in the sebaceous glands, being expressed exclusively in a subset of differentiated keratinocytes called sebocytes. Its expression coincides with the morphological appearance of sebaceous glands in the neonatal rat (Keeney et al. 1998a, b).

In rat keratinocytes serum-free monolayer cultures on day 0, CYP3A1 and CYP3A2 proteins were poorly expressed and CYP2E1 was undetectable. The levels of all three of these CYPs increased to maximum expression when the cells approached confluence between days 10–14. At this time point, levels became similar to those observed in the native skin: 137, 98, and 104 % CYP2E1, CYP3A1, and CYP3A2 compared with the respective levels in the rat skin microsomal fractions (Zhu et al. 2002). Thus, the usefulness of freshly isolated epidermal cells is questionable for studies on CYP-mediated metabolism and its consequences. However, 10- to 14-day cultured keratinocytes express CYPs at levels close to those of whole skin and therefore may provide a good model for investigating the metabolism and toxicity of xenobiotics in the skin.

#### CYP catalytic activities (see also Table 1)

The enzymatic activity metabolizing polycyclic aromatic hydrocarbon carcinogens most frequently is determined by measuring the formation of hydroxylated metabolites from the aromatic hydrocarbon (“aryl hydrocarbon”) benzo[a]pyrene (BP), and this activity is frequently termed “aryl hydrocarbon hydroxylase (AHH). AHH activity was observed in skin microsomes of neonatal rats. Skin had 2, 21, and 27 % of whole-body AHH activity in control, benzo[a]pyrene (BP)-treated, and Aroclor 1254-treated animals, respectively. Aminopyrine, a substrate for several CYPs, was also metabolized by microsomes from the skin of neonatal rats (Mukhtar and Bickers 1981). 7-Ethoxyresorufin (EROD) (selective substrate for CYP1 family), 7-pentoxyresorufin (PROD) (selective substrate for CYP2B family), and 7-benzyloxyresorufin (BROD) (substrate for a broader spectrum of rat CYPs including 1A1, 2B1/2, and 3A1) were oxidatively dealkylated in rat skin microsomes at a rate 8–16 times less than in rat liver microsomes while hydroxylation of lauric acid (selective substrate for rat CYP4A1) was not detected in rat skin microsomes (Pham et al. 1989).

**Table 1:** Representative cytochrome P450 (CYP) basal activities in skin microsomes of various mammalian species

Activity (preferential for)	Human	Rat	Mouse	Guinea pig	Pig
AHH (CYP1 family)	0.24–1.35^a^	1.25 ± 0.11^a^	m^b^: 3.3–8^a^; f^b^: 17–21^a^	2.51 ± 0.35^a^	
EROD (CYP1 family)	bd–35^a^	m: 3.6 ± 0.3; f: 1.5 ± 0.2^a^	m: bd; f: 3–19^a^		4.62 ± 0.54^b^
ECOD (CYP1A, 2B)	bd–12^a^	0.36–2.15^a^	10.4–80^a^	3.8 ± 2.7^a^	(13.2 ± 2.5^b^)
MROD (CYP1A2)	bd to +				
PROD (CYP2B)	bd to bq	m: 3.7 ± 1.3; f: 1.8 ± 0.1^a^	m: bq; f: 0.1–1.7^a^	bq	bd
BROD (CYP3A, 2B)		m: 4.4 ± 0.9; f: 2.1 ± 0.2^a^			
Aminopyrine-*N*-demethylase (CYP2B, 3A)		1,000–4200^a^	+		
Tolbutamide 4-hydroxylation (CYP2C9)	0.46 ± 0.05^b^	0.47 ± 0.04^b^	bd		1.66 ± 0.49^b^
Bufuralol 1-hydroxylation (CYP2D6)	bd	1.33 ± 0.17^b^	9.23 ± 0.67^b^		0.26 ± 0.03^b^
Chlorzoxazone 6-hydroxylation (CYP2E1)	2.83 ± 0.34^b^	bd	20.8 ± 0.5^b^		bd
Para-nitrophenol hydroxylation (CYP2E1)	bd/+	bd^c^	f: 40 ± 10^a^		
Midazolam 1-hydroxylation (CYP3A)	2.35 ± 0.23^b^	0.58 ± 0.09^b^	8.70 ± 0.28^b^		2.32 ± 0.21^b^
Benzoquinoline *O*-dealkylation (CYP3A)	bd–76 ± 41^a^				
Erythromycin *N*-demethylation (CYP3A)	+	bd–270^a^	f: 540–1100^a^		

More examples and references in the text

*AHH* aryl hydrocarbon hydroxylase, phenolic benzo[*a*]pyrene metabolites determined with 3-hydroxy-benzo[*a*]pyrene as standard, *bd* below detection, *BROD* 7-benzyloxyresorufin *O*-debenzylase, *bq* below quantification, *ECOD* 7-ethoxycoumarin *O*-deethylase, *EROD* 7-ethoxyresorufin *O*-deethylase, *f* female, *m* male, *MROD* 7-methoxyresorufin *O*-demethylase, *PROD* pentoxyresorufin *O*-depentylase

^a^pmol/mg protein/min

^b^pmol/mg protein/h, numbers in brackets: in medium of short-term culture

^c^In epidermal microsomes

In a comparative study, Rolsted et al. (2008) found in rat skin microsomes the following activities (expressed in pmol metabolites/h/mg protein): EROD 1.01 ± 0.14, PROD”not detected” (LOQ [limit of quantification]1.87 pmol), tolbutamide 4-hydroxylation (prototypical for CYP2C9) 0.47 ± 0.04, bufuralol 1-hydroxylation (prototypical for CYP 2D6) 1.33 ± 0.17, chlorzoxazone 6-hydroxylation (prototypical for CYP2E1)”not detected” (LOQ 12.8 pmol), midazolam 1-hydroxylation (prototypical for CYP3A) 0.58 ± 0.09.

##### Localization

In decreasing order of potency, the NADPH (cofactor for CYP activity) fortified 9000 g supernatant (S9) fraction of rat epidermis, dermis, and whole skin converted 2-aminoanthracene to metabolites that were mutagenic to the *Salmonella typhimurium* tester strains TA1537, TA1538, TA98, and TA100 (Bickers et al. 1985). Incubation of neonatal rat skin microsomes prepared from whole skin, dermis, or epidermis with testosterone resulted in the formation of 6-beta-, 7-alpha-, and 16-alpha-hydroxytestosterone. Maximum hydroxylation occurred in epidermal microsomes followed by whole skin and dermis microsomes. The reaction required NADPH. Addition of SKF-525A and metyrapone (1 mM) (CYP inhibitors) resulted in (75 and 70 %) inhibition (Mukhtar et al. 1987), all consistent with CYP being responsible for the testosterone hydroxylation reactions in rat skin microsomes. In the rat, the formation of 6-beta-hydroxytestosterone is preferentially catalyzed by CYP3A1/2 (and CYP 1A1), that of 7-alpha-hydroxytestosterone by CYP2A1/2, and that of 16-alpha-hydroxytestosterone by CYP2C11 and CYP 2B1/2.

*Induction.* Wattenberg and Leong (1962) already showed that topical application of 3-MC to the rat skin led to an increase in the cutaneous AHH activity. Mukhtar and Bickers (1981) reported that the AHH activity was increased after topical application of BP or the polychlorinated biphenyl mixture Aroclor 1254 and that also EROD activity was increased. After application of 3-MC (Khan et al. 1989) or nitroarenes (3-nitrofluoranthene, 1-nitropyrene) (Asokan et al. 1986) to the rat skin, AHH, 7-ethoxycoumarin *O*-deethylase (ECOD; preferentially catalyzed by CYP1A/2B), and EROD activities were induced in epidermal microsomes. Topical application of the dermatological antifungal agent clotrimazole to the rat skin led to an increase in para-nitrophenol hydroxylase, a diagnostic activity for the ethanol-inducible CYP2E1, which was not observed in the untreated rat skin (Merk et al. 1989). After 7-, 14-, and 28-day UVB irradiation of adult female Wistar rats, cutaneous erythromycin demethylase (but not EROD nor aminopyrine-*N*-demethylase) activity was increased (Goerz et al. 1996).

In epidermal cells obtained from the skin of newborn rats exposed to benz[a]anthracene by topical exposure and in submerged cultures exposed to benz[a]anthracene, in vitro induction of microsomal AHH and CYP was observed. The level of AHH activity was increased 2.5-fold in vivo and 6- to 7-fold in vitro when the measurements were taken on the entire epidermis or the entire culture, respectively. Separate measurement on germinative (basal) and on differentiated cells showed that AHH was sevenfold higher in differentiated cells compared with basal cells in the skin of both unexposed animals and animals exposed in vivo. Similar results were obtained in cultured cells exposed in vitro.

Immunocytochemical staining of sections of skin from animals exposed to benz[a]anthracene with a monoclonal antibody against CYP1A1 showed a higher binding of the antibody in lower spinous cells than in basal cells. Differentiated keratinocytes had a higher AHH activity than germinative cells, but both cell types were susceptible to CYP induction. The lowest AHH activities were observed in basal and in low Ca^2+^ (8 × 10^−5^ M) cultured keratinocytes, and the highest activities in differentiated cell and in keratinocytes cultured in the presence of high Ca^2+^ (2 × 10^−3^ M), suggesting that constitutive AHH expression is an event of the differentiation program that can be modulated in vitro by Ca^2+^ (Guo et al. 1990).

*Inhibition*. AHH in rat epidermal microsomes was inhibited by very low concentrations of the dermatology widely used antifungal agent clotrimazole (IC_50_ 1.2–2.5 × 10^−7^ M) (Mukhtar et al. 1984), and AHH and EROD activities were inhibited by relatively low concentrations (IC_50_ 4–13 × 10^−5^ M) of nordihydroguaiaretic acid demonstrating that the use of the latter as diagnostic lipoxygenase inhibitor needs caution (Agarwal et al. 1991). Rat skin EROD was inhibited by the classical CYP inhibitors 5,6-benzoflavone, 7,8-benzoflavone and metyrapone (Moloney et al. 1982a). Ketoconazole and miconazole, also imidazole antifungal agents, inhibit retinoic acid metabolism by rat epidermal microsomes (IC_50_ 6.5 × 10^−7^ and 10^−5^ M, respectively) (Vanden Bossche et al. 1988). Ketoconazole may be effective in maintaining biologically active levels of retinoic acid in epidermal cells.

### Non-CYP oxidoreductases

#### Monoamine oxidase (MAO)

In the rat skin, MAO is present. It metabolizes serotonin (a substrate preferentially oxidized by MAO A) and is inhibited by pargyline (a selective inhibitor of MAO B) (it was not defined whether rat skin contains MAO A or MAO B or both) (Semak et al. 2004).

#### Xanthine oxidase

The carcinogenic air pollutant 2-nitrofluorene was reduced to the corresponding amine by the microsomal fraction of rat skin with NADPH and by the cytosolic fraction with 2-hydroxypyrimidine or 4-hydroxypyrimidine or hypoxanthine under anaerobic conditions. The cytosolic activity was much higher than that of the microsomes. The 2- or 4-hydroxypyrimidine-linked nitroreductase activity was inhibited by oxypurinol and 8-(3-methoxy-4-phenylsulfinylphenyl)-pyrazolo[1,5-a]-1,3,5-triazine-4(1H)-one (BOF-4272), inhibitors of xanthine oxidase. Upon partial purification, the fractions containing xanthine oxidase exhibited a marked 2-hydroxypyrimidine-linked nitroreductase activity and showed immunoreactivity against anti-rat xanthine oxidase. Thus, xanthine oxidase plays a major role in the nitroreduction of 2-nitrofluorene in the rat skin (Ueda et al. 2003). Nitroreduction is a key metabolic reaction in the activation of nitroarenes to mutagens (Vance et al. 1987).

#### Peroxidase

A cyanide and azide-inhibitable peroxidase was observed in and partially purified from neonatal (3–6 days old) rat skin, which had activity toward pyrogallol, hydroquinone, *p*-cresol, catechol, benzidine, 3,3′-dimethoxybenzidine, tetramethylbenzidine, and *p*-phenylenediamine (Strohm and Kulkarni 1986). These peroxidase-mediated reactions may constitute an alternative pathway to CYP-mediated metabolism in the rat skin.

#### Alcohol dehydrogenase (ADH) and aldehyde dehydrogenase (ALDH) (see also Table 2)

ADH and ALDH are active in rat skin. ADH1, ADH3, ALDH1, and ALDH2 proteins were expressed constitutively. ADH2 was not detected. Immunohistochemistry showed predominant localization of ADH and ALDH in the epidermis, sebaceous glands, and hair follicles. The ADH inhibitor 4-methylpyrazole (1 mM) reduced ethanol oxidation in the rat skin to ≈30–40 % of the control activity (Cheung et al. 2003b).

**Table 2:** Representative non-CYP-mediated oxidoreductase activities in skin of various mammalian species

Model substrate (for)	Human	Rat	Mouse	Guinea pig
Benzydamine (FMO)	+			
Methimazol (FMO)			0.35^a^	
Thiobezamide (FMO)			0.32^a^	
Arachidonic acid (COX)	23.5 ± 8.7^b^			
Ethanol^c^ (ADH)	0.3–0.4^d^	2.06^d^	1.1–1.2^d^	0.6^d^
2,6-Dichlorophenolindophenol (NQR)	~375^d^		159 ± 20^d^	
Menadione (NQR)	7–10^d^	+		

More examples and references in the text; only constitutive activities

*ADH* alcohol dehydrogenase, *COX* cyclooxygenase, *FMO* flavin-dependent monooxygenase, *NQR* NADH/NADPH quinone reductase

^a^nmol product/mg microsomal protein/min

^b^pg PGE2 formed/mg microsomal protein/min

^c^Beside ethanol, ADH activity is shown in human skin for 2-butoxyethanol > 2-phenoxyethanol > ethylene glycol > 2-ethoxyethanol as substrates

^d^nmol product/mg cytosolic protein/min

ADH3 mRNA was expressed in the epidermis of the rat embryo (embryonic day 12.5) and in the adult rat, ADH4 only in the adult rat, restricted to the stratum basale and partly to the stratum spinosum and granulosum (Westerlund et al. 2005).

Lockley et al. (2005) observed ADH/ALDH activities in whole and dermatomed rat skin cytosolic fractions with rates that were greatest for 2-butoxyethanol > 2-phenoxyethanol > ethylene glycol > 2-ethoxyethanol > ethanol and dermatomed skin cytosol with approximately twice the specific activity of whole rat skin. This suggests that ADH and ALDH are localized in the epidermis that constitutes more of the protein in dermatomed skin than whole-skin cytosol. Disulfiram completely inhibited alcohol and glycol ether metabolism in the skin cytosolic fractions. ADH1, ADH3, and ADH4 proteins predominate in rat skin and are responsible for metabolizing glycol ethers. Following multiple topical exposure, ethanol metabolism increased the most following ethanol treatment and 2-butoxyethanol metabolism increased the most following 2-butoxyethanol treatment. Ethanol and 2-butoxyethanol may induce specific ADH and ALDH isoforms that preferentially metabolize short-chain alcohols (i.e., ADH1 and ALDH1) and longer-chain alcohols (i.e., ADH3, ADH4, and ALDH1), respectively. Treatment with a general inducing agent such as dexamethasone enhanced ethanol and 2-butoxyethanol metabolism, suggesting the induction of multiple ADH isoforms.

#### NAD(P)H:quinone reductase (NQR)

NAD(P)H:quinone reductase (also called NADH/NADPH quinone oxidoreductase NQO; DT-diaphorase) is present in rat skin. Its activity was abolished by the classical inhibitor of NQR dicoumarol and strongly induced upon oxidative stress, both, in terms of increased mRNA as well as increased enzyme activity (using menadione as substrate) (Rees et al. 1994).

### Hydrolases (see also Table 3)

#### Epoxide hydrolase (EH)

Skin microsomes from untreated rats exhibited depending on the substrate used 0.3–1.7 % (Bentley et al. 1976) and about 6 % (Mukhtar and Bickers 1981) of the rat liver microsomal EH-specific activity. The sequence of the rate of hydration phenanthrene-9,10-oxide > 7-methylbenz[a]anthracene-5,6-oxide ~ benz[a] anthracene-5,6-oxide ~ benzo[a]pyrene (BP)-4,5-oxide > 3-MC-11,12-oxide > dibenz[a,h] anthracene-5,6-oxide was the same as in the rat liver (Bentley et al. 1976). The specific activity was among the lowest activities determined in 26 organs of the rat and within the skin was highest in the epidermis, followed by dermis and then by subcutis (Oesch et al. 1977). The specific activity was measured using BP-4,5-oxide as substrate, but it was demonstrated that the same microsomal EH enzyme was responsible for the hydration of this and of other standard substrates of microsomal EH such as styrene-7,8-oxide (Oesch and Bentley 1976). The skin microsomal EH had very similar properties as the liver microsomal EH except for a much lower amount in the skin (Oesch et al. 1977). EH activity toward BP-4,5-oxide and *trans*-stilbene oxide was observed in the rat skin microsomes and toward *cis*-stilbene oxide in the rat skin cytosol (Pham et al. 1989). Microsomal EH was relatively potently (IC_50_ 0.1 mM) inhibited by clotrimazole (Mukhtar et al. 1984).For the cytosolic EH, the specific activity in male rat skin was 1.6-fold higher than in female skin. The specific activity of cytosolic EH in skin was about 4 times lower than in liver cytosol that of microsomal EH 10- to 30-fold lower in skin compared with the liver.

**Table 3:** Representative xenobiotic hydrolase activities in skin of various mammalian species

Substrate (for)	Human	Rat	Mouse	Pig
Phenyl acetate (E)		1,130 ± 25^c^ (micr)		
		3,440 ± 1400^d^ (cytos)		
Naphthyl acetate (E)	90 ± 6^a^ (micr)	1,500 ± 70^a^ (micr)		28 ± 3^a^ (micr)
	47 ± 3^a^ (cytos)	280 ± 1^a^ (cytos)		85 ± 9^a^ (cytos)
Para-nitrophenyl acetate (E)	91 ± 4^a^ (micr)	2,100 ± 100^a^ (micr)		46 ± 6^a^ (minipig micr)
	45–86^a^ (cytos)	380 ± 20^a^ (cytos)		155 ± 18^a^ (minipig cytos)
		188 ± 30^a^ (S9)		
Para-nitrophenyl butyrate (E)		460 ± 127^a^ (S9)		
4-Methylumbelliferone acetate (E)	0.5^a^ (S9)			
Carbaryl (E)		0.2 ± 0.03^c^ (micr)		+
		0.5 ± 0.12^d^ (cytos)		
Fluroxypyr methyl ester (E)		1400^b^ (homogenate)		
Fluroxypyr methylheptyl ester (E)		490^b^ (homogenate)		
Fluazifo *p*-butyl (E)		20 ± 1.5^c^ (micr)		
		400 ± 60^d^ (cytos)		
Methylparaben (E)	~400^a^ (micr)			~390^a^ (minipig micr)
	~550^a^ (cytos)			~570^a^ (minipig cytos)
Ethylparaben (E)	~420^a^ (micr)			~400^a^ (minipig micr)
	~520^a^ (cytos)			~440^a^ (minipig cytos)
Propylparaben (E)	~100^a^ (micr)			~150^a^ (minipig micr)
	~210^a^ (cytos)			~380^a^ (minipg cytos)
Butylparaben (E)	~80^a^ (micr)			~100^a^ (minipig micr)
	~150^a^ (cytos)			~140^a^ (minipig cytos)
Benzylparaben (E)	~50^a^ (micr)			~30^a^ (minipig micr)
	~180^a^ (cytos)			~170^a^ (minipig cytos)
Ethyl nicotinate (E)		27.3 ± 11.7^a^ (S9)	6.46 ± 1.14^a^ (S9)	
Prodrug esters (E)	+ (Many)	+ (Many)	+ (Many)	+ (Alkylazacycloalkan-2-one prodrug esters)
Phenanthrene 9,10-oxide (mEH)	2.53^a^ (micr)	0.808^a ^(micr)	1.58^a^ (micr)	
Benz[*a*]anthracene 5,6-oxide (mEH)	0.526^a^ (micr)	0.11^a ^(micr)	0.129^a^ (micr)	
Benzo[*a*]pyrene 4,5-oxide (mEH)	0.175–0.447^a^ (micr)	0.12–0.16^a^ (micr)	0.111–0.172^a^ (micr)	
7-Methylbenz[*a*]anthracene 5,6-oxide (mEH)	0.384^a^ (micr)	0.119^a^ (micr)	0.159^a^ (micr)	
3-Methylcholanthrene 11,12-oxide (mEH)	0.059^a^ (micr)	0.004^a^ (micr)	0.023^a^ (micr)	
Dibenz[*a,h*]anthracene 5,6-oxide (mEH)	0.021^a^ (micr)	0.0015^a^ (micr)	0.003.5^a^ (micr)	
Styrene 7,8-oxide		0.15 ± 0.03^a^ (micr)		
*Cis*-stilbene oxide		0.11–0.0160^a^ (micr)		
*Trans*-stilbene oxide (sEH)		0.027–0.043^a^ (cytos)		

More examples and references in the text; only constitutive activities

*S9* 9,000*g* supernatant fraction, *cytos* cytosol, *E* esterase, *FPMH* fluroxypyr methylheptyl ester, *mEH* microsomal epoxide hydrolase (EH1; EPH1), *micr* microsomes, *sEH* soluble epoxide hydrolase (EH2; EPH2)

^a^nmol product/min/mg protein

^b^µmol/min/g of tissue

^c^nmol/min/g microsomal fraction

^d^nmol/min/g cytosolic fraction

#### Esterase/amidase

Many prodrug esters of cytostatic drugs such as esters of temozolomide (Suppasansatorn et al. 2006) as well as many other compounds used as prodrugs for topical application such as ethylnicotinate (Sugibayashi et al. 1999; Rittirod et al. 1999) are effectively hydrolyzed by rat skin esterase (faster than by mouse skin esterase).

Determination of the transdermal metabolism of the herbicides fluroxypyr methylheptyl ester (FPMH) and fluroxypyr methyl ester (FPM) during penetration through rat (and human) skin in vitro showed that both FPM and FPMH were completely metabolized during their passage through skin. The only metabolite observed was the hydrolysis products, fluroxypyr (FP). Similar metabolic profiles were produced during the transdermal metabolism of FPM and FPMH in previously frozen rat skin, indicating the robust nature of the esterase enzymes involved. The authors conclude that after skin contact with FPM or FPMH, systemic exposure (to rat and human) is likely to be to the acid metabolite, FP, only and not to the parent ester (Hewitt et al. 2000a). In crude rat, whole-skin homogenate FPM and FPMH were extensively metabolized to the acid metabolite, FP. In no instance were any other metabolites detected. FPM was essentially hydrolyzed completely within 1 h. In FPMH incubations, there was still parent ester present after 24 h at all concentrations tested. The kinetics of hydrolysis of the two esters was different: *V*_max_ was approximately threefold greater for FPM than FPMH (1,400 and 490 μmol FP/min/g of tissue, respectively); however, *K*_m_ values were very similar, 251 and 256 µM, respectively. Taken together with the data presented above (Hewitt et al. 2000a), the authors conclude that no parent ester will pass through the skin in vivo, only the metabolite, fluroxypyr, and that therefore first-pass metabolism will be completed before these esters reach the systemic circulation (Hewitt et al. 2000b).

Carboxylesterase, especially the rat isozyme hydrolase A, is expressed in rat skin. The hydrolysis of *para*-nitrophenyl acyl derivatives and caproyl-propranolol was 20-fold lower in the S9 fraction of skin homogenate than in liver microsomes (Imai et al. 2013). Hydrolysis efficiency (equivalent to the intrinsic metabolic clearance in the whole organism: *V*_max_:*K*_m_ through rearrangement of the Michaelis–Menton equation, assuming substrate concentration is below *K*_m_) for the prototypical carboxylesterases substrates naphthyl acetate and para-nitrophenyl acetate was higher in rat skin than in rat plasma, but lower than in rat liver. The esterase efficiency of rat skin microsomes (580–1,100 min^−1^ mg^−1^) was two to three orders of magnitude higher than human (1.3–4.2 min^−1^ mg^−1^) and minipig skin microsomes (1.2–4.2 min^−1^ mg^−1^). Rat skin cytosol (80–100 min^−1^ mg^−1^) was 2- to 10-fold more efficient than human (2.4–67 min^−1^ mg^−1^) or minipig cytosol (18–61 min^−1^ mg^−1^) (Prusakiewicz et al. 2006).

The aryloxyphenoxypropionate Fluazifop-butyl (butyl-(*R*)-2-(4-((5-(trifluormethyl)-2-pyridyl)oxy)phenoxy)propionat) was hydrolyzed by rat skin cytosol as well as microsomes, but with markedly different *V*_max_: 20 pmol/min/g microsomes; 400 pmol/min/g cytosol. Inhibition by paraoxon and bis-nitrophenol phosphate indicated the involvement of carboxylesterases. Carbaryl was also hydrolyzed in both subcellular fractions by carboxylesterases. Phenylacetate was also hydrolyzed by both microsomal and cytosolic skin fractions. Hydrolysis involved arylesterases in the microsomes and carboxylesterases in the cytosol. However, paraoxon was not hydrolyzed by the rat skin (McCracken et al. 1993).

The esterase distribution in rat skin studied microphotographically using fluorescein-5-isothiocyanate diacetate showed a higher enzyme concentration in the epidermal cells and near hair follicles than in the dermis (Sugibayashi et al. 1999). In a permeation study of caproyl-propranolol in rat full-thickness and stripped skin using a flow-through diffusion cell, caproyl-propranolol was easily partitioned into the stratum corneum and retained in the stratum corneum and in viable epidermis and dermis. Caproyl-propranolol could barely be detected in the receptor fluid after application to either full-thickness or stripped skin. However, its hydrolysis product propranolol was detected in receptor fluid. Permeation of caproyl-propranolol was markedly decreased under carboxylesterase inhibition, indicating that the net flux of caproyl-propranolol is dependent on its conversion to propranolol (reminiscent of what was discussed above for FPMH and FPM compared with their hydrolysis product FP).

### Conjugating enzymes

#### Glutathione *S*-transferase (GST) (see also Table 4)

Glutathione *S*-transferase (GST) was found in the rat skin cytosol where its specific activity (activity per amount of enzyme) for the broad-spectrum substrate 1-chloro-2,4-dinitrobenzene (CDNB) was 15 % of that in the liver (Mukhtar and Bickers 1981). Rat skin microsomes had an approximately tenfold lower specific activity toward CDNB compared with rat skin cytosol (Raza et al. 1991). Cytosolic GST activity toward CDNB increased only slightly with increasing age of the rats (from 48.3 to 65.7 nmol/mg protein/min between the age of 5 until 67 days) (Raza et al. 1991), which partly differs from the observation of Jewell et al. (2000) that cutaneous activity toward CDNB was higher in adult rat > pig > mouse > neonatal rat. For *cis*-stilbene oxide, the GST-specific activity in the rat skin was surprisingly high (half as high as in the liver cytosol) (Pham et al. 1989). A single topical application of clotrimazole resulted in 80 % induction of the rat epidermal GST activity toward CDNB (Mukhtar et al. 1984). Pendlington et al. (1994) reported that the rat skin GST was almost exclusively located within the sebaceous cells.

**Table 4:** Representative glutathione *S*-transferase (GST) activities in skin of various mammalian species

Substrate (for)	Human	Rat	Mouse	Pig
CDNB (broad spectrum)	20–451^a^ (cytos)	52–247^a^ (cytos)	53.8–106^a^ (cytos)	129 ± 10^a^ (cytos)
	ca. 3^a^ (micr)	5.35 ± 0.92^a^ (micr)		
Styrene 7,8-oxide	3.91 ± 0.28^a^ (cytos)	4.80 ± 0.33^a^ (cytos)	5.31 ± 0.33^a^ (cytos)	
*Cis*-stilbene oxide		1.59–1.65^a^ (cytos)		
Benz[*a*]pyrene 4,5-oxide	0.85 ± 0.06^a^ (cytos)	0.90 ± 0.05^a^ (cytos)	1.22 ± 0.09^a^ (cytos)	
3-Methylcholanthrene-11,12-oxide	0.17–0.46^b^ (cytos)		0.62–1.10^b^ (cytos)	
Ethacrynic acid (GST Pi)	5.02 ± 0.41^a^ (cytos)	3.05 ± 0.22^a^ (cytos)	15.5 ± 1.5^a^ (cytos)	
4-Hydroxynonenal (GST A4-4)	ca. 20^a^ (cytos)	<0.02^c^ (cytos)		
Bromosulphthalein	bd	bd	bd	

More examples and references in the text; only constitutive activities

*bd* below detection, *CDNB* 1-chloro-2,4-dinitrobenzene, *cytos* cytosol, *GST* glutathione *S*-transferase, *micr* microsomes, *PAH* *epox* polycyclic aromatic hydrocarbon K-region epoxides

^a^nmol product/mg protein/min

^b^nmol product/mg protein/5 min at 22° C

^c^nmol product/mg protein/5 min at 37° C

^d^µg product/mg protein/min

The levels of the cofactor necessary for GST activity, glutathione (GSH), in the rat skin varied widely from below detectability (Yarat et al. 2001) to 73 ± 28 nmol glutathione/mg skin protein (Tunali et al. 2004), 53 ± 3 nmol glutathione/g skin tissue (Korac and Buzadzic 2001), 11.2–12.7 nmol glutathione “per 50 mg skin” (Rees et al. 1994), 16.15 ± 2.18 nmol glutathione “per 50 mg tissue” (Adamson et al. 1996), 30.3 ± 2.5 nmol glutathione/cm^2^ in the young adult (26 day old) and 91.3 ± 3.8 nmol glutathione/cm^2^ in the neonatal rat (Jewell et al. 2000), 16.5 ± 5.7 nmol glutathione/mg protein in the epidermis of the hairless rat (Romeu et al. 2002), and 20.7 ± 3.0 nmol glutathione “per 2 million fibroblasts grown for 3 days with F12/DMEM” (Adamson et al. 1996).

Mu and Pi (predominantly Pi), but not alpha classes of GST, were identified in rat skin cytosol (Raza et al. 1991) (GST families are called “classes and are designated by Greek letters). These GST isozymes expressed activities toward CDNB, BP 4,5-oxide, styrene 7,8 oxide, leukotriene A4, and ethacrynic acid, while no activities toward bromosulfophthalein and cumene hydroperoxide were observed.

7α- and 7β-Hydroperoxycholest-5-en-3b-ols (cholesterol 7α- and 7β-hydroperoxides) are aging markers in the rat skin (Ozawa et al. 1991). These toxic hydroperoxides are reduced by alpha-class GSTs, composed of the subunits Ya–Ya and Ya–Yc, and by the selenium-containing peroxidase GSH Px (Se-GSH Px) in rat liver cytosol (apparent specific activity toward the cholesterol hydroperoxides GSTs Ya–Ya > Ya–Yc ≫ Se–GSH Px, but approximately equal *V*_max_/*K*_m_ values). Rat skin had very low concentration of Se-GSH Px, and no GST bearing the subunit Ya, possibly resulting in the accumulation of cholesterol 7-hydroperoxides in the skin. From rat skin cytosol, GSTs Yc–Yc, Yb1–Yb1, Yb1–Yb2, Yb2–Yb2, and Yp–Yp were purified to homogeneity and identified with the corresponding GSTs from liver and kidney. The GSTs accounted for 0.23 % of total skin cytosolic protein, and the most abundant isoform of skin GSTs was Yb2–Yb2, followed by Yc–Yc, Yp–Yp, Yb1–Yb1, and Yb1–Yb2 in decreasing order (Hiratsuka et al. 1997).

The alpha-class rat GST A4-4 catalyzes the GSH conjugation of the toxic 4-hydroxy-2(*E*)-nonenal (HNE), non-enzymatically formed from ω-6 polyunsaturated fatty acid residues of lipids by lipid peroxidation. While enzyme, Western blot, and immunohistochemical analyses indicated that rat skin cytosol contained no detectable level of rGST A4-4, rats irradiated with UVB markedly expressed rGSTA4-4 in the skin (purified to homogeneity and identified by reverse-phase partition HPLC and by amino acid sequence analysis). The specific activity toward HNE was one-fifth of that in the liver after a single dose of 24,000 mJ/cm^2^. Immunohistochemistry demonstrated the selective expression of rGSTA4-4 in epidermis and sebaceous glands localized in dermis after UVB irradiation (Hiratsuka et al. 1999).

#### UDP-glucuronosyltransferase (UGT) (see also Table 5)

Rat whole-skin microsomes exhibit UGT activity toward “group 1” substrates that were defined as those whose glucuronidation was increased by pretreatment of the rats with 3-MC but not after pretreatment with phenobarbital, which include 1-naphthol, *N*-hydroxy-2-naphthylamine and 3-hydroxy-BP (Bock et al. 1980). Glucuronidation of BP-7,8-dihydrodiol, the immediate precursor of the most genotoxic derivative of BP, the 7,8-dihydrodiol 9,10-epoxide, is unproportionately low in the rat skin (Bock et al. 1980). UGT activity toward 1-naphthol (Moloney et al. 1982b) and bilirubin (Pham et al. 1989) was observed in rat skin microsomes. The apparent *K*_m_ values were considerably lower than those for rat liver UGT. The specific activity in rat skin microsomes was about 10–50 % (Moloney et al. 1982b; Pham et al. 1989) of that in rat liver microsomes. Pretreatment with 3-MC resulted in small increases in cutaneous UGT activities (Moloney et al. 1982b). Testosterone glucuronidation was not detected in rat skin microsomes (Pham et al. 1989). Dressler and Appelqvist (2006) tentatively showed that rat skin metabolized acetylaminophenol (APAP, paracetamol, acetaminophen) to the glucuronide.

**Table 5:** Representative UDP-glucuronosyl (UGT) and sulfotransferase (SULT) activities in skin of various mammalian species

Substrate (for)	Human	Rat	Mouse	Pig
4-Methylumbelliferone (UGT)	1.3 ± 0.2^a^		11.1 ± 9.65^b^	
Bilirubin (UGT)	+	15–23^a^		
4-Hydroxybiphenyl (UGT)		<0.08^a^		
7-Hydroxycoumarin (UGT)				<0.333 ± 0.032^c^
1-Naphthol (UGT)		2.5–13^a^		
3-Hydroxybenzo[*a*]pyrene (UGT)		0.08 ± 0.01^a^		
Testosterone (UGT)		<0.09^a^		
Salicylic acid (UGT)	0.0062–0.016^a^			
Acetaminophen (UGT)		+ (Tentative)		+ (Tentative)
Acetaminophen (SULT)		+ (Tentative)		+ (Tentative)
7-Hydroxycoumarin (SULT)				<0.183 ± 0.029^c^
4-Nitrophenol (SULT)	Traces—0.18 ± 0.02^d,e^			
Minoxidil (SULT1A1)	0.21 ± 0.02^d^	0.006^f^		
Dopamine (SULT)	0.60 ± 0.05^d^			

More examples and references in the text; only constitutive activities

*UGT* UDP-glucuronosyltransferase, *SULT* sulfotransferase

^a^nmol product/min/mg microsomal protein

^b^ nmol/min/g of skin

^c^ nmol/h/g of skin analyzed in the culture medium

^d^ nmol/h/mg “high-speed supernatant” protein

^e^ pmol/min/mg protein (sum of 7,500*g* supernatant + medium)

^f^ nmol product/min/mg cytosolic protein

Rat skin fibroblasts possess UGT activities, which are protective against the genotoxicity of BP (Vienneau et al. 1995), the tobacco-specific nicotine-derived nitrosamine ketone (NNK) (also known as 4-[methylnitrosamino]-1-[3-pyridyl]-1-butanone) (Kim and Wells 1996) as well as phenytoin (Kim et al. 1997) by sequestration into non-toxic pathways.

The concentration of UDP-glucuronic acid, the cofactor required for UGT activity, was reported as 0.08 mM “in skin cellular water” and 28 ± 2 nmol/g wet tissue of the neonatal rat skin (Hardingham and Phelps 1968).

#### Sulfotransferase (SULT)

SULT activity toward minoxidil was observed in the cytosolic fraction of rat skin. The thermostability and inhibition by the little specific aryl (phenol) SULT inhibitors 2,6-dichloro-4-nitrophenol and para-nitrophenol with good inhibitory activity for SULT 1A1 (Wang et al. 2009) and lack of inhibition by low concentrations of dopamine and tyramine (substrates of SULT1A3) suggested that SULT1A1 is responsible for the observed activity (Wong et al. 1993). The enzymatic transfer of ^35^S from sodium ^35^sulphate to minoxidil was also demonstrated, suggesting that the rat skin is capable of synthesizing 3′-phosphoadenosine-5′-phosphosulphate (PAPS), the required cofactor of SULT. Thus, it is conceivable that the metabolism-dependent action of minoxidil as a promoter of hair growth could be carried out by the skin itself (Wong et al. 1993).

Dressler and Appelqvist (2006) tentatively showed that rat skin metabolized acetylaminophenol (paracetamol) to the sulfate.

#### *N*-Acetyltransferase (NAT) (see also Table 6)

After uncovering the full expression of cutaneous serotoninergic and melatoninergic systems in the skin, Semak et al. (2004) characterized serotonin metabolism in the rat skin and found that serotonin undergoes acetylation in the presence of acetyl coenzyme A. Inhibition of serotonin acetylation with “Cole bisubstrate inhibitor” showed that rat skin expresses arylalkylamine and arylamine *N*-acetyltransferase activities and that serotonin is acetylated by both of them to generate the precursor of melatonin.

**Table 6:** Representative *N*-acetyltransferase (NAT) activities^a^ in skin of various mammalian species

Substrate	Human	Rat	Mouse	Pig
Para-aminobenzoic acid	0.45 ± 0.17	3–6	+	
Para-phenylenediamine	0.41–3.68	+ (Tentative)		+ (Tentative)
Para-aminophenol	+	+ (Tentative)		+ (Tentative)
Para-toluidine	0.63–3.03			
2-Aminofluorene		ca. 1	ca.3	

More examples and references in the text; only constitutive activities

^a^nmol product/mg cytosolic protein/min

With respect to purely xenobiotic substrates, two NATs have been discovered: NAT1 and NAT2 [Walraven et al. (2006) identified a third rat NAT gene, NAT3]. NAT1 was tentatively identified in the rat skin to transform para-aminophenol to acetylaminophenol (paracetamol) (Dressler and Appelqvist 2006). NAT1 is notoriously labile, which—at least in part—explains high variability in NAT1 activity determinations (Fabian et al. 2013).

## Xenobiotic-metabolizing enzymes in the mouse skin

### Cytochromes P450 (CYP)

#### CYP transcript expression

In the mouse skin, the expression of several *Cyp* genes was demonstrated on the RNA level. Determination of (CD-1) mouse cutaneous RNA levels by real-time polymerase chain reaction showed transcripts arising from *Cyp4f13* and *4f16*(most abundant); *Cyp4f18* and *4f39* (intermediate); *Cyp4f14, 4f17,* and *4f37* (low); and from *Cyp4f15* and *Cyp4f40* (highly variable or too low to measure in some animals) (Du et al. 2009). Also, the expression of the *Cyp1a1, Cyp1b1, Cyp2e1* genes was observed in (C57BL/6J) mouse skin (Flowers et al. 2011).

#### CYP protein expression

Pohl et al. (1976) demonstrated by CO/dithionite-reduced minus dithionite-reduced difference spectroscopy the presence of CYP protein as well as increases in CYP content of mouse skin microsomes 24 and 72 h after topical treatment with TCDD.

Pendlington et al. (1994) observed constitutive CYP1A1/A2 and CYP2B1/B2 concentrated in the epidermis and sebaceous glands of the (MFl/h hairless) mouse. The cell fractions enriched by density gradient centrifugation in basal and sebaceous cells contained high levels of CYP1A1/A2, which was induced approximately tenfold following β-naphthoflavone pretreatment.

The treatment with dexamethasone resulted in an increased immunoreactivity (1.8–13.9 times) determined using antibodies against purified CYPs 1A1/2, 2B1/2, 2E1, and 3A. Using antibody against CYP2B1/2, the treatment with dexamethasone resulted in an increased reactivity in the suprabasal layer of the epidermis and in the hair follicles (Jugert et al. 1994).

Saarikoski et al. (2005a, b) reported the presence of the novel CYP2S1 in the mouse skin as well as its strong induction by topical treatment with CDNB.

#### CYP catalytic activities (see also Table 1)

Mouse (Swiss-Webster CD-1) skin microsomes catalyzed the hydroxylation of BP and aniline and the deethylation of 7-ethoxycoumarin. The enzyme activities did not respond to topical application of 3-MC. However, 24 h after topical application of TCDD, microsomes from skin had 50 % greater AHH and EROD activity, and 4- to 8-fold greater activity after 72 h compared to the untreated control (Pohl et al. 1976).

The topical application of corticosteroids used in the therapy of human dermatological disorders induces AHH activity in mouse skin (Briggs and Briggs 1973), suggesting that also in dermatology, induction of human cutaneous AHH by corticosteroids may be associated with their therapeutic effect. Topical treatment of mice (NMRI) with dexamethasone resulted in the induction of EROD (2.3 times), PROD (19.2 times), para-nitrophenol hydroxylase (7.5 times), and erythromycin *N*-demethylase (2.2 times), activities catalyzed preferentially by CYP 1 family, 2B1, 2E1, and 3A, respectively (Jugert et al. 1994).

Das et al. (1985) observed in (SKH hairless) mouse skin low, but significant AHH activity (10.56 ± 0.12 pmol 3-hydroxy-BP formed/min/mg microsomal protein) and ECOD (12.39 ± 0.10 pmol 7-hydroxycoumarin formed/min/mg microsomal protein) activities (compared with 625.50 ± 5.63 and 1,198.6 ± 37.8, respectively, in the liver as determined in the same mice in the same study). The activities in the skin were further reduced by chronic UVB irradiation (to 4.71 ± 0.09 and 4.30 ± 0.07, respectively) and still further reduced in the skin tumors produced by the UVB irradiation (to 1.89 ± 0.04 and 2.56 ± 0.04, respectively). (USP) Coal tar (which contains polycyclic aromatic hydrocarbons) treatment (single topical application of 1 mL/100 g) of unirradiated controls increased both activities about 3.3-fold.

Storm et al. (1990) compared CYP-dependent activities in the mouse skin with those in the skin of the guinea pig and human. AHH and ECOD activities in (Sencar) mouse skin (3.35 ± 0.07 and 10.4 ± 1.4 pmol/min/mg protein, respectively) were similar as in (hairless) guinea pig skin (2.51 ± 0.35 and 3.8 ± 2.7 pmol/min/mg protein, respectively), but much higher than in human skin (0.24 ± 0.08 pmol/min/mg protein and below detection, respectively).

In a comparative study, Rolsted et al. (2008) found in mouse skin microsomes the following activities (expressed in pmol metabolites/h/mg protein): EROD 95.4 ± 4.2, PROD below detection (LOQ 1.87 pmol), tolbutamide 4-hydroxylation (prototypical for CYP2C9) below detection, bufuralol 1-hydroxylation (prototypical for CYP 2D6) 9.23 ± 0.67, chlorzoxazone 6-hydroxylation (prototypical for CYP2E1) 20.8 ± 0.5, midazolam 1-hydroxylation (prototypical for CYP3A) 8.70 ± 0.28.

Pendlington et al. (1994) observed constitutive EROD and PROD activities in mouse skin. A single topical application of β-naphthoflavone (200 µL of a 20-mg/mL solution in acetone) induced ECOD activity. However, a single topical application of phenobarbital (200 µL of a 20-mg/mL solution in ethanol) had no effect on PROD activity.

In (CD-1) mouse skin cells, Du et al. (2009) observed leukotriene B4 (LTB4) hydroxylation activity converting LTB4 to 20-OH-LTB4. This metabolic reaction represents an inactivation of LTB4, which is an innate mechanism to resolve tissue inflammation. Retinoic acid exposure induced microsomal LTB4 hydroxylation activity in mouse skin cells. The authors conclude from their data that 20-hydroxylation is the major CYP-dependent LTB4 inactivation pathway in mouse (and human) skin and that this retinoid-inducible metabolic pathway has capacity to modulate tissue levels of proinflammatory lipids.

Eugenol, a frequent sensitizer, does not sensitize CYP1A1 knockout mice, whereas the wild-type mice are sensitized to eugenol. This indicates that CYP1A1 is responsible for generating from eugenol the ultimately antigenic metabolite(s) (Basketter et al. 2008).

##### Localization

Mouse (hairless HRS/J) skin cells isolated after enzymatic digestion with trypsin and separated by metrizamide and Percoll gradient centrifugations showed the following EROD activities: in the fraction containing 80 % sebaceous cells 1,051 ± 221 pmol/min/mg DNA, in the fraction containing 50 % basal cells 175 ± 24 pmol/min/mg DNA, and in the fraction consisting predominantly of differentiated keratinocytes 11.8 ± 4.3 pmol/min/10^6^ cells. AHH in the former two fractions was 2,377 ± 1,164 and 735 ± 360 pmol/min/mg DNA, respectively. The activities were 2- to 10-fold increased by treatment with β-naphthoflavone (Coomes et al. 1983).

In four subpopulations of murine epidermal keratinocytes generated by Percoll gradient separation that differed in their stages of differentiation, non-induced per cell ECOD and EROD activities were the lowest in basal keratinocytes and progressively increased as the keratinocytes underwent differentiation. Treatment of dorsal mouse skin with 100 nmol of dibenz[a,c]anthracene increased EROD activities mostly in basal keratinocytes (greater than or equal to 1,850-fold!) and progressively less as the keratinocytes underwent differentiation (200-fold in the most differentiated fraction investigated, which contained spinous and granular keratinocytes) (Reiners et al. 1992).

##### Differentiation/age dependence

Keeney et al. (1998a, b) discovered a novel CYP in fetal (CD-1) mouse skin, CYP2B19, a new and specific cellular marker of late differentiation in skin keratinocytes. The onset of *Cyp2b19* expression coincided spatially (upper cell layer) and temporally (day 15.5) with the appearance of loricrin-expressing keratinocytes during the stratification stage of fetal epidermis. CYP2B19 is also present postnatally in the differentiated keratinocytes of the mouse epidermis, sebaceous glands, and hair follicles. *Cyp2b19* expression is tightly coupled to the differentiated (granular cell) keratinocyte phenotype in vivo and in vitro. In primary mouse epidermal keratinocytes, it is specifically upregulated and correlated temporally with calcium-induced differentiation and expression of the late differentiation genes *Loricrin* and *Profilaggrin*. Recombinant CYP2B19 metabolizes arachidonic acid, a normal constituent of cellular membranes and the precursor of biologically active lipids such as epoxyeicosatrienoic (EET) acids, hydroxyeicosatetraenoic (HETE) acids, leukotrienes, thromboxanes, and prostaglandins. Recombinant CYP2B19 generates 14,15- and 11,12-epoxyeicosatrienoic (EET) acids, and 11, 12-, and 15-hydroxyeicosatetraenoic (HETE) acids (20, 35, 18, 7, and 7 % of total metabolites, respectively) from arachidonic acid. The CYP2B19 metabolites 11,12- and 14,15-EET are endogenous constituents of murine epidermis and are present in similar proportions to those generated by the enzyme in vitro, suggesting CYP2B19 to be the primary enzymatic source of these EETs in murine epidermis (Keeney et al. 1998a, b). Normal fatty acid metabolism is critical to the permeability barrier function of the epidermis. Scaling epidermal lesions, abnormal keratinocyte differentiation, and chronic hyperproliferation are associated with abnormal levels of fatty acids including arachidonic acid (Keeney et al. 1998a, b).

Williams and Woodhouse (1995, 1996) observed that in the (C57BL/6 J) mouse skin ECOD, EROD activities were markedly lower in senescent skin while aldrin epoxidase activities remained constant with increasing age. Cutaneous NADPH-CYP reductase (determined as NADPH cytochrome c reductase), a component of the mixed function mono-oxygenase system, significantly decreased with age (in contrast to the liver where it remained constant).

### Non-CYP oxidoreductases (see also Table 2)

#### Cyclooxygenase

Mouse skin microsomes metabolized *trans*-7,8-dihydroxy-7,8-dihydrobenzo[*a*]-pyrene (BP-7,8-diol) to the 7,10/8,9-tetrol and the 7/8,9,10-tetrol of BP in the presence of arachidonic acid. Indomethacin inhibited this arachidonic acid-dependent oxidation in mouse skin. From these facts, the authors concluded that this metabolic conversion was dependent on cyclooxygenase (prostaglandin synthase) (Sivarajah et al. 1981).

Irradiation of C57BL/6 mice and arylhydrocarbon receptor (AhR) knockout (KO) mice with 600 J/cm^2^ UVB led to the induction of Cox-2 (and Cyp1a1) mRNAs in wild-type but not in AhR KO mice demonstrating control of Cox-2 expression by AhR and the activation of the latter by UVB (Fritsche et al. 2007).

#### Alcohol dehydrogenase (ADH) and aldehyde dehydrogenase (ALDH)

In the skin of the mouse (BALB/c and CBA/ca), ADH1, ADH3, ALDH1, and ALDH2 were expressed, constitutively, as shown by Western blot. ADH2, present in the mouse liver and present in both, human skin and liver, was not detected in the mouse skin. ALDH3 was expressed, constitutively, in the skin of both strains of mouse, but was absent in the liver. Immunohistochemistry showed similar patterns of expression for ADH and ALDH in both strains of mouse skin sections, with localization predominantly in the epidermis, sebaceous glands, and hair follicles. ADH activity determined by ethanol oxidation was higher in the mouse skin (apparent *V*_max_ 1.07–1.21 nmol/mg protein/min) than in human skin (0.32–0.41). The ADH inhibitor 4-methyl pyrazole reduced ethanol oxidation in mouse skin in a concentration-dependent manner. 1 mM 4-methyl pyrazole reduced the activity to ≈30–40 % of the basal activity (Cheung et al. 2003b).

#### NAD(P)H:quinone reductase (NQR)

Merk et al. (1991) reported that in marked contrast to most other investigated xenobiotic enzymes, NQR (also called NADH/NADPH quinone oxidoreductase NQO; DT-diaphorase) in mouse skin (cytosol) was almost as active as in the liver (cytosol) (ratio 1.3 as compared with a ratio of 27.4 for EROD and 107 for AHH).

Reiners et al. (1992) observed NQR activity in four subpopulations of murine epidermal keratinocytes generated by Percoll gradient separation that differed in their stages of differentiation. Non-induced per cell NQR activities in the three less-differentiated murine keratinocytes subpopulations were very similar to one another and greater than the activities measured in the most differentiated subpopulation. Treatment of dorsal mouse skin with 100 nmol of dibenz[a,c]anthracene increased NQR almost threefold, which was similar in all four keratinocyte subpopulations.

### Hydrolases (see also Table 3)

#### Epoxide hydrolase (EH)

Das et al. (1985) observed in (SKH hairless) mouse skin low, but significant epoxide hydrolase activities (0.11 ± 0.01 nmol BP-4,5-dihydrodiol formed/min/mg microsomal protein) (compared with 2.35 ± 0.18 in the liver as determined in the same mice in the same study). The activities in the skin were not significantly changed by chronic UVB irradiation and not significantly changed in the skin tumors produced by the UVB irradiation.

Decker et al. (2012) very recently discovered EH3 (ABHD9), a first member of a new epoxide hydrolase family with high activity for fatty acid epoxides. Quantitative RT-PCR from mouse tissues indicated strongest EH3 expression in lung, skin, and upper gastrointestinal tract.

### Conjugating enzymes

#### Glutathione *S*-transferase (GST) (see also Table 4)

Pendlington et al. (1994) observed constitutive GST protein concentrated in the epidermis and sebaceous glands of the (MFl/h hairless) mouse. The GST activity toward the broad-spectrum substrate CDNB was induced about fourfold in both male and female phenobarbital-pretreated mice (more in males than in females).

Mu and Pi (predominantly Pi), but not alpha classes of GST protein, were identified in mouse skin cytosol (Raza et al. 1991). These GST isozymes were largely localized in sebaceous glands and expressed activities toward CDNB, BP 4,5-oxide, styrene 7,8 oxide, leukotriene A4, and ethacrynic acid, while no activities toward bromosulfophthalein and cumene hydroperoxide were observed.

Mouse (hairless HRS/J) skin cells isolated after enzymatic digestion with trypsin and separated by metrizamide and Percoll gradient centrifugations showed the following GST activities toward the broad-spectrum substrate CDNB: in the fraction containing 80 % sebaceous cells 1,399 ± 802 nmol per min/mg DNA, in the fraction containing 50 % basal cells 399 ± 190 nmol/min/mg DNA, and in the fraction consisting predominantly of differentiated keratinocytes 11.8 ± 4.3 nmol/min/10^6^ cells. The activities were twofold increased by treatment with β-naphthoflavone in the basal cell fraction, but not in the sebaceous cell fraction (Coomes et al. 1983).

Das et al. (1985) observed in (SKH hairless) mouse skin low, but significant GST activity (53.83 ± 0.91 nmol CDNB GSH conjugate formed/min/mg cytosolic protein) (compared with 1,506 ± 12 in the liver as determined in the same mice in the same study). The activities in the skin were increased by chronic UVB irradiation to 93.14 ± 2.13 and reduced back to control activities in the skin tumors produced by the UVB irradiation (54.8 ± 3.81).

Agarwal et al. (1992) showed the presence of a GST activity in the mouse skin, which converted leukotriene A4 methyl ester to the GSH conjugate leukotriene C4 methyl ester. Leukotriene C4 is the precursor of leukotriene D4 and leukotriene E4, cysteine-containing leukotrienes responsible for allergic and anaphylactic reactions in the skin. The specific activity in the mouse skin (12.3 ± 0.9 pmol/min/mg 100,000*g* supernatant protein) was lower than in the rat skin (20.2 pmol/min/mg protein), but higher than in the human skin (5.9 pmol/min/mg protein).

In a comparative study, Jewell et al. (2000) found that cutaneous GST activity toward CDNB was lowest in the mouse compared with higher activities in pig < rat < human.

#### UDP-glucuronosyltransferase (UGT)

Mouse (hairless HRS/J) skin cells isolated after enzymatic digestion with trypsin and separated by metrizamide and Percoll gradient centrifugations showed the following UGT activities toward the broad-spectrum substrate 4-methylumbelliferone: in the fraction containing 80 % sebaceous cells 39.3 ± 13.8 nmol/min/mg DNA, in the fraction containing 50 % basal cells 13.7 ± 0.8 nmol/min/mg DNA, and in the fraction consisting predominantly of differentiated keratinocytes 11.8 ± 4.3 nmol/min/10^6^ cells. The activities were not significantly increased by treatment with β-naphthoflavone (Coomes et al. 1983).

## Xenobiotic-metabolizing enzymes in the guinea pig skin

### Cytochromes P450 (CYP) (see also Table 1)

In the (hairless) guinea pig skin, the CYP-dependent AHH and ECOD activities were observed. Their activities (2.51 ± 0.35 and 3.8 ± 2.7 pmol/min/mg protein, respectively) were similar as in (Sencar) mouse skin (3.35 ± 0.07 and 10.4 ± 1.4 pmol/min/mg protein, respectively), but much higher than in human skin (0.24 ± 0.08 pmol/min/mg protein and below detection, respectively) (Storm et al. 1990).

Thiele et al. (1987) found CYP-dependent AHH activity and inducibility by benzanthracene (BA) in cultured guinea pig epidermal cells. Basal AHH activity in guinea pig epidermal cells was much higher than in human epidermal cells. Basal AHH activity in guinea pig epidermal cells was directly related to the labelling index and decreased to the original level between the 5th and 7th day of cell culturing. However, the induction ratio of AHH which reached its maximum when the number of cells began to rise (proliferation phase) remained high at day 7 of the cell culture.

Mukhtar et al. (1989) demonstrated in guinea pig skin microsomes leukotriene B4 omega-hydroxylase activity converting LTB4 to the 20-hydroxy metabolite. Product formation, which required NADPH and oxygen, was inhibited by carbon monoxide or 2-diethylaminoethyl-2,2-diphenylvalerate hydrochloride (SKF-525A) indicting that the activity was CYP-dependent.

The presence of CYP reductase, required for the monooxygenase activity of CYP, was demonstrated in the guinea pig skin by Young et al. (1997): Strong staining in cells of the sebaceous glands was associated with hair follicles, and pale staining in the epidermis and in cells of the hair follicles.

### Non-CYP oxidoreductases

#### Lipoxygenase

Examination of the metabolism of radioactively labelled arachidonic acid via the lipoxygenase and cyclooxygenase pathways and the metabolic conversions of radioactively labelled prostaglandin H2 in the epidermal and dermal layers of the guinea pig skin showed that arachidonic acid was metabolized preferentially via lipoxygenase to hydroxyeicosatetraenoic acid (HETE). Epidermis exhibited much higher lipoxygenase activities per milligram protein than the dermis. The study showed that guinea pig skin is a highly active site of arachidonic acid metabolism (Ruzicka and Printz 1982).

#### Cyclooxygenase (COX)

The studies discussed just above (under *Lipoxygenase*) also examined the metabolism of radioactively labelled arachidonic acid via the cyclooxygenase pathways in the epidermal and dermal layers of the guinea pig skin. Although arachidonic acid was metabolized preferentially via the lipoxygenase pathway, the major product of the cyclooxygenase pathway was prostaglandin D2 while prostaglandin E2 was formed in lesser amounts. Epidermis exhibited much higher activities of cyclooxygenase activity per milligram protein than the dermis (Ruzicka and Printz 1982).

#### Alcohol dehydrogenase (ADH) and aldehyde dehydrogenase (ALDH)

In the skin of the guinea pig (Dunkin-Hartley), ADH1, ADH3, ALDH1, and ALDH2 were expressed, constitutively, as shown by Western blot. ADH2, present in the guinea pig liver (and present in both, human skin and liver), was not detected in the guinea pig skin. ALDH3 (expressed, constitutively, in the human, rat, and mouse skin) was absent from the guinea pig skin. Immunohistochemistry showed similar localization patterns of ADH (ADH1 and ADH 3) and ALDH (ALDH 1 and ALDH 2) in guinea pig skin sections, predominantly in the epidermis, sebaceous glands, and hair follicles. ADH activity determined by ethanol oxidation was higher in the guinea pig skin (apparent *V*_max_ 0.6 ± 0.8 [!] nmol/mg protein/min) (Table 2) than in human skin (0.32–0.41). The ADH inhibitor 4-methyl pyrazole reduced ethanol oxidation in the guinea pig skin in a concentration-dependent manner. 1 mM 4-methyl pyrazole reduced the activity to 22 % of the control activity (Cheung et al. 2003b).

### Hydrolases

#### Esterase/amidase

Prodrug hydrolysis of ethyl, propyl, butyl, octyl, and decyl *O*-acyl esters of haloperidol by cutaneous esterases during permeation across full-thickness guinea pig skin was minimal, also across freshly excised guinea pig skin (the hydrolysis rates of ethyl and butyl haloperidol were greatest, those of octyl and decyl haloperidol; the two prodrugs with the longest ester chains were hydrolyzed at the slowest rates). Also, hydrolysis studies using a skin extract revealed only limited prodrug metabolism (9.56, 6.58, 9.17, 6.07, 3.56 % haloperidol liberated after 50 h from ethyl, propyl, butyl, octyl, and decyl *O*-acyl esters of haloperidol, respectively) while using a liver extract, hydrolysis of all prodrugs was rapid (74.5, 96.8, 95.2, 106.1, 22.9 % haloperidol liberated already after 3 h from ethyl, propyl, butyl, octyl, and decyl *O*-acyl esters of haloperidol, respectively). It was proposed that GGGX esterases (possessing the glycine–glycine–glycine-x motif), required for the hydrolysis of tertiary esters, were not present at a sufficiently high concentration within the guinea pig skin for substantial tertiary ester prodrugs hydrolysis to occur (Morris et al. 2009).

### Conjugating enzymes

#### Glutathione *S*-transferase (GST)

Ruzicka and Printz (1982) reported that the guinea pig skin had a very high activity of GST isomerizing prostaglandin H2 to prostaglandin D2. Both skin layers, epidermis and dermis, showed similar and very high activity of GSH-dependent prostaglandin H2/prostaglandin D2 isomerase. Prostaglandin D2 was virtually the only product formed by skin homogenates from prostaglandin H2. The authors conclude that the guinea pig skin is a highly active site of arachidonic acid metabolism.

## Xenobiotic-metabolizing enzymes in the pig skin

### Cytochromes P450 (CYP) (see also Table 1)

The conversion of parathione to paraoxon, a metabolic step usually attributed to CYPs, was reported by Chang et al. (1994) to occur in isolated perfused viable and anatomically intact porcine skin.

In a comparative study, Rolsted et al. (2008) found in minipig skin microsomes the following activities (expressed in pmol metabolites/h/mg protein): EROD 4.62 ± 0.54, PROD “not detected” (LOQ 1.87 pmol), tolbutamide 4-hydroxylation (prototypical for CYP2C9) 1.66 ± 0.49, bufuralol 1-hydroxylation (prototypical for CYP 2D6) 0.26 ± 0.03, chlorzoxazone 6-hydroxylation (prototypical for CYP2E1) “not detected” (LOQ 12.8 pmol), midazolam 1-hydroxylation (prototypical for CYP3A) 2.32 ± 0.21.

Jacques et al. (2010a) demonstrated in short-term skin cultures from ears of domestic pigs the biotransformation of [^14^C]-7-ethoxycoumarin to 7-hydroxycoumarin (1.3 ± 1.2 pmol/h/mg protein [equivalent to 4.50 ± 4.17 pmol/h/cm^2^ skin and equivalent to 56.15 ± 52.1 pmol/h/g skin tissue]). To appreciate the full monoxygenase activity of the pig skin, to this has to be added the formation of the secondary metabolites derived from this primary metabolite 7-hydroxycoumarin: Glucuronide 7.71 ± 0.73 pmol/h/mg protein (equivalent to 26.70 ± 2.54 pmol/h/cm^2^ skin and equivalent to 333.24 ± 31.75 pmol/h/g skin tissue) and sulfate 4.24 ± 0.66 pmol/h/mg protein (14.67 ± 2.29 pmol/h/cm^2^ and 183.04 ± 28.53 pmol/h/g tissue). These numbers refer to the metabolites analyzed in the culture medium. Minor amounts of metabolites were also found in the cultured skin tissue (after 48 h, 70 % of the radioactivity was recovered in culture media, 10 % in the skin, including unchanged 7-ethoxycoumarin).

Likewise, Jacques et al. (2010b) observed that ^14^C-benz[*a*]pyrene (BP) was extensively metabolized by pig ear skin microsomes and similarly by pig ear skin explant short-term cultures, to the primary oxidative metabolites, hydroxylated BPs (as well as extensively to BP-glucuronide and sulfate conjugates and to a lesser extent to BP-diols, BP-catechols, and BP-diones metabolites derived from primary oxidative metabolites).

### Hydrolases

#### Epoxide hydrolase (EH)

Jacques et al. (2010b) observed that ^14^C-benz[*a*]pyrene (BP) was metabolized by pig ear skin microsomes and similarly by pig ear skin explant short-term cultures, to BP-diols, and BP-diol-glucuronides, derived from it.

#### Esterase/amidase (see also Table 3)

Parathion and carbaryl were hydrolyzed by isolated perfused viable and anatomically intact pig skin yielding para-nitrophenol and naphthol, respectively (Chang et al. 1994). Alpha-tocopheryl acetate was metabolized in micro-Yucatan pig skin to the active antioxidant, alpha-tocopherol to the extent of 15–20 % of the amount of alpha-tocopheryl acetate permeated in the skin (Rangarajan and Zatz 2001). No hydrolysis was detected in the stratum corneum. Six 1-alkylazacycloalkan-2-one prodrug esters of ketoprofen were efficiently hydrolyzed by pig skin (Bonina et al. 2003).

Methyl-, ethyl-, butyl-, and benzylparabens, esters of 4-hydroxybenzoic acid, applied to the surface of minipig skin were hydrolyzed to 4-hydroxybenzoic acid. The effects of the carboxylesterase inhibitors paraoxon and bis-nitrophenylphosphate provided evidence of the involvement of dermal carboxylesterases. Parabens applied to the surface of minipig or human skin were absorbed to a similar amount and metabolized to 4-hydroxybenzoic acid during dermal absorption implying to the authors that the minipig is a suitable model for man for assessing dermal absorption and hydrolysis of parabens, although the carboxylesterase profile in skin differs between human and minipig (Jewell et al. 2007).

The hydrolysis of [Arg8]-vasopressin in the pig skin was inhibited by bestatin, suggesting that the major contributor to the enzymatic hydrolysis was aminopeptidase (Bi and Singh 2000).

Minipig skin microsomes hydrolyzed the prototypical carboxylesterases substrates naphthyl acetate and para-nitrophenyl acetate with efficiencies (=*V*_max_/*K*_m_) between 1.2 and 4.2 min^−1^ mg^−1^, which were very similar to those of human skin micrososmes (1.3–4.2 min^−1^ mg^−1^) while those of rat skin microsomes (580–1,100 min^−1^ mg^−1^) were quite different from those of human. Also, minipig skin cytosol hydrolyzed naphthyl acetate and para-nitrophenyl acetate with efficiencies (18–61 min^−1^ mg^−1^) more similar to those of human skin cytosol (2.4–67 min^−1^ mg^−1^) than those of rat skin microsomes (80–100 min^−1^ mg^−1^) (Prusakiewicz et al. 2006).

In the excised skin of Yucatan micropig, methyl paraben was efficiently hydrolyzed to 4-hydroxybenzoic acid. However, in the presence of ethanol, transesterification forming ethyl para-hydroxybenzoate occurred much more readily than the hydrolysis to para-hydroxybenzoic acid (Oh et al. 2002) demonstrating the strong influence of the formulation on xenobiotic metabolism.

### Conjugating enzymes

#### Glutathione *S*-transferase (GST)

GST activity for the broad-spectrum substrate CDNB was observed in pig skin. In a comparative study, Jewell et al. (2000) found that cutaneous GST activity toward CDNB was lowest in the mouse compared with higher activities in pig < rat < human (Table 4). The levels of the cofactor necessary for GST activity, GSH, in the pig skin were 18.6 ± 1.5 nmol GSH/cm^2^ (Jewell et al. 2000).

#### UDP-glucuronosyltransferase (UGT)

The pesticide Propoxur (2-isopropoxyphenyl *N*-methylcarbamate) applied in vitro to skin from pig, human, and rabbit yielded 2-isopropoxyphenol (IPP), followed by phase II conjugation. In pig skin, glucuronides and sulfates were formed in equal amounts (while in human skin, only sulfate conjugation was observed, and for rabbit skin, glucuronidation was the major route of conjugation with minor amounts of the sulfate conjugate generated) (van de Sandt et al. 1993). Dressler and Appelqvist (2006) tentatively showed that pig skin UGT metabolized acetylaminophenol (paracetamol) to the glucuronide.

Jacques et al. (2010a) demonstrated in short-term skin cultures from ears of domestic pigs the biotransformation of [^14^C]-7-ethoxycoumarin to the glucuronide of the primary deethylated metabolite 7-hydroxycoumarin. The glucuronide was the major metabolite, its formation amounting to 7.71 ± 0.73 pmol/h/mg protein (equivalent to 26.70 ± 2.54 pmol/h/cm^2^ skin and equivalent to 333.24 ± 31.75 pmol/h/g skin tissue). These numbers refer to the metabolite analyzed in the culture medium. Minor amounts of metabolites were also found in the cultured skin tissue (after 48 h, 70 % of the radioactivity was recovered in culture media, 10 % in the skin, including unchanged 7-ethoxycoumarin).

Likewise, Jacques et al. (2010b) observed that ^14^C-benz[*a*]pyrene (BP) was extensively metabolized by pig ear skin microsomes and similarly by pig ear skin explant short-term cultures, as most abundant metabolites to the glucuronides of the primary oxidative metabolites, the hydrox-BPs, and to a minor extent to the glucuronides of the BP-diols.

Also, bisphenol A was metabolized to its mono-glucuronide as the major metabolite formed by pig ear fresh explant culture (but not after freezing) (Zalko et al. 2011).

#### Sulfotransferase (SULT)

As discussed above under UDP-glucuronosyltransferase, the pesticide Propoxur (2-isopropoxyphenyl *N*-methylcarbamate) applied in vitro to skin from pig, human, and rabbit yielded 2-isopropoxyphenol (IPP), followed by phase II conjugation. In pig skin, sulfates and glucuronides were formed in equal amounts (while in human skin, only sulfate conjugation was observed, and for rabbit skin, glucuronidation was the major route of conjugation with minor amounts of the sulfate conjugate generated) (van de Sandt et al. 1993). Dressler and Appelqvist (2006) tentatively showed that pig skin metabolized acetylaminophenol to the sulfate.

Jacques et al. (2010a) demonstrated in short-term skin cultures from ears of domestic pigs the biotransformation of [^14^C]-7-ethoxycoumarin to the sulfate of the primary deethylated metabolite 7-hydroxycoumarin. The sulfate was the second most abundant metabolite, its formation amounting to 4.24 ± 0.66 pmol/h/mg protein (14.67 ± 2.29 pmol/h/cm^2^ and 183.04 ± 28.53 pmol/h/g tissue). These numbers refer to the metabolite analyzed in the culture medium. Minor amounts of metabolites were also found in the cultured skin tissue (after 48 h, 70 % of the radioactivity was recovered in culture media, 10 % in the skin, including unchanged 7-ethoxycoumarin).

Likewise, Jacques et al. (2010b) observed that ^14^C-benz[*a*]pyrene (BP) was extensively metabolized by pig ear skin microsomes and similarly by pig ear skin explant short-term cultures, as second most abundant metabolites to the sulfates of the primary oxidative metabolites, the hydrox-BPs.

Also, bisphenol A was metabolized to its mono-sulfate as the second major metabolite formed by pig ear fresh explant culture (but not after freezing) (Zalko et al. 2011).

#### *N*-Acetyltransferase (NAT)

One major pathway of the many topically applied amines is *N*-acetylation, which also occurs in the porcine skin. After dermal application, para-aminophenol is converted to para-acetylaminophenol (paracetamol), para-phenylenediamine to *N*,*N*′-diacetylated para-phenylenediamine. Both of these represent the sole metabolite observed in blood plasma, suggesting that topically applied amines are metabolized in the pig skin, presumably by NAT1, resulting in systemic exposure to acetylated metabolites, and not to their parent arylamines (Dressler and Appelqvist 2006).

## Xenobiotic-metabolizing enzymes in the human skin including skin-derived cells and cell lines as well as reconstructed skin models

The EU Cosmetic Directive prohibits the use of animal experiments for all safety assessments of cosmetic products and their ingredients from March 2013 onward (Council Directive 76/768/EEC, 1976). Human in vivo studies are an ethical concern and technically challenging. Cell cultures have restrictions in cell diversity, chemical solubility, and metabolic competence. Reconstructed skin mimicking organotypic stratified epidermis skin represents new alternatives (dos Santos et al. 2011; Jäckh et al. 2011, 2012). Investigations on XME in reconstructed human skin models will therefore be discussed here (usually in the end of each chapter), and an overview on currently commercially available reconstructed human skin models is presented in Table 8, while an overview on cell lines derived from human skin is given in Table 7.

**Table 7:** Overview of frequently used cell lines for studies on cutaneous xenobiotic-metabolizing enzymes

Cell line	Type	Reference and/or origin
HaCaT	Spontaneously immortalized aneuploid immortal, but non-tumorigenic human keratinocytic cell line	Boukamp et al. (1988)
NCTC 2544	Human breast skin keratinocyte-derived cell line	Bakken et al. (1961)
KeratinoSens^®^	Possess a luciferase gene under control of the human aldo keto reductase AKR1 C2 antioxidant response element (ARE)	Givaudan
LuSens	Derived from human keratinocytes; possess a luciferase gene under control of the rat NADH/NADPH quinone reductase NQO1 antioxidant response element (ARE)	Bauch et al. 2011; Ramirez et al. 2014/BASF SE
U937	Established from a diffuse histiocytic lymphoma displaying many monocytic characteristics	Galvão dos Santos et al. 2009/DSMZ, Braunschweig
THP-1	Derived from the blood of a patient with acute monocytic leukemia	Qin 2012/DSMZ, Braunschweig

*DSMZ* Deutsche Sammlung von Mikroorganis-men und Zellkulturen GmbH (German Collection of Microorganisms and CellCultures)

### Cytochromes P450 (CYP)

The presence of xenobiotic-metabolizing CYP mRNAs in the untreated, healthy human skin appears to be unequivocally demonstrated, but the presence of xenobiotic-metabolizing CYP protein and enzymatic activity is controversial. If present, it appears clear, that their levels are much lower than in the liver. Findings and failure of findings will be discussed in the following.

#### CYP transcript expression

##### Human skin

Transcripts coding for CYPs were found in human skin samples in several studies: in the study by Yengi et al. (2003), the major CYP transcripts detected in 10-mm punch biopsies of full-thickness skin from 18- to 45-year-old healthy males were CYP1B1, CYP2B6, CYP2D6, and CYP3A4 with mean values of 2.5, 2.6, 2.7, and 1.1 fg/18S rRNA in 50 ng total RNA, respectively. Lower levels of CYP1A1, CYP2C9, CYP2C18, CYP2C19, CYP2E1, and CYP3A5 transcripts were also detected while CYP1A2, 2A6, and 2C8 transcripts were below detection. There was interindividual variation in the levels of mRNA among the 27 subjects studied, although Poisson analysis showed data to be normally distributed, except for CYP2B6, as some individuals completely lacked CYP2B6 mRNA.

Baron et al. (2008) reported that array analysis at the transcript level showed mRNA expression above background for 36 CYPs in human whole-skin samples, most notably 1A1, 1A2, 1B1, 2A6/7, 2B6/7, 2C9, 2C18, 2C19, 2D6, 2E1, 2S1, 3A4/7, 3A5. Expression of CYP 1A1, 1B1, 4B1, 4X1, 19A1, and 26B1 transcripts was higher in skin samples compared to liver samples.

CYP1A1, CYP1A2, CYP1B1, CYP2C18 transcripts were induced in adult human full-thickness skin (4 mm) punch biopsy samples by coal tar, CYP26 and NADPH P450 reductase by all-*trans*-retinoic acid, and CYP3A5 by clobetasol 17-propionate while CYP1A1 and CYP1A2 transcript expression was suppressed by all-*trans*-retinoic acid (Smith et al. 2006).

Of the transcripts of which Janmohamed et al. (2001) demonstrated the presence in adult human skin (2A6, 2B6, 3A4), only the mRNA encoding CYP2B6 decreased with increasing age of the individual. In situ hybridization showed that the mRNA expression of each of the three CYPs analyzed was localized to the epidermis, sebaceous glands, and hair follicles (Janmohamed et al. 2001).

##### Cells in culture (for cell lines, see overview in Table 7)

Transcripts coding for CYPs in primary cultures and cell lines were also found in several studies. In the study by Janmohamed et al. (2001) of the mRNAs of the CYPs investigated (CYP2A6, 2B6, 3A4), all were substantially less abundant in cultures of keratinocytes than in samples of skin from which the cells were derived. HaCaT, an immortalized human keratinocyte cell line, expressed CYP2B6 mRNAs in similar amounts as in the whole-skin samples, but CYP2A6 and CYP3A4 mRNAs were not detected in HaCaT cells (Janmohamed et al. 2001). In a spontaneously immortalized human keratinocytic cell line (SIK), the mRNA of the following CYPs was expressed: CYP4B1, CYP11A1, CYP4F3, CYP2A7, CYP4F2, and CYP51 (Rea et al. 2002).

Vondracek et al. (2001, 2002) used standardized and quantitative, reverse transcription-polymerase chain reaction (StaRT-PCR) and microarray chip techniques to analyze transcript levels of CYPs in cultured normal human oral keratinocytes (NOK) and a Siman virus 40 T antigen-immortalized oral keratinocyte line SVpgC2a. With good agreement between the 2 methodologies, NOK and SVpgC2a were found to express similar levels of transcripts for CYP2B6/7, CYP 2E1, CYP oxidoreductase, and the aryl hydrocarbon receptor nuclear translocator. In contrast, SVpgC2a exhibited comparatively higher levels of CY1A1, 1B1, and aryl hydrocarbon receptor. Transcripts for CYP2A6/7 were not detected. Generally, the comparison of NOK from 2 individuals indicated relatively similar transcript levels of these enzymes. In contrast, differences between NOK and SVpgC2a, e.g., for CYP1B1, may reflect alteration caused by immortalization.

CYP mRNA coding for CYP1A1, 1B1, and 2E1 was found in all four cell types extracted from cultured Langerhans cells, keratinocytes, fibroblasts, and melanocytes (Saeki et al. 2002). In Langerhans cells, mRNA coding for CYP1A1, 1B1, 1E1, and 3A4 was observed in all 6 individuals, 3A7 in 3 of the 6 individuals; in keratinocytes, CYP1A1, 1B1, 2E1, and 3A5 in all individuals, CYP2C in 5 individuals, and 4B1 in 3 individuals; in fibroblasts, CYP1A1, 1B1, 2D6, 2E1, and 3A7 in all 6 individuals, CYP3A5 in 5, 2C in 4, and 2A6 in 2 individuals; and in melanocytes, CYP1A1, 1B1, 2A6, and 2E1 in all 6 individuals. In contrast to the further above-mentioned findings, quite a number of CYP mRNA (CYP1A2, 2A7, 2B6, and 3A4 mRNA) were not detectable in keratinocytes in this study (the expression pattern of CYPs was not changed upon maturation of the keratinocytes, but they were cultured for 36 h only [with 1.5 mM Ca^2+^]). However, in more recent studies (Du et al. 2006a, b) where it was ascertained by monitoring the morphology and the upregulation of keratin 10 and transglutaminase 1 mRNA that the first differentiation stage of keratinocytes (the spinous stage) was actually reached after 6 days in cultures, an impressive number of CYP mRNA was identified: CYP1A1, 1A2, 1B1, 2C9, 2C18, 2C19, 2D6, 2E1, 2J2, 2S1, 2U1, 2W1, 3A4, 4B1. Several of them were upregulated by cellular differentiation: CYP2C9, 2C18, 2C19, 2W1, 3A4, and 4B1. Expression of mRNA for these CYP genes in differentiating keratinocytes was lower after exposure to retinoic acid (a negative regulator of epidermal differentiation in vitro [Fisher and Voorhees 1996]) and also after exposure to aryl hydrocarbon receptor ligands. CYP2U1 was expressed at highest levels in undifferentiated keratinocytes. CYP2R transcripts had also been detected in human keratinocyte cultures differentiated for 6 days (Du et al. 2004).

In fetal human keratinocytes, the mRNA of the following CYPs was identified: 1A1, 1A2, 1B1, 2A6, 2B6, 2C8, 3A4, 26A1 (Swanson 2004). Some of them were induced upon treatment with dioxin (CYP1A1, CYP1A2, CYP1B1) (Swanson 2004).

##### Reconstructed skin models (overview in Table 8)

Neis et al. (2010) observed by real-time PCR analysis transcripts for CYP1A1, 1B1, 2E1, 2C18 (weak), 2J2, 3A4 (weak), 3A5, 4B1, but not 2B6 in all three commercially available organotypic skin models, the EpiDermFT™ full-thickness skin equivalent (MatTek, Ashland, Mass., USA), the Phenion^®^ full-thickness skin model (Phenion/Henkel, Düsseldorf, Germany), and the AST-2000 (“Advanced Skin Test”) full-thickness skin model (CellSystems^®^, St. Katharinen, Germany) as well as in an in-house model. The expression of CYP1A1 and 1B1 transcripts was induced by treatment with liquor carbonis detergens in all tested systems, highly induced in all of them except Phenion^®^ where CYP1A1 was only weakly induced. In the study by Luu-The et al. (2009), transcripts coding for the following CYPs involved in xenobiotic metabolism were found in total skin, human epidermis as well as in EpiSkin™ and EpiskinTMF™, at low levels (below approximately 200,000 copies per µg total RNA): CYP2B6, 2D6, 2E1, 1A1, 1B1, 2C8, 2C18, 2F1, and 3A5, while transcripts coding for CYP2C9, 1A2, and 3A7 were found at very low levels (below 10,000 copies per µg total RNA). Although in the study of Hu et al. (2010) on the expression of xenobiotic-metabolizing enzyme transcripts in human skin and the reconstructed human skin model EpiDerm™ the concordance between full-thickness human bottom skin (FTHBS) and EpiDerm™ tissues was high (83–86 % agreement), exceptions were the transcripts coding for CYP1A1, 2A6, 2E1, 4X1, and 8B1, which were detected in FTHBS, but not in EpiDerm™. Transcripts for CYP2D6, 2U1, and 2W1 were seen in FTHBS, but their presence was variable between EpiDerm™ samples. CYP1A2, 1B1, 2C9, 2C18, 2J2, 2R1, 2S1, 3A5, and 4B1 transcripts were present in FTHBS and EpiDerm™, CYP2B6, 2C8, 2C19, 2F1, 3A4, and 3A7 were neither detected in FTHBS nor in EpiDerm™ (further descriptions of CYP transcript expressions in human reconstructed skin models [and native skin]: Hayden et al. 2006; Rassmussen et al. 2011; Wiegand et al. 2008).

**Table 8:** Overview of commercially available reconstructed human skin models^a^

Skin model	Type	Company	Cell origin	Reference
EpiDerm™ (EPI-200)	Epidermal	MatTek, MA, USA	Male foreskin	Hayden et al. (2006)
EpiDermFT™ (EFT)	Full thickness (collagen matrix)	MatTek, MA, USA	Male foreskin	Hayden et al. (2003)
EST-1000 (EST)	Epidermal	Cell Systems, Troisdorf, Germany	Male foreskin	Hoffmann et al. (2005)
AST-2000 (AST)	Full thickness (collagen matrix)	Cell Systems, Troisdorf, Germany	Male foreskin	Hoffmann et al. (2003)
Episkin™	Epidermal	SkinEthic™ laboratories, Nice, France	Adult breast skin	Tinois et al. (1991) Netzlaff et al. (2005)
Episkin™ FTM	Full thickness (polycarbonate and collagen matrix)	SkinEthic™ laboratories,	Adult breast skin	Eilstein et al. (2010)
SkinEthik™ RHE	Epidermal	SkinEthic™ laboratories, Nice, France	Adult abdomen Male foreskin	Netzlaff et al. (2005) Eilstein et al. (2014)
Phenion^®^ FT (PFT)	Full thickness (collagen matrix)	Henkel, Düsseldorf, Germany	Male foreskin	Mewes et al. (2007)
StrataTest^®^	Epidermal	StrataTech, MA, USA	NIKS^®^ human keratinocyte cell line	Slavik et al. (2007)
EuroSkin^®^	Epidermal	EuroDerm, Leipzig, Germany	Hair follicles	http://www.eurodermbiotech.de (2011)
EPI-MODEL	Epidermal	LabCyte, Aichi, Japan	?	Katoh et al. (2009)

NIKS^®^, spontaneously immortalized near-diploid human keratinocyte cell line

^a^Adapted from: Jäckh et al. (2012)

##### Other aspects

Monocytes and dendritic cells play a critical role in antigen processing and presentation leading to several allergic reactions such as allergic dermatitis. Moreover, freshly isolated whole-skin samples are not free of residual blood. It may therefore be of some interest that in human peripheral blood monocytes and in dendritic cells (CD80+/CD83+/CD14−) obtained after incubation of blood monocytes with GM-CSF and IL-4 over 5 days, remarkably CYP1B1 showed the highest mRNA expression of all CYPs investigated. In monocytes CYP1B1, 2E1, and 2B6/7, 2D6 and 2E1 transcripts were observed. In monocytes treated with benzanthracene, lipopolysaccharide (LPS), or the phorbol ester TPA, the expression of CYP1A1 was enhanced. Dendritic cells of all 17 examined individuals expressed CYP 1B1, 2B6, 2D6, and 2E1. CYP3A5 was found in 85 % of the individuals, CYP1A1 in 60 %, CYP1A2 and 2C19 only in 18 %. CYP2A6 and 3A4/7 were not detected (Sieben et al. 1999).

#### CYP protein expression

Constitutive levels of CYP1A1/A2 and CYP2B1/B2 proteins were observed in human skin by immunocytochemistry and shown to be concentrated in the epidermis and sebaceous glands (Pendlington et al. 1994). Katiyar et al. (2000) also reported CYP1A1 protein to be present in human skin and to be primarily localized in the basal cell layer of the epidermis as shown by immunohistochemistry. In the same study, CYP1B1 was localized to the epidermal cells other than the basal cell layer (Katiyar et al. 2000). The authors speculated that the different localizations of CYP1A1 as opposed to CYP1B1 may be related to keratinocyte differentiation. UVB exposure to previously solar-UV-protected skin (buttock) resulted in an induction of CYP1A1 and CYP1B1 in the epidermis. Reverse transcription-polymerase chain reaction and Western blot analyses showed that exposure to UVB (4 minimal erythema doses) resulted in enhanced expression of mRNA and protein of CYP1A1 and CYP1B1 in the epidermis.

Using immunofluorescence, Baron et al. (2001) identified CYP 1A1, 2B6, 2E1, and 3A protein in human foreskin sections. The CYP proteins were restricted to the cytoplasm and were preferentially localized to the suprabasal layer of the keratinocytes.

After treatment of human keratinocytes in culture in late subconfluency with “Target Unmasking Fluid,” Baron et al. (2001) showed by intracellular staining with specific antibodies using the “APAAP method” the presence of CYP1A1 protein in a few cells. After induction with benz[*a*]anthracene, the level of CYP1A1 and the number of cells harboring CYP1A1 were highly increased (Baron et al. 2001). Immunoblots using antibodies against individual CYP proteins showed in microsomal preparations from untreated proliferating human foreskin keratinocytes reactivity with CYP 1A1, 2B6, 2E1, and 3A.

A recent study (van Eijl et al. 2012) investigated the presence of XME in human whole skin (epidermis and dermis) by proteomics using custom-built PROTSIFT software. Protein identification was based on the presence of at least 2 different tryptic peptides in at least two donors. Samples of human whole skin were obtained from 10 healthy females (undergoing reduction mammoplasty; sedated with propofol and/or remifentanil, not receiving regular medication; mean age 44 ± 13 years). Thirteen CYP proteins encompassing all of the major forms involved in xenobiotic metabolism were not detected in human skin at the limit of detection (LOD) of 0.1–0.2 pmol/mg microsomal protein. The authors conclude that if these CYP proteins were present in the human skin, they would have to be at least 300-fold lower than in the liver. CYP1A2, CYP2E1, and CYP3A4 were also not detected in skin under immunoblotting conditions (LOD 2.5 pmol/mg microsomal protein) that readily showed their presence in human liver and CYP1A1 could not be detected in either skin or liver, although a preparation containing recombinant CYP1A1 was readily detected (van Eijl et al. 2012). In the reconstructed 3D human skin models, EpiDerm, EpiSkin™, and SkinEthik™ RHE CYP1, CYP2B6, and CYP3A proteins were also not detected (Hewitt et al. 2013).

Whether the further above-discussed studies claiming the presence of xenobiotic-metabolizing CYP proteins in reality were observing non-CYP proteins or whether the more recent studies presented just above did not detect CYP proteins which in reality were present in the human skin future studies may show.

#### CYP catalytic activities (see also Tables 1, 9, 10)

##### Aryl hydrocarbon hydroxylase (AHH)

Already, early investigations had shown that human skin possesses an enzymatic activity metabolizing polycyclic aromatic hydrocarbon carcinogens and to be inducible by them (Levin et al. 1972; Alvares et al. 1973a; Bickers et al. 1984). Most frequently, this activity was determined by measuring the formation of hydroxylated metabolites from the aromatic hydrocarbon (“aryl hydrocarbon”) benzo[a]pyrene (BP), and this activity is frequently termed “aryl hydrocarbon hydroxylase” (“AHH”). Levin et al. (1972) demonstrated low, but measurable AHH activity (2–4 pmol 3-OH-BP/mg protein in 30 min, which was 2–3 times that of the blank) in untreated human neonatal foreskin kept in culture for 16 h. This activity was increased 2- to 5-fold by treatment with benz[*a*]anthracene. Alvares et al. (1973a, b) showed that this activity had an absolute requirement for both, NADPH and molecular oxygen, had a pH optimum of 7.4, and was completely inhibited by carbon monoxide, indicating that this activity was completely dependent on CYP (at least in the investigated samples that were from neonatal human foreskin maintained for 16–20 h in culture). Merk et al. (1987a) also showed that AHH activity—in this case measured in freshly plucked human hair follicles—absolutely required NADPH and was inhibited by carbon monoxide more than 90 % (and also largely by other prototypical CYP inhibitors, e.g., in vitro 73 % inhibition by 10^−4^ M α-naphthoflavone; in vivo 200 mg ketoconazole daily for 5 days orally applied to healthy volunteers resulted in greater than 90 % inhibition of hair follicle AHH activity). Bickers et al. (1984) showed that human skin (S9 fraction of homogenate obtained from epidermal strips freshly excised from healthy, untreated human skin) had clearly measurable AHH activities: 62.1 (± 5.6 SE) fmol 3-OH-BP equivalents/min/mg protein.

**Table 9:** Representative cytochrome P450 (CYP) activities^a^ in human skin and human cells in culture^b^

Activity (preferential for)	Skin microsomes	Keratinocytes^c^	HaCaT	NCTC 2544	KeratinoSens^®^	LuSens	U937	THP-1
AHH (CYP1 family)	0.24–1.35^d^							
EROD (CYP1 family)	bd–35^d^	bd–10.7^d^/19^d^	bd–0.047^d^–21.9^e^/253 ± 35^e^	0.2–0.3^d^; 2 ± 0.1^f^/114 ± 77^d^	bd	bd	bd	bd
MROD (CYP1A2)	bd to +		?/35^d^	?/70^d^				
PROD (CYP2B6)	bd to bq	bd–1.43^d^/2.7^d^	bd/bd	bd–0.6 ± 0.05^f^	bd	bd	bd	bd
MFCOD (CYP1/2, especially 2C9)	bd							
BROD (CYP2B/3A)					bd	bd	bd	bd
Tolbutamide 4-hydroxylation (CYP2C9)	0.46 ± 0.05^e^		bd					
Bufuralol 1-hydroxylation (CYP2D6)	bd		bd					
Chlorzoxazone 6-Hydroxylation (CYP2E1)	2.83 ± 0.34^e^		bd					
Para-nitrophenol hydroxylation (CYP2E1)	bd/+	1810^d^						
Midazolam 1-hydroxylation (CYP3A4)	2.35 ± 0.23^e^		bd					
BQOD (CYP3A)	bd–76 ± 41^d^	bd/bd	38 ± 13^d^	33 ± 5^d^				
Erythromycin *N*-demethylase (CYP3A) +	3260^d^							

More examples and references in the text

*AHH* aryl hydrocarbon hydroxylase, phenolic benzo[*a*]pyrene metabolites determined with 3-hydroxy-benzo[*a*]pyrene as standard, *bd* below detection, *BROD* 7-benzyloxyresorufin *O*-debenzylase, *BQOD* benzyloxyquinoline *O*-dealkylase, *ECOD* 7-ethoxycoumarin *O*-dealkylase, *EROD*, 7-ethoxyresorufin *O*-dealkylase; MFCOD, 7-methoxy-4-trifluoromethylcoumarin *O*-dealkylase; MROD, 7-methoxyresorufin *O*-deethylase; PROD, 7-pentoxyresorufin *O*-dealkylase

^a^Constitutive; number after slash: induced (highest reported induced activity)

^b^For description of the cells in culture, see Table 7

^c^Primary keratinocytes in culture (“NHEC,” “normal human epithelial keratinocytes”)

^d^pmol/min/mg microsomal protein

^e^pmol/h/mg microsomal protein

^f^pmol/min/mg protein determined in culture medium

**Table 10:** Representative cytochrome P450 (CYP) activities^a^ in human skin and human reconstructed skin models^b^

Activity (preferential for)	Skin	EpiDerm™	Episkin™	Episkin™ FTM	SkinEthik™ RHE	Phenion^®^ FT	StrataTest^®^
AHH (CYP1 family)	0.24–1.35^d^						
EROD (CYP1 family)	bd-35^d^ 3.0 ± 1.2^g^	bd/1.7 ± 0.8^f^	7.8 ± 0.4^g^	2.6 ± 0.3^g^	2.8 ± 0.9^g^	bd	<1^d^
MROD (CYP1A2)	bd to +	bd/0.7 ± 0.3^f^					
PROD (CYP2B6)	bd to bq	bd/bd				bd	<4^d^
MFCOD (CYP1/2, especially 2C9)	bd ~≤0.5^g^	bd/bd	~≤0.5^g^	~≤0.5^g^	~≤0.5^g^		
BROD (CYP2B/3A)		bd				bd	<1^d^
Tolbutamide 4-hydroxylation (CYP2C9)	0.46 ± 0.05^e^						
Bufuralol	bd						
1-Hydroxylation (CYP2D6)							
Chlorzoxazone 6-hydroxylation (CYP2E1)	2.83 ± 0.34^e^						
Para-nitrophenol hydroxylation (CYP2E1)	bd/+						
Midazolam 1-hydroxylation (CYP3A4)	2.35 ± 0.23^e^						
BQOD (CYP3A)	bd-76 ± 41^d^	94 ± 13^d^/5.5 ± 0.9^f^					
Erythromycin *N*-demethylase (CYP3A)	+						

More examples and references in the text

*AHH* aryl hydrocarbon hydroxylase, phenolic benzo[*a*]pyrene metabolites determined with 3-hydroxy-benzo[*a*]pyrene as standard, *bd* below detection, *BROD* 7-benzyloxyresorufin *O*-debenzylase, *BQOD* benzyloxyquinoline *O*-dealkylase, *ECOD* 7-ethoxycoumarin *O*-dealkylase, *EROD* 7-ethoxyresorufin *O*-dealkylase, *MFCOD* 7-methoxy-4-trifluoromethylcoumarin *O*-dealkylase, *MROD* 7-methoxyresorufin *O*-deethylase, *PROD* 7-pentoxyresorufin *O*-dealkylase

^a^Constitutive; number after slash: induced (highest reported induced activity)

^b^For description of the reconstructed human skin models, see Table 1

^c^Primary keratinocytes in culture (“NHEC,” “normal human epithelial keratinocytes”)

^d^pmol/min/mg microsomal protein

^e^pmol/h/mg microsomal protein

^f^pmol/min/mg intact model protein

^g^pmol of products/6 h/mg protein (sum of 7,500*g* supernatant + medium)

AHH activities were higher in hair follicles than in total skin (Merk et al. 1987a). AHH activity was lower and less inducible in psoriatic lesions (Shuster et al. 1980) but not significantly different in unaffected skin in the surrounding tissue of psoriatic patients compared with healthy subjects (Bickers et al. 1984) (while in psoriatic lesions, the expression of CYP2E1 and CYP2S1 mRNA was increased [Smith et al. 2003a; Merk and Baron 2004]).

Such as in other species and in other tissues, human skin AHH can convert aromatic hydrocarbons to reactive metabolites, which covalently bind to DNA, RNA, and proteins. Kang-Sickel et al. (2010) observed naphthyl-keratin adducts in dermal tapestrip samples collected from naphthalene-exposed workers (0.004–6.104 pmol adduct/μg keratin), indicating the potential use of keratin adducts as biomarkers of dermal exposure.

Brinkmann et al. (2013) demonstrated in primary human keratinocyte cultures (“normal human epithelial keratinocytes”; NHEK) exposed to 3.5 µM BP BP-derived metabolites, including *trans*-BP-7,8-diol and BP-7,8,9,10-tetraol in readily quantifiable amounts. The presence of BP-7,8,9,10-tetraol indicates that the ultimate carcinogen (+)-anti-BP-7,8-diol-9,10-epoxide (BPDE) had been transiently generated in NHEK cultures. Cultures of primary human skin-derived fibroblasts generated amounts of metabolites comparable to those formed by NHEK, except that fibroblast cultures produced lower levels of BP-7,8,9,10-tetraol (0.9 ± 1.5 pmol per mg wet weight vs. 8.2 ± 2.9 pmol per mg wet weight after 72 h of incubation) and higher levels of *trans*-BP-7,8-diol (22.7 ± 11.3 pmol per mg wet weight vs. 3.9 ± 1.5 pmol per mg wet weight after 72 h of incubation) than NHEK. Compared with NHEK, BP metabolism in fibroblasts can thus be expected to generate lower overall amounts of the ultimate carcinogen BPDE. Comparison of the metabolic profiles of excised human skin ex vivo with NHEK and primary human fibroblasts in culture and with commercially available epidermal (MatTek EpiDerm) and full-thickness (MatTek EpiDermFT) skin models showed that these models convert BP into its three different main classes of metabolites and indicate a good accordance in metabolic capacity between human skin and these models. Compared with human skin ex vivo and the skin models, confluent NHEK generated 32.5-fold less metabolites (2,450 pmol per 60.1 cm^2^ culture dish vs. 1,335 pmol per cm^2^ in skin) over 48 h when normalized to model areas.

##### Prototypical CYP catalytic activities other than AHH

*Human skin* Damen and Mier (1982) found no 7-ethoxycoumarin *O*-dealkylase (ECOD) (prototypical CYP1A and 2B substrate) activity (<3 fmol/min/mg tissue) in human skin (0.2 mm thick, encompassing the whole epidermis, but only part of the dermal adnexae) homogenate (while in rat and mouse skin homogenates, there were clear-cut activities of 12 ± 8 and 48 ± 23 fmol/min/mg tissue, respectively).

However, Merk et al. (1987b) found ECOD activity in freshly plucked human hair follicles. Topical application of PAHs (in liquor carbonis detergens) to the scalp of human volunteers enhanced the activity. Oral and topical administration of ketoconazole resulted in an up to 73 % inhibition of the activity. Ademola et al. (1993a) observed ECOD activity in human skin of 29- to 55-year-old individuals.

In a comparative study, Rolsted et al. (2008) found in human skin microsomes the following activities: EROD below detection (LOQ [limit of quantification] 1.87 pmol), PROD below detection (LOQ 1.87 pmol), tolbutamide 4-hydroxylation (prototypical for CYP2C9) 0.46 ± 0.05 pmol/h/mg protein, bufuralol 1-hydroxylation (prototypical for CYP 2D6) below detection (LOQ 1.08 pmol), chlorzoxazone 6-hydroxylation (prototypical for CYP2E1) 2.83 ± 0.34 pmol/h/mg protein, midazolam 1-hydroxylation (prototypical for CYP3A) 2.35 ± 0.23 pmol/h/mg protein.

Using the CYP3A substrate 7-benzyloxyquinoline (BQ) and the luminescent Luc-BE CYP3A assay, very low (just exceeding the LOD) activities of 76 ± 41 pmol BQ dealkylated product/min/mg and 0.05 ± 0.03 pmol Luc-BE/min/mg protein were detected in human skin microsomes, while no EROD, 7-methoxyresorufin *O*-demethylase (MROD), PROD, or *O*-dealkylase activities for 7-methoxy-4-trifluoromethylcoumarin [multi-CYP1⁄2 substrate with relatively high activity for CYP2C (Szotáková et al. 2004)] above the LOD were seen (LOD for EROD, MROD and PROD < 0.2 pmol⁄min⁄mg protein) (Götz et al. 2012a). Jäckh et al. (2011) and Rolsted et al. (2008) also did not detect EROD activity above the LOQ (2 pmol/min/mg protein and 1.87 pmol, respectively) in human skin microsomes.

In fresh human full-thickness skin (dermis + epidermis, no subcutaneous tissue) punch biopsies (6–8 mm), Du et al. (2009) observed leukotriene B4 (LTB4) hydroxylation activity converting LTB4 to 20-OH-LTB4. This metabolic reaction represents an inactivation of LTB4, which is an innate mechanism to resolve tissue inflammation. Retinoic acid exposure induced microsomal LTB4 hydroxylation activity in human skin cells. The authors conclude from their data that 20-hydroxylation is the major CYP-dependent LTB4 inactivation pathway in human skin and that this retinoid-inducible metabolic pathway has capacity to modulate tissue levels of proinflammatory lipids.

*Cell and organ culture* (for cell lines, see overview in Table 7). Raffali et al. (1994) reported on ECOD activity in intact cultured human keratinocytes, which was induced by low concentrations of the imidazole derivatives miconazole and econazole and inhibited at high concentrations, while the imidazole derivatives clotrimazole and sulconazole only had an inducing effect on ECOD activity (imidazole itself had no apparent effect on ECOD activity).

Hirel et al. (1995) observed in adult human keratinocytes cultured in submerged conditions EROD and phenacetin deethylase activities. After confluence, phenacetin deethylase showed a slight decrease, whereas EROD activities were decreased by 65 %. No major differences were observed between keratinocytes in primary culture and those in second subculture. Maximum EROD activity was obtained with 1 µM 3-MC and 20 µM benzanthracene, in both pre-confluent and post-confluent cultures. After freezing, EROD and phenacetin deethylase activities were only slightly reduced, if at all. In contrast to these results obtained in cultured human keratinocytes, Kao et al. (1985) had observed a drastic decrease in the metabolism of testosterone and benzo[a]pyrene after freezing of whole-skin samples that had been kept in short-term organ culture.

Hirel et al. (1996) observed in viable human keratinocytes obtained 24–50 h after death EROD and phenacetin deethylase activities (0.5–0.7 pmol/min/mg protein), which were similar as in keratinocytes from freshly obtained surgical samples.

The microsomal fractions of human keratinocytes in culture in late subconfluency showed the following enzymatic activities: 7-ethoxyresorufin *O*-deethylase (EROD, prototypic for the CYP 1 family, 10.7 pmol/min/mg protein), 7-pentoxyresorufin *O*-depentylase (PROD, prototypic for CYP2B, 1.43 pmol/min/mg protein), para-nitrophenol hydroxylase (PNPH, prototypic for CYP2E1, 1.81 nmol/min/mg protein), and erythromycin *N*-demethylase (EMND, prototypic for CYP 3A, 3.26 nmol/min/mg protein). EROD was enhanced 20-fold by pretreatment with benz[a]anthracene (Baron et al. 2001).

Delescluse et al. (1997) investigated the suitability of the keratinocyte-derived cell line HaCaT (Boukamp et al. 1988) as a substitute for primary cultures. EROD activity was stimulated by 3-MC in a concentration-dependent manner (maximal induction, 17-fold over control, at 1–2.5 nM 3-MC). Above this peak, the induction effect fell rapidly. Northern blot analysis of CYP1A1 mRNA generally agreed with the trends obtained for EROD values, but the decrease in the EROD activity at the highest 3-MC concentrations was not correlated with CYP1A1 mRNA reduction. The authors concluded that, in contrast to SV40-immortalized keratinocytes, the spontaneously immortalized keratinocyte cell line HaCaT may constitute a valuable tool for studying epidermal CYP1A1 gene regulation by xenobiotics.

Rolsted et al. (2008) reported in their comparative study in HaCat cells EROD activity of 21.9 ± 1.9 pmol metabolites/h/mg protein after induction with 5 µM β-NF 253 ± 35. All other diagnostic activities investigated were “not detected”: PROD (LOQ 1.87 pmol), tolbutamide 4-hydroxylation (prototypical for CYP2C9) (LOQ 1.08 pmol), bufuralol 1-hydroxylation (prototypical for CYP 2D6) (LOQ 4.07 pmol), chlorzoxazone 6-hydroxylation (prototypical for CYP2E1) (LOQ 12.8 pmol), midazolam 1-hydroxylation (prototypical for CYP3A) (LOQ 1.46 pmol). They also investigated the cell line KERT, but concluded their use is not recommendable for the evaluation of dermal CYP activities.

Bonifas et al. (2010) investigated EROD activities of three different HaCaT shipments and human primary keratinocytes (NHEK). Solvent-treated HaCaT showed EROD levels near the detection limit (0.047 pmol/mg/min), NHEK (*n* = 4) were in a range between 0 and 0.76 pmol/mg protein/min. B[a]P (1 µM) highly induced EROD activities to 19.0 ± 0.9 pmol/mg protein/min (*n* = 11) in HaCaT and 5.8 ± 0.5 pmol/mg protein/min (*n* = 4) in NHEK.

Gelardi et al. (2001) reported that in the human breast skin keratinocyte-derived cell line NCTC 2544 (Bakken et al. 1961) the prototypic CYP activities ECOD, EROD, and PROD were easily detectable in basal conditions and were susceptible to the classical CYP inducers β-naphthoflavone, 3-MC and phenobarbital, and to the classical CYP inhibitors α-naphthoflavone and metyrapone.

Götz et al. (2012a) investigated the activity and inducibility for prototypical CYP substrates in three human skin-derived cell types, the two keratinocyte-derived cell lines, HaCaT and NCTC 2544, and primary normal human epidermal keratinocytes (NHEKs). Basal EROD activity of HaCaT was below detection, and for NCTC and NHEK cells just above the LOD at 0.2–0.3 pmol/min/mg protein. EROD and MROD (MROD; prototypic for CYP1A2) activities were increased by 3-MC in all three cell lines, the highest EROD and MROD induction was in NCTC 2544 at 5 µM 3-MC (114 ± 77 and 70 pmol/min/mg protein, respectively). At that concentration, HaCaT cells showed 75 pmol/min/mg protein for EROD and 35 pmol/min/mg protein for MROD. NHEKs displayed maximal EROD activity using 2.5 µM 3-MC (11.1 ± 6.6 pmol/min/mg protein). Thus, immortalized cell lines like NCTC 2544 and HaCaT were more responsive to CYP induction by 3-MC than primary keratinocytes. On the other hand, CYP2B activity (probed with PROD as substrate) was not increased by treatment with rifampicin (50 µM), 6-(4-chlorophenyl)imidazo-[2,1-b][1,3]thiazole-5-carbaldehyde-*O*-(3,4-dichlorobenzyl)oxime (CITCO; 10 µM) or cyclophosphamide (500 µM) in any of the three investigated cell lines (Götz et al. 2012a).

In two keratinocytic cell lines, KeratinoSens^®^ (developed by Givaudan) and LuSens (developed by BASF SE), and in two dendritic cell lines, U937 und THP-1 (German Collection of Microorganisms and CellCultures), EROD, PROD, and BROD activities were below the LOD (Fabian et al. 2013).

*Reconstructed skin models.* Pham et al. (1990) already observed in a reconstituted epidermis model from the outer root sheath cells of human hair follicles EROD, PROD, and 7-benzoxyresorufin *O*-debenzylase activities. Cotovio et al. (1996) observed in reconstructed human epidermis EpiSkin™ (obtained from Imidex, Chaponost, France) ECOD activity, which was modulated by the dermatologically used imidazole derivatives econazole and clotrimazole with a biphasic effect: induction at low concentrations and inhibition at high concentrations. Dermatological preparations containing imidazole derivatives at non-toxic doses decreased ECOD activity by about 40 %. In reconstructed epidermal models of human skin in the early 2000s, EROD, although not detected without treatment, was found to be induced by 10 µM 3-MC in 5 of 6 batches of EpiDerm™, in 2 of 5 batches EpiSkin™, and in 2 of 6 batches SkinEthic™. ECOD was constitutively active and induced in the same batches in which EROD was induced. Clotrimazole inhibited the induced EROD and ECOD activities (Harris et al. 2002a). In EpiDerm™, after a 24-h treatment with 5 μM 3-MC, there was a marked elevation of EROD activity (Hayden et al. 2006). In the more recent study by Hu et al. (2010), low basal EROD activities were seen in EpiDerm™ and up to sixfold enhancement of activity upon treatment with 10 µM 3-MC. Jäckh et al. (2011) reported that CYP enzyme activities in the human skin models EpiDerm™ and Phenion^®^FT (PFT) remained below the LOD of 0.001 nmol/min/mg protein for EROD, 0.0025 nmol/min/mg protein for PROD, and 0.002 nmol/min/mg protein for BROD. Götz et al. (2012a) observed in EpiDerm™ microsomes very low, but quantifiable CYP3A activities (94 ± 13 pmol BQ⁄min⁄mg protein and 0.08 ± 0.05 pmol Luc-BE⁄min⁄mg protein), but no EROD, MROD, PROD, or 7-methoxy-4-trifluoromethylcoumarin *O*-dealkylase activities above the LOD were seen(LOD for EROD, MROD, and PROD < 0.2 pmol⁄min⁄mg protein). These results were similar to the findings in human skin microsomes obtained by the same authors (discussed above under “*Human skin”*). In intact EpiDerm™, tissue activities were induced by 3-MC up to 1.7 ± 0.8 pmol/min/mg protein for EROD and 0.7 ± 0.3 pmol/min/mg protein for MROD. PROD and 7-methoxy-4-trifluoromethylcoumarin *O*-dealkylase activities remained below the LOD after treatment with rifampicin- or CITCO (6-(4-chlorophenyl)imidazo[2,1-b][1,3]thiazole-5-carbaldehyde-*O*-(3,4-dichlorobenzyl)oxime). CYP3A4 activity was not induced by rifampicin or dexamethasone (Götz et al. 2012a). In the human epidermal skin model StratTest^®^, EROD, PROD, and BROD activities were below the LOD of 1, 4, and 1 pmol/min/mg protein, respectively (Guth 2013). Most recently, Eilstein et al. (2014) reported the following comparative *O*-dealkylase activities (expressed as pmol/mg protein/6 h; sum of S9 + medium): EROD in SkinEthik™ RHE (2.8) and in Episkin™ FTM (2.6) very similar to human skin (3.0), but higher in Episkin™ (9.1); 7-benzoxy 4-trifluoromethyl-coumarin *O*-deethylase activities (indicative for CYP3A5/3A7) in Episkin™ FTM (3.3) and Episkin™ (3.6) similar to human skin (3.8), but higher activity in SkinEthik™ RHE (13.3). The dealkylase activity for 7-methoxy-4-trifluoromethylcoumarin was close to the LOQ (0.5) in the three skin models investigated and in human skin (Eilstein et al. 2014).

#### Individual CYPs

Some CYPs which appear of special toxicological and/or pharmacological importance in the human skin will be discussed in more detail here below.

##### CYP1 family

Many polycyclic aromatic hydrocarbons such as BP and many planar polyhalogenated dibenzo-para-dioxins, polyhalogenated dibenzofurans, and coplanar polyhalogenated byphenyls highly induce in the skin and/or hair follicles of rodents and humans CYPs of the family 1 and/or enzymatic activities dependent on CYPs of the family 1 (Levin et al. 1972; Alvares et al. 1973a, b; Mukhtar and Bickers 1983; Finnen et al. 1984; Merk et al. 1987a; Whitlock 1987; Vecchini et al. 1995). The treatment with these inducers resulted in manyfold increases in CYP1A1 mRNA and protein as well as AHH activity (albeit not exclusively catalyzed by CYP1A1) in human keratinocytes. An important regulator of the induction of CYPs of the CYP1A family is the Ah receptor (AhR). In the human epidermal keratinocytes, the highest amount of AhR protein is found in the granular layer and spinous layer, and the lowest amount in the basal layer (Swanson 2004). Exposure of HaCaT (human keratinocyte cell line) to the AhR ligand FICZ (6-formylindolo[3,2-b]carbazole) even in the lower picomolar range caused significant CYP1A1 mRNA induction (Fritsche et al. 2007). Investigation of culture conditions allowing for CYP1A1 induction by 2,3,7,8-tetrachlorodibenzofuran (TCDF) in human keratinocytes showed that the keratinocytes grown in serum-free low extracellular Ca^2+^ (0.1 mM) medium did not accumulate CYP1A1 mRNA in response to TCDF. If the cultures were pretreated with serum or elevated extracellular Ca^2+^ (2 mM), induction of CYP1A1 was obtained by TCDF with an EC50 of approximately 2 nM. Culture conditions allowing for CYP1A1 induction correlated with conditions that induced mRNA for the differentiation-specific epidermal transglutaminase, suggesting that terminal differentiation is necessary for CYP1A1 induction in human keratinocytes by AhR ligands (Berghard et al. 1990). In HaCaT cells, AhR activation, binding to CYP1 target gene promoters, and CYP1A1 mRNA (and protein) expression induced by the polychlorinated biphenyl congener 3,3′,4,4′,5-pentachlorobiphenyl (PCB 126) were inhibited by hypoxia (exposure to 1 % O_2_ prior to PCB 126 treatment) (Vorrink et al. 2014). Some compounds, such as carbaryl, induced CYP1A1 expression in HaCaT without being a ligand for (human) AhR (Ledirac et al. 1997). Storm et al. (1990) did not detect BP metabolism in uninduced, intact, metabolically viable skin of humans, while in Sencar mice and hairless guinea pigs, BP metabolism was clearly detectable, albeit low. However, AHH activity and activity toward prototypical substrates of family 1 CYPs have been reported in untreated, healthy human skin in several (albeit not all) studies as discussed just above under “CYP catalytic activities.”

*CYP1A1 protein* is localized in the epidermis. Katiyar et al. (2000) reported that in contrast to most other CYPs and to most other xenobiotic-metabolizing enzymes, CYP1A1 protein (in buttock skin from 29- to 55-year-old non-smokers) is localized mostly in the basal cells. However, Baron et al. (2001) reported that CYP 1A1 protein (in human foreskin sections) was preferentially localized to the suprabasal layer of the keratinocytes, similar to the localization of other CYPs such as CYP2B6, 2E1, and 3A protein. Polycyclic aromatic hydrocarbons, such as BP, dibenzo[a,l]pyrene, or 7,12-dimethylbenz[a]anthracene, are excellent substrates for CYP1A1. CYP1A1 catalyzes the formation of genotoxic carcinogens such as epoxides, but at the same time also of non-genotoxic products such as phenolic metabolites (which may then be further metabolized to more easily excretable conjugates). In line with this, enhancement (or also decrease) of CYP1A1 activities has opposite effects depending of the route of administration (leading to first-pass effect or to immediate target tissue exposure) (Nebert et al. 2004). Metabolic toxication of dibenzo[a,l]pyrene, the most carcinogenic of all polycyclic aromatic hydrocarbons, is very efficiently catalyzed by human (heterologously expressed) CYP1A1, better than by other human CYPs and much better than by any rat CYP (Schober et al. 2006).

*CYP1A2* Ademola et al. (1993a) observed that human skin was able to *N*-demethylate theophylline, an activity ascribed to CYP1A2. Also, CYP1A2-typical MROD activity was observed in human skin. Metabolic toxication of dibenzo[a,l]pyrene, the most carcinogenic of all polycyclic aromatic hydrocarbons, is catalyzed by human (heterologously expressed) CYP1A2, much less efficient than by human CYP1A1 and 1B1, but better than by other human CYPs (Schober et al. 2006). Other CYP1A2 substrates (found in skin or other systems) include aromatic amines, acetanilide, polycyclic aromatic hydrocarbons, aflatoxin B1, phenacetin, acetaminophen, caffeine, theophylline, warfarin, imipramine (also 7-ethoxyresorufin like CYP1A1) (Du et al. 2004; Oesch-Bartlomowicz and Oesch 2007).

*CYP1B1* also plays a major role in the activation of polycyclic aromatic hydrocarbons to ultimate genotoxins. Metabolic toxication of dibenzo[a,l]pyrene is very efficiently catalyzed by human (heterologously expressed) CYP1B1, less efficient than by human CYP1A1 but much better than by other human CYPs and much better than by any rat CYP (Schober et al. 2006). This CYP has been shown to be expressed and to be highly inducible (mRNA 100-fold by treatment with nanomolar [10 nM] concentrations of 2,3,7,8-tetrachlorodibenzo-*p*-dioxin (TCDD) in primary human keratinocytes (Sutter et al. 1994). Induction of CYP1B1 expression by TCDD was also observed in human dermal fibroblasts (Akintobi et al. 2007). Also, UV-B exposure (20 mJ/cm^2^) increased human CYP1B1 transcripts in primary keratinocytes and HaCaT cell cultures, dependent on AhR. Among 20 human samples, large interindividual variability of CYP1B1 mRNA induction (1.1- to 4.5-fold) was observed (Villard et al. 2002). Pretreatment with an antioxidant, *N*-acetylcysteine, repressed CYP1B1 increase, suggesting the involvement of UV-B photoproducts. Cell-type-specific induction of CYP1B1 has been observed in that the prohapten carvoxime strongly induced CYP 1B1 in dendritic cells without having any effect on the CYP 1B1 expression in keratinocytes (Merk 2009). On the other hand, Murray et al. (1997) using immunostaining did not detect CYP1B1 protein in normal human skin (while in human skin cancer, there was strong immunoreactivity to anti-CYP1B1 antibodies of high specificity, which did not cross-react with CYP1A1 or CYP1A2). However, Katiyar et al. (2000) reported that they found CYP1B1 protein to be present in normal untreated human skin and to be localized in epidermal cells other than the basal layer. Polycyclic aromatic hydrocarbons and their dihydrodiols are important substrates of CYP1B1 (Oesch-Bartlomowicz and Oesch 2007).

##### CYP2 family

At least 13 CYP2 mRNAs (CYP2A6, 2A7, 2B6, 2C9, 2C18, 2C19, 2D6, 2E1, 2J2, 2R1, 2S1, 2U1, 2W1) are expressed in skin from at least some human individuals (Du et al. 2004).

*CYP2A subfamily* Of the 3 complete CYP2A genes in humans (CYP2A6, 2A7, and 2A13), only CYP2A6 and CYP2A7 are expressed in human skin. CYP2A7 transcripts have only been found in human skin fibroblasts, but no catalytic activity was observed upon heterologous expression (Ding et al. 1995). *CYP2A6* transcripts were detected in five of nine human skin samples, but not in proliferating keratinocyte cultures or in the keratinocyte-derived HaCaT cell line (Janmohamed et al. 2001), which suggests that CYP2A6 transcripts are produced in the dermis and/or in the terminally differentiated keratinocytes that were not represented in the proliferating cultures. 2A6 substrates (found in skin or other systems) include physiological compounds (all-*trans*-retinoic acid, arachidonic acid, progesterone) as well as xenobiotics (aflatoxin B_1_, coumarin, diallyl disulfide, nicotine, psoralene, 6-aminochrysene, several nitrosamines) (Oesch-Bartlomowicz and Oesch 2007).

*CYP2B subfamily.* Only one CYP2 gene, *CYP2B6*, is known to produce active protein in humans (Du et al. 2004). CYP2B6 transcripts were detected in all three of the human skin samples analyzed by Janmohamed et al. (2001) and in proliferating cultures of human keratinocytes and HaCaT cells (Baron et al. 2001). Hu et al. (2010) detected CYP2B6 transcription in native human skin. However, in full-thickness (epidermis and dermis) skin punch biopsies from 27 humans, CYP2B6 transcripts levels ranged from undetectable to the highest values measured for 10 CYP genes studied (Yengi et al. 2003) and CYP2B6 transcription remained undetected in native human skin, primary human keratinocytes, fibroblasts, and Langerhans cells in the study by Saeki et al. (2002), as well as in the EpiDerm™ and StrataTest^®^ models (Rolsted et al. 2008; Rassmussen et al. 2011). CYP2B6 protein was localized in the suprabasal cell layers of human foreskin epidermis (Baron et al. 2001) and to the epidermis, sebaceous glands, and hair follicles of adult skin (Baron et al. 1983). Activity for the CYP2B prototypic substrate PROD was below the LOD (<0.2 pmol/min/mg protein) in human skin and in the EpiDerm™ model (Götz et al. 2012a) and below the LOD (4 pmol/min/mg protein) in the StrataTest^®^ model (Guth 2013). The CYP2B inducers phenobarbital (Rolsted et al. 2008, human skin), rifampicin, CITCO (6-(4-Chlorophenyl)imidazo[2,1-b][1,3]thiazole-5-carbaldehyde-*O*-(3,4-dichlorobenzyl)oxime), and cyclophosphamide were also unsuccessful in elevating PROD activity (Götz et al. 2012a, EpiDerm™ model). The CYP2B6 protein is (as found in skin or other systems) active toward several physiological substrates (all-*trans*-retinoic acid, 17β-estradiol, estrone, testosterone) as well as xenobiotics (coumarin, nicotine, ochratoxin A, cyclophosphamide (Ekins and Wrighton 1999; Oesch-Bartlomowicz and Oesch 2007). Phenobarbital increased the CYP2B-catalyzed PROD activity in proliferating human skin keratinocyte cultures (Baron et al. 2001).

*CYP2C subfamily*. CYP2 transcripts and proteins are most often expressed in differentiated keratinocytes comprising the outer suprabasal cell layers of the epidermis (Du et al. 2004). CYP2C9, CYP2C18, and CYP2C19 transcripts were detected in all 27 human full-thickness skin punch biopsies (epidermis and dermis) analyzed (Yengi et al. 2003). Gonzalez et al. (2001) detected CYP2C19 transcripts in clofibrate-treated, but not in control, proliferating human keratinocyte monolayer cultures. CYP2C19 heterozygosity was associated with increased risk of late-onset psoriasis, but was protective against psoriatic arthritis (Richter-Hintz et al. 2003). CYP2C proteins (as found in skin or other systems) metabolize many physiological (2C9: 9-*cis*-and all-*trans*-retinoic acids, arachidonic and linoleic acids, 5-alpha-androstane-3-alpha, 17-beta-diol; 2C18: all-*trans*-retinoic acid, progesterone; 2C19: 9-*cis*-retinal, arachidonic and linoleic acids, testosterone, progesterone), natural compounds (2C9: capsaicin, limonene, nicotine, diallyldisulfide, galangin, genistein 4′-methyl ether, kaempferide, ochrotoxine A, tamarixetin; 2C18: limonene; 2C19: capsaicin, limonene, nicotine, diallyldisulfide, genistein 4′-methyl ether, ochratoxin A, tetrahydrocannabinol), pharmaceutical drugs (2C9: diclofenac, ibuprofen, indomethacin, phenytoin, tolbutamide, (*S*)-warfarin; 2C19: (*S*)-mephenytoin, diazepam, amitryptiline, omeprazole, lansoprazole, pantoprazole, (*R*)-warfarin) (Du et al. 2004; Oesch-Bartlomowicz and Oesch 2007).

*CYP2D subfamily.* CYP2D6 transcripts were detected in all full-thickness human skin biopsies analyzed (27 individuals) (Yengi et al. 2003). Out of 10 investigated CYP genes, the levels of CYP2D6 transcripts were among the highest. However, CYP2D6 transcription was not detected in microsomes of native human skin, Phenion^®^ FT full-thickness skin model (PFT), or EpiDerm™ (Jäckh et al. 2011), in accordance with the CYP2D6-mediated turnover of bufuralol remaining below the LOQ (1.08 pmol) (Rolsted et al. 2008). CYP2D6 transcripts were detected in dermal fibroblasts but not in proliferating subconfluent cultures of human keratinocytes, implying that the dermis or terminally differentiated keratinocytes that were not represented in the proliferating cultures express CYP2D6 transcripts (Saeki et al. (2002). CYP2D6 protein is (as found in skin or other systems) active toward several physiological substrates (all-*trans*-retinal, progesterone, testosterone, tryptoptamine, 5-methoxytryptamine), natural compounds (aflatoxin B_1_, capsaicin, curcumin, diallyl disulfide, emetine, genisteine 4′-methyl ether, ibogaine, nicotine, ochratoxin A, sparteine), pharmaceutical drugs (bufuralol, debrisoquine, fluoxetin, propranolol, dextromethorphan) (Du et al. 2004; Oesch-Bartlomowicz and Oesch 2007).

*CYP2E subfamily.* CYP2E1 transcripts were expressed constitutively in human keratinocyte cultures (Baron et al. 2001; Gonzalez et al. 2001) and in cultures of human keratinocytes, Langerhans cell, fibroblasts, and melanocytes (Saeki et al. 2002). CYP2E1 protein was observed in human foreskin specimens (preferentially localized to the suprabasal cell layers of the epidermis) (Baron et al. 2001). Constitutive activity toward the prototypical CYP2E1 substrate 4-nitrophenol was observed in the microsomal fraction derived from proliferating human keratinocyte cultures (1.81 nmol 4-nitrocatechol formed/min/mg protein). This activity was modestly (1.8-fold) enhanced by treatment with 0.5 M ethanol (Baron et al. 2001). Activity toward the prototypical CYP2E1 substrate chlorzoxazone was detected in human skin microsomes in two independent studies (Rolsted et al. 2008; Götz et al. 2010). Some physiological (fatty acids [predominantely metabolized to their omega minus one hydroxy-derivatives], 17β-estradiol, estrone, all-*trans*-retinoic acid, phosphatidylcholine, uroporphyrinogen, prostaglandin H2) and natural substrates (aflatoxin B_1_, capsaicin, curcumin, diallyl disulfide, genisteine, genisteine 4′-methyl ether, nicotine, methyl eugenol), pharmaceutical drugs such as chlorzoxazone, and many relatively small xenobiotic molecules such as ethanol, and other alcohols, benzene, short-chain *N*-nitrosamines, are metabolized by CYP2E1 (as found in skin or other systems) (Du et al. 2004; Oesch-Bartlomowicz and Oesch 2007).

*CYP2J2 subfamily*. CYP2J2 transcripts were detected in human keratinocyte cultures differentiated for 6 days (Du et al. 2004). Anti-CYP2J2 antibodies recognized proteins in the keratinocyte cell lysates. CYP2J2 protein metabolizes arachidonic and linoleic acids and also testosterone (Du et al. 2004).

*CYP2R, CYP2U, and CYP2W subfamilies.* CYP2R, CYP2U, and CYP2W transcripts were detected in human keratinocyte cultures differentiated for 6 days (Du et al. 2004). The human CYP2R1 was expressed heterologously and shown to have vitamin D 25-hydroxylase activity (Cheng et al. 2003). It therefore may be involved in epithelial differentiation, because the 1,25-dihydroxy-vitamin D is a prodifferentiating mediator in the epidermis (Bikle and Pillai 1993). Recombinant CYP2U1 is active toward medium- and long-chain fatty acids, and it generates mainly 19- and 20-hydroxyeicosatetraenoic acids from arachidonic acid (Chuang et al. 2004). CYP2W1 substrates include benzphetamine and indole (Li et al. 2009).

*CYP2S subfamily*. CYP2S1 (the only member of this subfamily) is expressed primarily in the epithelial cell types, the highest expression occurring in the epithelia of tissues exposed to the outer environment, such as the respiratory and digestive tracts and the skin (Saarikoski et al. 2005a). Transcripts were found in full-thickness skin punch biopsies (epidermis and dermis) from 26 psoriasis patients and from controls (Smith et al. 2003b). Large interindividual differences were observed. The levels of CYP2S1 (and also of NADPH-CYP reductase) transcripts were greater in the psoriasis lesions than in the surrounding unaffected tissue. CYP2S1 protein was localized to the outermost, differentiated cell layers of human epidermis (Smith et al. 2003b). The human CYP2S1 gene promoter region contains two xenobiotic response elements, identical to those in CYP1 genes, and many retinoic acid receptor consensus half-site sequences (Smith et al. 2003b). Crude coal tar as well as retinoic acid (and also UV light) applied to the skin induced the levels of CYP2S1 transcripts in patients and increased the levels of CYP2S1 protein in skin biopsies. However, seven of thirteen individuals showed no CYP2S1 induction after the treatment with coal tar, although CYP1A1 mRNA was induced in all individuals, indicating that the AHR/ARNT system was functional in all them. CYP2S1 was reported to metabolize naphthalene (Saarikoski et al. 2005b) and generate 4-hydroxy and 5,6-epoxy retinoic acid from all-*trans*-retinoic acid (Smith et al. 2003b). However, these reactions were below detection (<0.05 and <0.003 nmol product formed/min/nmol P450, respectively) using recombinant CYP2S1 (Wu et al. 2006). Recently, Wang and Guengerich (2013) found that CYP2S1 is involved in the reductive biotransformation of carcinogenic aromatic amines and heterocyclic aromatic amines. The *N*-hydroxylamines of 4-aminobiphenyl, 2-naphthylamine, and 2-aminofluorene were reduced by CYP2S1, also under aerobic conditions. Some nitroso and nitro derivatives of the arylamines also were reduced by CYP2S1. None of the amines tested were oxidized by CYP2S1, suggesting that CYP2S1 may be involved in the reductive detoxication of many activated metabolites of carcinogenic aromatic amines and heterocyclic aromatic amines.

##### CYP3 family

*CYP3A4 and CYP3A5*. Immunohistology showed the presence of CYP3A protein in human skin specimens (Baron et al. 2001). Metabolism of testosterone to 6-β-hydroxytestosterone, a prototypical activity of CYP3A, was observed in freshly isolated full-thickness ex vivo abdominal human skin preparations (Gibbs et al. 2007). In proliferating human skin keratinocyte cultures, erythromycin *N*-demethylase, an activity preferentially mediated by CYP3A, was constitutively present and further induced by dexamethasone (Baron et al. 2001). The hydroxylation of typical CYP3A4 substrates such as cyclosporine A was, however, preferentially observed in human dermis-derived cultures (Vickers et al. 1995). In proliferating human skin keratinocyte cultures, RT-PCR showed no constitutive expression but an induction by dexamethasone of CYP3A4 transcripts while CYP3A5 mRNA was constitutively expressed (Baron et al. 2001). As far as comparatively investigated, CYP3A4 and CYP3A5 (84 % amino acid identity!) have almost identical substrate specificities, CYP3A5 with lower rates than CYP3A4. Substrates (found in skin or other systems) include Aflatoxin B_1_, acetaminophen, benzphetamine, nifedipine, midazolam, cortisol, dehydroepiandrosterone, 17β-estadiol, progesterone, testosterone, dexamethasone, ethinylestradiol, and levonorgestrel (Oesch-Bartlomowicz and Oesch 2007).

##### CYP4 family

CYP4B1 transcripts were constitutively present in human skin samples (Baron et al. 2008) and were found in epidermal keratinocyte cultures (Saeki et al. 2002). CYP4B1 RNA is found in the reconstructed human skin model EpiSkin™ at levels similar to the epidermis (Luu-The et al. 2009). CYP4B1 can hydroxylate medium-chain fatty acids and is involved in the bioactivation of many protoxins including the bioactivation of 4-ipomeanol to pneumotoxic metabolites and the conversion of several aromatic amines to reactive intermediates that can cause bladder cancer. The wide tissue distribution of CYP4B1 mRNA in humans combined with the capacity of CYP4B1 enzyme protein to activate protoxins and pre-carcinogens may suggest an important role in environmental and occupational health including skin carcinogenesis.

##### Other CYPs

Also, some CYPs essentially involved in the metabolism of endogenous compounds such as steroidogenic CYPs, retinoic acid-metabolizing CYPs, vitamin D-metabolizing CYPs occasionally become important for xenobiotic metabolism when xenobiotics are in partial structures sufficiently similar to their endogenous substrates. For a discussion of these CYPs in the human skin, see our original review Oesch et al. (2007).

### Non-CYP oxidoreductases (see also Tables 2, 11, 12)

#### Flavin-dependent monooxygenases (FMO)

FMO substrates encompass soft nucleophiles. Most notably, they catalyze the *N*-oxygenation of many secondary and tertiary amines.

**Table 11:** Representative non-CYP-xenobiotic-metabolizing enzyme activities^a^ in human skin and human cells in culture

Substrate (preferentially for)	Skin	Keratinocytes^b^	HaCaT	NCTC 2544	KeratinoSens^®^	LuSens	U937	THP-1
Benzydamine (broad-spectrum FMO substrate)	+				bd	bd	bd	bd
Arachidonic acid (COX)	23.5 ± 8.7^c^	5.1 ± 2.8^c^	0.05 ± 0.03^c^	0.02 ± 0.01^c^				
Ethanol (ADH)	0.3–0.4^d^				bd	bq	bd	bq
Propanal (ALDH)				8 ± 1.5^m^	4.77–16.4^d^	8.04–17.3^d^	bd	bq
2,6-Dichlorophenol indophenol (NQR)	~350^d^	~340^d,e^ ~880/1320^d,f^		134 ± 0.05^d^				
Menadione (NQR)	7–10^d^/~ 11^d,g^	~88^d^	~288^d^					
Fluorescein diacetate (E)	~0.5^h^	3.7^h^			3.20–4.06^h^	1.19–3.55^h^	0.911–1.057^h^	0.869–1.072^h^
4-MU heptanoate (E)	10–32^j^	23^j^						
CDNB (GST)	~290^f,j^	~620^d^; ~50^j^	~47^j^	~40^m^ 167 ± 3^m^/234^m^				
Para-nitrophenol (UGT)		0.016–0.023^j^						
4-MU (UGT)	1.3 ± 0.2^k^	bd to ~1.2^j^	1.99 ± 0.97^j^	~1^j^	bd	bd	bd	bd
PABA (NAT)	0.449 ± 0.175^h^	0.215 ± 0.070^h^ 8 ± 0.5^l^	12.0–44.5^l^		13.3–28.7^h^	10.9–22.6^h^	24.8–60.2^h^	6.00–29.9^h^
Para-toluidine (NAT)	0.63–3.03^d^	0.16 ± 0.08^j^	0.65 ± 0.37^j^	0.35 ± 0.22^j^				

More examples and references in the text

*4-MU* 4-methylumbelliferone, *ADH* alcohol dehydrogenase, *ALDH* aldehyde dehydrogenase, *DCNB* 1-chloro-2,4-dinitrobenzene; *E* esterase, *FMO* flavin-dependent monooxygenase, *bd* below the limit of detection, *bq* below the limit of quantification, *NQR* NADH/NADPH quinone reductase, *PABA* para-aminobenzoic acid

^a^Constitutive; number after slash: induced (highest reported induced activity)

^b^Keratinocytes = Primary keratinocytes in culture (“NHEC,” “normal human epithelial keratinocytes”)

^c^pg prostaglandin E2 formed/min/mg microsomal protein

^d^nmol/min/mg cytosolic protein

^e^Differentiated keratinocytes

^f^Proliferating keratinocytes

^g^Ex vivo epidermis

^h^nmol/min/mg S9 protein

^i^Epidermis (scraped from skin surgical samples)

^j^nmol/min/mg protein, activity determined in culture medium

^k^nmol/min/mg microsomal protein

^l^nmol/min/mg cell lysate protein

^m^nmol/min/mg homogenate protein

##### FMO transcripts

mRNA coding FMOs 1, 3, 4, and 5 exhibit marked interindividual variation in adult human skin. Of the individuals analyzed, 90 % contained FMO5 mRNA and about half contained FMO 1, 3, and 4 mRNA. The amount of each FMO mRNA in skin is much lower than in the organ in which it is most highly expressed, the kidney (for FMO1) and the liver (for the others). In contrast to the latter organs, in the skin, FMO mRNAs are present in amounts similar to, or greater than, CYP mRNAs (Janmohamed et al. 2001). Hu et al. (2010) observed mRNA expression of all 5 FMOs (1–5) in full-thickness human buttock skin and all of these except FMO3 also in the reconstructed human skin model EpiDerm™.

mRNA for each of the FMOs was reported to be localized to the epidermis, sebaceous glands, and hair follicles (Janmohamed et al. 2001). Luu-The et al. (2009) observed that FMO1 was expressed more selectively in the epidermis, FMO2 and FMO3 more selectively in the dermis, and FMO4 and FMO5 almost equally in the total skin, dermis, and epidermis.

Their expression profile in the reconstructed human skin models EpiSkin™ and EpiSkin™ FTM was quite different from that of the epidermis: FMO1, FMO3, and FMO5 transcripts were almost absent in EpiSkin™ while FMO2 transcripts were expressed at a relatively high level and FMO4 transcripts at a low level in both models, the difference in expression level between epidermis and models possibly due to stimulating/inhibitory factors in culture media or secreted by fibroblasts (Luu-The et al. 2009). Janmohamed et al. (2001) observed FMO1 transcripts in the epidermis and dermis of native skin, Phenion^®^ FT (PFT) full-thickness, and EpiDermFT™ (EFT) full-thickness reconstructed human skin models.

HaCaT, an immortalized human keratinocyte cell line, expressed FMO3 and FMO5 mRNAs in amounts similar to those in the whole-skin samples, while FMO1 mRNAs was not detected in HaCaT cells and FMO4 mRNA was considerably higher (Janmohamed et al. 2001).

##### FMO proteins

Western blot analysis showed the presence of FMO3 protein in NHEKs, whereas FMO1 was not detectable (Vyas et al. 2006). Thus, it seems that only FMO3 is involved in the observed bioactivation of sulfamethoxazole and dapsone to their respective hydroxylamine metabolites in human keratinocytes (findings discussed in the first paragraph of the “FMO activities” section just below).

##### FMO activities

The arylamine drugs sulfamethoxazole and dapsone were metabolized in normal human epidermal keratinocytes (NHEKs) by FMO3 to arylhydroxylamines that auto-oxidized to arylnitroso derivatives, which bind to proteins and can act as antigens/immunogens. Neither CYPs nor cyclooxygenases mediate this bioactivation of sulfamethoxazole and dapsone in NHECs. Methimazole, a prototypical substrate of FMOs, inhibited the bioactivation of sulfamethoxazole and dapsone in the NHECs (Vyas et al. 2006).

Diphenylthiourea, a catalytic enhancer used during neoprene synthesis, was bioactivated by FMO-catalyzed *S*-oxygenation (Samuelsson et al. 2011).

FMO activity for benzydamine, a relatively broad-spectrum FMO substrate (especially good substrate for FMO1 and FMO3), was below detection in two keratinocytic cell lines derived from HaCat cells, KeratinoSens^®^ and LuSens, and two dendritic cell lines, U937 und THP-1 (Fabian et al. 2013).

In contrast to the cell lines discussed just above, in three-dimensional, reconstructed human skin models, FMO activities with benzydamine as substrate were observed. Jäckh et al. (2011) found FMO activity toward benzydamine in the microsomal fraction of the epidermal EpiDerm™ and the full-thickness skin model Phenion FT with a *V*_max_ value of 5.95 nmol/min/mg for EpiDerm™ and 4.02 nmol/min/mg for PFT, thus similar for the epidermal and the full-thickness model. Estimated *K*_m_ values of 1.4 mM for EpiDerm™ and 2.0 mM for PFT reflect correlated enzyme affinities. In the human epidermal skin model StratTest^®^, Guth (2013) observed also with benzydamine as substrate in the microsomal fraction FMO activity of 0.5 ± 0.1 nmol/min/mg protein.

#### Cyclooxygenases (COX)

COX are responsible for the formation of prostaglandin (PG) H_2_ and are therefore also called PGH_2_ synthases. Formation of the PGH_2_-derived metabolite PGE_2_ is also taken as a measure of COX activity (Giuliano and Warner 2002). COX-1 and COX-2 are structurally related isozymes. Both can oxidize xenobiotic substances of low redox potential including many phenolic compounds and aromatic amines (Oesch and Arand 1999). In contrast to COX-1, COX-2 is inducible by exposure to inflammatory and neoplastic stimuli, suggesting a role for COX-2 in disease pathogenesis. Evidence also suggests that COX-2- and COX-2-derived prostaglandins play a key role in keratinocyte differentiation (Lee et al. 2003).

##### COX transcripts

COX-2 transcripts were present in untreated human buttock skin full-thickness (4 mm) punch biopsies (Smith et al. 2003a) and markedly increased by treatment with coal tar (Smith et al. 2006). Exposure to the AhR ligand FICZ (6-formylindolo[3,2-b]carbazole) even in the lower picomolar range caused significant COX-2 mRNA induction (Fritsche et al. 2007).

##### COX protein

The presence of COX-2 protein was demonstrated in whole skin and in the three reconstructed human skin models EpiDerm™, EpiSkin™, and SkinEthic™ RHE (Hewitt et al. 2013).

In normal human skin, COX-1 protein is present throughout the epidermis, whereas COX-2 localizes mainly in suprabasal keratinocytes (Buckman et al. 1998; Leong et al. 1996).

##### COX activity

Basal COX activity in human skin microsomes was 23.5 ± 8.7 pg PGE2 formed/min/mg protein (Table 2), much lower in the human keratinocyte-derived cell lines HaCaT (0.05 ± 0.03 pg/min/mg protein) and NCTC (0.02 ± 0.01/min/mg protein), while primary NHEKs exhibited more than 100-fold higher PGE2 production (5.1 ± 2.8 pg/min/mg protein). Basal PGE2 production in intact reconstructed human skin EpiDerm™ tissue was, compared with human skin microsomes, about tenfold lower (3.6 ± 1.9 pg/min/mg protein) and increased after treatment with 3-MC approximately twofold (Götz et al. 2012a).

##### COX induction by UV

The expression of COX-2 was induced in artificial human cornified epidermis (approximately 0.8 cm^2^, devoid of dermal components, obtained from MatTek) exposed to simulated solar light. The UVB and UVA-2 (320–350 nm) regions fully accounted for the induction of COX-2 mRNA and protein as well as the enhanced production of prostaglandin E2. At the protein level, approximately 70 % of the total induction by solar light was due to light in the UVA-2 region. UVA-1 (350–400 nm), visible light, and IR radiation were practically ineffective. COX-2 induction by simulated solar light was attenuated by inhibitors of p38 (MAPK) or of *c*-Jun-*N*-terminal kinases (JNK), whereas inhibition of JNK was sufficient for blocking COX-2 induction by UVA (Mahns et al. 2004).

#### Alcohol dehydrogenase (ADH) and aldehyde dehydrogenase (ALDH)

ADH and ALDH are present in human skin (Cheung et al. 1999; Dudley et al. 2000) and are important for the toxication and detoxication of some compounds that cause allergic contact dermatitis, such as *trans*-cinnamaldehyde and *trans*-cinnamic alcohol (Cheung et al. 2003a; Smith et al. 2000). Cutaneous metabolism includes the detoxication of protein-reactive sensitizing aldehydes to alcohols by ADH or to acids by ALDH, or the activation of nonprotein-reactive alcohols into aldehydes by ADH.

Immunohistochemistry showed in human skin a predominant localization of ADH and ALDH in the epidermis, sebaceous glands, and hair follicles (Cheung et al. 2003b).

##### Alcohol dehydrogenase (ADH)

Mammalian ADHs have been classified as ADH1–ADH6 (Duester et al. 1999). ADH proteins 1–4 have different substrate preferences (ADH5 and ADH6 have been identified only at gene and mRNA levels). ADH1 is primarily involved in the oxidation of primary and secondary aliphatic alcohols, including ethanol, the prosensitizers cinnamic alcohol, and eugenol. ADH2 substrates include many aromatic aldehydes. ADH3 is involved in ω-hydroxy fatty acid metabolism and has a GSH-dependent dehydrogenase activity toward formaldehyde (Cheung et al. 2003b).

*ADH transcripts.* Luu-The et al. (2009) showed that ADH1B (which oxidizes ethanol to acetaldehyde and retinol to retinal) exhibited in human skin by far the highest expression level (∼3 million copies/g total RNA) of all phase I XME investigated in his study, and that it was expressed in the dermis, but neither in the epidermis nor in the reconstructed human skin models EpiSkin™ and EpiSkin™ FTM, although the latter is a full-thickness model from EpiSkin™ that also contains fibroblasts seeded in a collagen matrix.

*ADH protein.* ADH1 protein levels were lower in neonatal foreskin compared with adult breast and abdominal skin, indicating age- and/or site-specific expressions of the enzyme protein (Cheung et al. 1999).

Immunohistochemical staining for ADH2 protein was faint in the epidermis with very little dermal expression. ADH3 protein was the most abundant isoform in skin and was localized to the basal layer and the sublaying dermis (Cheung et al. 1999).

Van Eijl et al. (2012) observed ADH1B, ADH3, and ADH4 proteins in human (healthy females 44 ± 13 years old) whole skin (epidermis and dermis).

In line with the localization of ADH1B transcripts and protein in the dermis, ADH1B protein was not detected in the keratinocyte-derived cell line HaCat (Hewitt et al. 2013).

Hewitt et al. (2013) report on the presence of ADH 3 protein in whole skin and in the reconstructed human skin models EpiDerm™, EpiSkin™, and SkinEthik™ RHE while ADH 1B and ADH 4 proteins were reported to be present in the human skin (dermis), but not in these three reconstructed skin models.

*ADH activity.* Saleem et al. (1984) observed human skin ADH activity toward ethanol as substrate (1.8 U/mg, a value ~200-fold lower than that in the liver). Wilkin and Stewart (1987) reported human cutaneous ADH activities for ethanol as 98 nmol NADH produced/min/mg 25,000 g supernatant protein, for propanol and pentanol as 166 and ~300 nmol/min/mg protein, respectively, suggesting that ADH activities increased with increasing length of the carbon chain.

A *V*_max_ of 0.3–0.4 nmol NADH produced/mg cytosolic protein/min was reported for ADH activity with ethanol as substrate, which was lower compared with 1.1–1.2 in mouse skin (Table 2) (Cheung et al. 2003b).

In two keratinocytic cell lines derived from HaCat cells, KeratinoSens^®^ and LuSens, and two dendritic cell lines, U937 und THP-1, the ADH activity toward ethanol as substrate was below the level of quantification in all three passages of the tested four cell lines, in the majority of passages (7/12) even below detection (Fabian et al. 2013).

In line with the exclusive expression of ADH1 transcripts in the dermis (see above), ADH activity with ethanol as substrate was only detected in dermis-containing reconstructed tissues being absent from cytosolic fractions of EpiDerm™ and also absent from primary keratinocytes (Blatz et al. 2011). Likewise, ADH activity with ethanol as substrate was below the LOD (36 nmol/min/mg protein) in the human epidermal skin model StrataTest^®^ (Guth 2013). This apparent restriction of ADH1 activity to the dermal compartment indicates that the use of full-thickness skin models is preferable in order to obtain information on ADH-mediated bioactivation of primary and secondary aliphatic alcohols including several prosensitizers.

##### Aldehyde dehydrogenase (ALDH)

*ALDH transcripts* Hu et al. (2010) observed mRNA expression of ALDH 1A3, 1B1, 2, 3A1, 3A2, 3B1, 3B2, 7A1 in full-thickness human buttock skin and all of these except ALDH 1B1 and ALDH 3B1 also in the reconstructed human skin model EpiDerm™.

*ALDH proteins* ALDH 2 (substrates: acetaldehyde, propionaldehyde, succinic semialdehyde, glutamate-gamma-semialdehyde, aldehydes from lipid peroxidation) antisera revealed a major band (at ≈55 kDa) in the human skin mitochondrial samples (ALDH 2 protein being ≈12-fold lower in the human skin compared with human liver) (Cheung et al. 2003b).

Van Eijl et al. (2012) observed by proteomics ALDH 1A1, 1L1, 2, 3A2, and 9A1 proteins in human whole skin. Hewitt et al. (2013) reported on the presence of ALDH 2 protein in human whole skin and in the reconstructed human skin models EpiDerm™, EpiSkin™, and SkinEthik™ RHE, but that ALDH 1L1 protein was present in human whole skin but not detected in the reconstructed human skin models EpiDerm, EpiSkin™, and SkinEthik™ RHE, while detection versus non-detection of ALDH 3A2 (detected in whole skin) and 7A1 (not detected in whole skin) was variable between these samples.

*ALDH activities* The human keratinocyte cell line NCTC 2544 ALDH had high activities with propionaldehyde and with benzaldehyde as substrates that were increased by 3-MC (Gelardi et al. 2001).

In the two keratinocytic cell lines, KeratinoSens^®^ and LuSens, and in the two dendritic cell lines, U937 und THP-1, discussed above under *ADH activity,* ALDH activities, determined in the cytosolic fraction with propanal as substrate, were clearly detectable and quantifiable in all three passages of the two keratinocyte cell lines with quite similar activities in the two cell lines (4.77–16.4 nmol NAD consumed/min/mg protein in the KeratinoSens^®^ cells compared with 8.04–17.3 nmol NAD consumed/min/mg protein in the LuSens cells). In the two dendritic cell lines, however, the ALDH activity was in all passages below the LOQ, in the majority of passages (4/6) even below the LOD (Fabian et al. 2013).

In the human epidermal skin model StrataTest^®^, Guth (2013) observed with propanal as substrate in the cytosolic fraction ALDH activity of 3.1 ± 0.8 nmol/min/mg protein, similar to the activities in the cell lines discussed just above.

#### NAD(P)H:quinone reductase (NQR)

NQR (also called NADH/NADPH quinone oxidoreductase NQO; DT-diaphorase) activity may largely protect the human skin from the toxicity of quinones circumventing the formation of semiquinone radicals by a direct two-electron reduction of the quinones.

##### NQR transcripts

Hu et al. (2010) observed mRNA expression of both, NQR1 and NQR2, in full-thickness human buttock skin. NQO-1 transcripts were present in untreated human buttock skin full-thickness (4 mm) punch biopsies and markedly increased by treatment with coal tar (Smith et al. 2006). Warwick et al. (2012) observed in an in vitro model of primary human skin fibroblasts at three different developmental stages (1 month, 2 years, and adult) upon treatment with 5–20 μM sulphoraphane 6–35-fold increases in NQR1 transcription and 3–5-fold increases in response to 5–20 μM catechin.

##### NQR protein

NQR1 protein levels were significantly increased in the human skin fibroblasts from 2-year-old and adult individuals in response to sulphoraphane treatment.

##### NQR activity

NQR activity was very high in human skin and human keratinocytes compared to rodents. In contrast to most XME that have a much lower specific activity in the skin compared with the liver (typically 1–5 %), NQR activity is similar in skin and liver. NQR is highly induced by Ah receptor-binding compounds such as coal tar constituents and 3-MC and also by butylated hydroxytoluene, which does not bind to the Ah receptor (Merk et al. 1991). NQR activity in human reconstructed epidermis was lower than in cultured keratinocytes but similar to that in human epidermis (Harris et al. 2002b).

#### Other oxidoreductases

Some other oxidoreductases essentially involved in the metabolism of endogenous compounds such as steroid 5α-reductase and 7-dehydrocholesterol A7-reductase occasionally become important for xenobiotic metabolism when xenobiotics are in partial structures sufficiently similar to their endogenous substrates. For a discussion of these oxidoreductases in the human skin, see our original review Oesch et al. (2007).

### Hydrolases (see also Table 3, 11, 12)

#### Epoxide hydrolase (EH)

##### EH transcripts and protein

Hu et al. (2010) observed mRNA expression of both, microsomal EH (EH1, EPHX1) and cytosolic EH (EH2, EPHX2), in full-thickness human buttock skin.

Luu-The et al. (2009) showed that EPHX1 transcripts are selectively and highly expressed in the dermis while EPHX2 is more selectively expressed in the epidermis, but at a much lower level. In line with this localization, microsomal EH protein was neither detected in the human keratinocyte-derived cell line HaCat nor in the reconstructed human skin models EpiDerm™, EpiSkin™, and SkinEthik™ RHE (Hewitt et al. 2013).

Soluble EH protein was found by immunohistochemistry in the epithelial cells in the human skin in relatively high amounts as estimated from the high intensity of staining in all 3 of the 3 investigated individuals (Enayetallah et al. 2004).

##### EH activity

Microsomal EH activity was found in human skin biopsy-derived microsomes varying in 6 individuals from 175 to 447 pmol BP 4,5-diol/min/mg microsomal protein (Oesch et al. 1978). Bickers et al. (1984) also showed that human skin (S9 fraction of homogenate obtained from epidermal strips freshly excised from healthy, untreated human skin) had clearly measurable microsomal EH activities (25.1 ± 1.1 S.E. pmol BP 4,5-diol/min/mg S-9 fraction protein). The activity of microsomal EH decreased in the order human skin > mouse skin > rat skin (Oesch et al. 1978). The sequence of the rate of hydration was phenanthrene-9,10-oxide > 7-methylbenz[a]anthracene-5,6-oxide ~ benz[a]anthracene-5,6-oxide ~ BP-4,5-oxide > 3-MC-11,12-oxide > dibenz[a,h]anthracene-5,6-oxide (Oesch et al. 1978), which was the same sequence as in the human (and rat) liver (Bentley et al. 1976). Microsomal EH in skin biopsies from 6 donors varied 2.6-fold (Oesch et al. 1978) and 2.3-fold in fibroblast cultures initiated from skin biopsies from 39 adult Caucasian volunteers (Oesch et al. 1980). No significant differences were observed between males, females, smokers, non-smokers, individuals with lung carcinomas, melanomas, or without tumors (Glatt et al. 1984; Oesch et al. 1980). Human skin microsomal EH had similar properties as the human and the rat liver microsomal EH, except for a much lower amount in the skin compared with the liver (Oesch et al. 1977, 1978).

##### Novel EHs

Decker et al. (2012) recently discovered two novel human epoxide hydrolases: EH3 and EH4. They share 45 % sequence identity, thus representing a new family of mammalian epoxide hydrolases. Quantitative RT-PCR from mouse tissue indicated that the novel membrane-bound EH3 was among 20 organs investigated most highly expressed in skin and stomach. The recombinant enzyme showed a high turnover number with 8,9-, 11,12-, and 14,15-epoxyeicosatrienoic acid (EET), as well as 9,10-epoxyoctadec-11-enoic acid (leukotoxin).

#### Esterases/amidases

Carboxylesterases (CE) catalyze the hydrolysis of esters, amides, thioesters, and carbamates. In humans, two carboxylesterases, CE1 and CE2, are important mediators of xenobiotic metabolism. Both are expressed in the liver, where CE1 greatly exceeds CE2 (Laizure et al. 2013). However, the study by Zhu et al. (2007) suggests that in human skin, only CE2 may be expressed.

##### Esterase transcripts

Zhu et al. (2007) report that in the human keratinocyte cell line HaCaT, only CE2 mRNA was detected.

Although in the extensive study of Hu et al. (2010) on the expression of xenobiotic-metabolizing enzyme transcripts the concordance between full-thickness human bottom skin (FTHBS) and EpiDerm™ tissues was high (83–86 % agreement), one of the relatively few exceptions was CE3, which was detected in EpiDerm™ tissues from 2 out of 4 donors, but not in FTHBS. CE1 and CE2 transcripts were expressed in FTHBS and in all 4 EpiDerm™ samples investigated, CE7 in none of these.

##### Esterase activity

In most studies on esterase-mediated xenobiotic metabolism in the human skin, the molecular nature of the responsible esterase(s) is not ascertained.

*Human skin* Esterases/amidases are highly active in human skin. This is especially important if their substrates are topically applied prodrug esters such as 3-alkyl esters of naltrexone (Pillai et al. 2004) or other opioid antagonists, steroid-derived esters (Gysler et al. 1997, 1999), and other prodrug esters such as ethylnicotinate (Rittirod et al. 1999; Sugibayashi et al. 2004), retinyl palmitate (Boehnlein et al. 1994), vitamin E acetate (Baschong et al. 2001), and many more.

Transdermal metabolism studies on the herbicide, fluroxypyr methylheptyl ester (FPMH), fluroxypyr methyl ester (FPM), and fluroxypyr (FP) during penetration through human skin in vitro showed that both FPM and FPMH were completely metabolized during their passage through human skin in vitro. The only metabolite observed was that of the hydrolysis product, FP, with no parent ester penetrating through the skin. Thus, systemic exposure after skin contact with FPM or FPMH is likely to be to the acid metabolite, FP, only and not to the parent ester. The conversion of the lipophilic esters into the highly hydrophilic metabolite FP allows the FP to then readily pass through the viable tissue beneath the stratum corneum, while the absorption of highly hydrophilic compounds (such as FP) through the entire skin is low because the lipid-rich stratum corneum acts as a diffusion barrier (Hewitt et al. 2000a) (the exact localization of the skin esterases is somewhat controversial: according to Hewitt et al. (2000a) exclusively in the viable tissue, according to Beisson et al. (2001) and Egelrud (1992) also in the stratum corneum). Thus, the percutaneous absorption of these lipophilic esters depends on several factors: (1) the rate and extent of the absorption into the skin and the formation of a reservoir of the lipophilic esters in the stratum corneum (parameters 1). (2) These parameters 1 will affect the rate of the hydrolysis of the esters (parameters 2). (3) Differences in amount, activity, and substrate specificity of the esterases between species and models and for individual compounds compose a third parameter substantially affecting the percutaneous absorption of a given compound in a given system.

Human skin microsomes hydrolyzed the prototypical CE substrates naphthyl acetate and para-nitrophenyl acetate with efficiencies (*V*_max_/*K*_m_) between 1.3 and 4.2 min^−1^ mg^−1^, which were very similar to those of minipig skin micrososmes (1.2–4.2 min^−1^ mg^−1^) while those of rat skin microsomes were considerably lower (580–1,100 min^−1^ mg^−1^). Also, human skin cytosol (2.4–67 min^−1^ mg^−1^) hydrolyzed naphthyl acetate and para-nitrophenyl acetate with efficiencies more similar to those of minipig skin cytosol (18–61 min^−1^ mg^−1^) than those of rat skin microsomes (80–100 min^−1^ mg^−1^) (Prusakiewicz et al. 2006).

Parabens, esters of 4-hydroxybenzoic acid, are hydrolyzed to 4-hydroxybenzoic acid in the human skin by CE as shown by the effects of the CE inhibitors paraoxon and bis-nitrophenylphosphate. Loperamide, a specific inhibitor of human CE2, inhibited butyl- and benzylparaben hydrolysis but not methylparaben or ethylparaben demonstrating that butyl- and benzylparaben are more selective substrates for CE2 in human skin than the other two parabens examined (Jewell et al. 2007).

Alcohols used as solvents in topical dosing toxicokinetic studies can interact metabolically by a transesterification reaction with the substrate of interest (e.g., methylparaben) within the skin (Oh et al. 2002).

*Cells in culture*. Esterases appear to be of much higher impact in human keratinocyte cultures than in excised human whole skin (Gysler et al. 1999). These esterases are active in both, human epidermis-derived keratinocyte monolayers as well as in human dermis-derived fibroblast monolayers, but much less in the latter compared with the former (Gysler et al. 1997; Sugibayashi et al. 2004).

Lamb et al. (1994) investigated the metabolism of a series of substituted pyrazolopyridine ester prodrugs in the human keratinocyte cell line NCTC 2544 and in the at that time as graft replacement therapy for burns patients recently introduced LDE Testskin compared with human skin homogenate. Esterase activity was slightly higher in NCTC 2544 than in skin homogenate but the rank order of activity for each prodrug was similar. Esterase activity of LDE Testskin was much lower compared with the other systems, but again the overall pattern of metabolism was similar.

The human keratinocyte-derived cell line HaCaT effectively hydrolyzed the prodrug ketoprofen ethyl ester, inhibited by 2-chloro-3,4-dimethoxybenzil, a specific inhibitor of CE2 (Zhu et al. 2007) as well as amides such as the peptidomimetic compound L-Ala-4-methoxy-2-naphthylamide (Boderke et al. 2000).

Barker and Clothier (1997) compared 4-methylumbelliferone heptanoate hydrolysis rates in primary breast keratinocytes, NCTC2544 cells, and SVK-14 cells. Activity difference between skin donors ranging from 10–32 nmol/min/mg protein indicated a polymorphic CE expression. Differences between cell lines were ~23 nmol/min/mg protein for NCTC2544 and ~13 nmol per min/mg protein for SVK-14 (determined in the culture medium).

Two keratinocytic cell lines derived from HaCat cells, KeratinoSens^®^ and LuSens, and two dendritic cell lines, U937 und THP-1, clearly had esterase activity in the S9 fractions toward fluorescein diacetate as substrate. The keratinocytic cell lines had with 1.19–4.06 nmol product/min/mg protein a somewhat higher activity than the dendritic cell lines with 0.869–1.07 nmol product/min/mg protein. All four cell lines had remarkable esterase activities, all of them within about one order of magnitude compared with the activity observed in Aroclor 1254-induced rat liver S9 as a positive control (0.869–4.06 nmol product/min/mg protein in the tested cell lines compared with 8.82 ± 0.31 nmol product/min/mg protein in rat liver S9) (Fabian et al. 2013).

*Reconstructed skin models.* Gysler et al. (1999) observed esterase activity for the double ester prednicarbate using the reconstructed human epidermis Skinethic™, Sugibayashi et al. (2004) for ethyl nicotinate using “LSE (living skin equivalent)-high” (Toyobo, Osaka, Japan).

Quantitative comparisons of esterase activities between systems come to somewhat different results:

Nabi et al. (2001) showed that the skin equivalent model TestSkin™ and the epidermis equivalent model EpiDerm™ had esterase activities for vitamin E acetate as substrate, which were similar to each other (0.07 vs. 0.12 nmol per hour per cm^2^) and similar to excised whole human skin (0.18 nmol per hour per cm^2^).

Klipper et al. (2010) reported that prednicarbate was hydrolyzed within the epidermal model EpiDerm™ and the three full-thickness models, EpiDermFT™, Phenion^®^ FT, and AST-2000. Compared with EpiDerm™, turnover rates were higher in full-thickness skin models possessing keratinocytes and fibroblasts.

Studies on the hydrolysis of fluorescein diacetate in several reconstructed human skin models and cells in culture showed that the rate (nmol/min/mg protein) was highest in primary keratinocytes (3.7) followed by Phenion^®^ FT full-thickness model (2.3), EpiDermFT™ full-thickness model (1.3), primary fibroblasts (1.2), EpiDerm™ (0.8), and AST-2000 (0.59). Thus, keratinocytes in culture had the highest esterase activities toward fluorescein diacetate. Activities of skin models were lower, which was more similar to native human skin (Jäckh et al. 2012).

Bätz et al. (2013) compared reconstructed human epidermis EpiDerm™, the respective reconstructed full-thickness human skin EpiDerm-FT™ model, full-thickness Phenion^®^ FT, and excised human skin. In this study, formation of the main metabolite prednisolone from prednicarbate and of fluorescein from fluorescein diacetate ranked as reconstructed human full-thickness skin similar to reconstructed human epidermis > excised human skin and keratinocytes > fibroblasts. The authors conclude that reconstructed human epidermis and reconstructed human full-thickness skin may be useful to quantitatively address esterase activity of human skin in drug development and hazard analysis, although an increased activity compared to native human skin has to be taken into account.

Guth (2013) observed with fluorescein diacetate as substrate in the S9 fraction of the StrataTest^®^ human skin model esterase activity of 3.6 ± 0.1 nmol/min/mg protein (in the same order of magnitude as in rat liver where the corresponding activity was 5.4 ± 0.4 nmol/min/mg protein).

Most recently, Eilstein et al. (2014) reported comparable intrinsic metabolic clearance values (hydrolysis efficiency *V*_max_/*K*_m_ through rearrangement of the Michaelis–Menton equation, assuming substrate concentration is below *K*_m_) (same order of magnitude) for esterase with 4-methylumbelliferyl acetate as substrate in SkinEthik™ RHE, Episkin™, Episkin™ FTM, and human skin. Apparent *V*_max_ values varied between Episkin™ FTM (2,390 ± 132 pmol/mg protein/min), SkinEthic RHE (1,096 ± 257 pmol/mg protein/min), Episkin™ (234 ± 49 pmol/mg protein/min), and human skin (954 ± 348 pmol/mg protein/min).

#### Other hydrolases

Some other hydrolases such as β-glucocerebrosidase occasionally become important for xenobiotic metabolism when xenobiotics are in partial structures sufficiently similar to their endogenous substrates. For a discussion of these other hydrolases in the human skin, see our original review Oesch et al. (2007).

### Conjugating enzymes (see also Tables 11, 12)

#### Glutathione *S*-transferase (GST)

##### GST transcripts

mRNA coding for GSTA4 (but not GSTA1 and GSTA2), GSTM1, and predominantly GSTP1 as well as induction of the latter by UV irradiation was found in healthy human skin. GSTP1 transcripts were considerably (mean 3.7-fold) increased in psoriatic plaques (Smith et al. 2003a).

Vondracek et al. (2001, 2002) using standardized and quantitative, reverse transcription-polymerase chain reaction (StaRT-PCR), and microarray chip techniques found with good agreement between the 2 methodologies that cultured normal human oral keratinocytes (NOK) and the Siman virus 40 T antigen-immortalized oral keratinocyte line SVpgC2a expressed similar levels of GSTM3, GSTP1. In contrast, SVpgC2a exhibited comparatively higher levels of GST M1, 2, 4, 5, and GST T 1 and comparatively lower levels of microsomal GST 1. Generally, the comparison of NOK from 2 individuals indicated relatively similar transcript levels of these enzymes.

Luu-The et al. (2009) found that among all phase II xenobiotic-metabolizing enzymes investigated, in the skin and in the reconstructed human skin models EpiSkin™ and FTM GSTP1, transcripts were expressed at the highest level: 2–3 million copies/µg total RNA in total human skin, human epidermis and human dermis, and 6–7 million copies/µg total RNA in EpiSkin™ and FTM (full-thickness model from EpiSkin™). GSTT1 and GSTM5 transcripts were also observed at relatively high levels with GSTM5 selectively expressed in the dermis and not detected in EpiSkin™ and FTM (Luu-The et al. 1009).

Hu et al. (2010) observed mRNA expression of all investigated GSTs (GSTA1, A4, M1, M2, M3, M4, M5, P1, Z1) in full-thickness human buttock skin and in all human skin reconstructed model EpiDerm™ samples, with the sole exception of GSTM5 mRNA, which was found in only 2 of 4 investigated EpiDerm™ samples.

Warwick et al. (2012) found in an in vitro model of primary human skin fibroblasts of three different developmental stages (1 month, 2 years, and adult) in response to 5–20 μM sulphoraphane a 3- to 10-fold increase in GSTA1 mRNA.

##### GST protein

Immunocytochemistry showed that GST protein was present in human skin and that it was concentrated in the epidermis and sebaceous glands (Pendlington et al. 1994).

GST proteins of the alpha and pi classes (very much predominantly the latter) were observed in human skin cytosol (Raza et al. 1991).

Van Eijl et al. (2012) observed with a detection rate of 100 % GST pi class and GST mu class proteins; with a detection rate of 50 % GST alpha and GST theta; and with a detection rate of 40 % GST omega in human (healthy females 44 ± 13 years old) whole skin (epidermis and dermis) by proteomics using custom-built PROTSIFT software, protein identification based on the presence of at least 2 different tryptic peptides in at least two donors. GST pi was about twofold higher than in liver, the other investigated GSTs 2- to 8-fold lower than those in liver. The high presence of the pi class GSTP1 protein (that catalyzes the conjugation of GSH to a wide number of exogenous and endogenous hydrophobic electrophiles [Ali-Osman et al. 1997]) may suggest glutathione conjugation to be the major detoxication process in the skin. GST kappa, GST zeta, and microsomal GST 1 proteins were detected in the liver, but not in the skin.

In HaCaT cells, GST pi and GST omega proteins were present in similar levels as in whole fresh skin, but GST alpha and GST mu proteins were not detected in HaCaT cells (van Eijl et al. 2012).

In the reconstructed human skin models SkinEthik™ RHE, EpiSkin™, and Epiderm™, the same GST isozymes were observed as in the whole skin except GST theta (Van Eijl et al. (2012).

##### GST activity (see also Table 4)

*Skin*. GST activity was found in human (2- to 4-day-old neonate) foreskin using styrene oxide and 3-MC-11,12-oxide as substrates (Mukhtar and Bresnick 1976). GST activities toward the broad-spectrum GST substrate 1-chloro-2,4-dinitrobenzene (CDNB), BP-4,5-oxide, styrene-7,8-oxide, leukotriene A4, and ethacrynic acid, but not toward bromosulfophthalein and cumene hydroperoxide were observed in human skin cytosol (Raza et al. 1991). In the study by van Eijl et al. (2012), GST activity in skin toward CDNB as a substrate was 91 ± 42 nmol/mg protein/min, which was consistent with the relative levels of the GST alpha, mu, and omega proteins in the skin and was about eightfold lower compared in the same study with the liver (753 ± 134 nmol/mg protein/min). Ademola et al. (1993b) observed in the receptor fluid of human skin flow-through diffusion cells to which butachlor (2-chloro-2,6-diethyl-*N*-(butoxymethyl) acetanilide) had been applied (relative to the dose small amounts of) glutathione conjugates.

Activity toward the broad-spectrum GST substrate CDNB was found in human skin samples to be considerably higher than in rat, pig, and mouse skin (Jewell et al. 2000).

*Cells in culture* (for cell lines, see overview in Table 7). Hirel et al. (1995) observed in adult human keratinocytes cultured in submerged conditions GST activities toward the broad-spectrum substrate CDNB, which after confluence were decreased by 50 %. No major differences were observed between keratinocytes in primary culture and those in second subculture. After freezing, enzyme activities were only slightly reduced, if at all. Hirel et al. (1996) observed in viable human keratinocytes obtained 24–50 h after death GST activity toward CDNB, which was similar as in keratinocytes from freshly obtained surgical samples (around 40 nmol/min/mg protein).

In human keratinocytes in primary cultures, the only GST expressed was GST P1 and its activity was inducible in correlation with the differentiation degree (Hirel et al. 1996; Harris et al. 2002a). The GST constitutive activity toward several substrates also increased as the keratinocytes differentiated in culture (Blacker et al. 1991). These authors concluded from their data that all of the GST isozymic forms noted in whole skin, with the exception of Pi, are of extra-keratinocyte origin.

In the human keratinocyte cell line NCTC 2544, GST activity with CDNB as substrate was highly expressed and inducible by 3-MC (Gelardi et al. 2001).

In the study by Götz et al. (2012b), GST activity for CDNB as substrate was observed in all three keratinocyte monolayer cells investigated—HaCaT, NCTC 2544, and NHEK—with approximately 50 nmol/min/mg total protein and thus in the range of GST activities in skin cytosolic protein.

*Reconstructed skin models*. Pham et al. (1990) already observed in a reconstituted epidermis model from the outer root sheath cells of human hair follicles effective glutathione conjugation for *cis*-stilbene as substrate. Harris et al. (2002a) demonstrated activity toward the broad-spectrum GST substrate CDNB in reconstructed human epidermal models EpiDerm™, EpiSkin™, and SkinEthic™, which was roughly similar as in cultured human keratinocytes NHEK and in human epidermal samples. Hu et al. (2010) observed GST activity toward CDNB in the reconstructed human skin model EpiDerm™, which was not increased after treatment with 3-MC. Götz et al. (2012b) observed in the cytosolic fraction of reconstructed human skin model EpiDerm™ a GST activity toward CDNB as substrate of approximately 62 nmol/min/mg protein (estimated from the figure), which was threefold higher than in untreated whole human skin cytosol (20 ± 6.8 nmol/min/mg protein), but the activity in the unbroken EpiDerm™ was considerably lower (4.2 ± 1.6 nmol/min/mg protein) speculated to probably be due to a lack of bioavailability of substrate to the enzyme in the unbroken model. The GST activity in the microsomal fraction also was about threefold higher in EpiDerm™ compared with whole-skin microsomes. No significant induction was obtained using 3-MC as potential inducer. Most recently, Eilstein et al. (2014) reported comparable intrinsic metabolic clearance values (*V*_max_/*K*_m_) (same order of magnitude) for GST with CDNB as substrate in SkinEthik™ RHE, Episkin™, Episkin™ FTM, and human skin.

*Other aspects* Inhibition of GST by ethacrynic acid increased the intended cytotoxic activity of several drugs used to treat skin cancer such as melphalan for the treatment for melanomas (Hansson et al. 1991).

Glutathione, the cofactor necessary for GST activity, was present in human skin at the level of 18.9 ± 1.0 nmol/cm^2^, which is similar to pig skin (18.6 ± 1.5) but less than rat skin (30.3 ± 2.5; neonatal rat 91 ± 3.8) (Jewell et al. 2000). In cultured human skin fibroblasts, the glutathione concentration reported by different authors varied widely such as 9.7 (4.2–14.3) nmol/mg protein (Levtchenko et al. 2005), 19.9 nmol/mg protein (Cereser et al. 2001), and 39.4 (24.7–70) nmol/mg protein (Okun et al. 2004). In the human fibroblast cell line 1522, the glutathione concentration was 32 nmol/mg protein (Phillips et al. 1986). In human skin keratinocyte cell lines, the glutathione concentrations were similar in different studies: in HaCaT, 65.4 ± 2.6 nmol/mg protein (Inbaraj and Chignell 2004) and 66 ± 3 nmol/mg protein (Jourdan et al. 2004), and in the human keratinocyte cell line NCTC 2544, 70.8 ± 3.8 nmol/mg protein (Aldini et al. 2003).

#### UDP-glucuronosyltransferase (UGT)

##### UGT transcripts

Vondracek et al. (2001, 2002) using standardized and quantitative, reverse transcription-polymerase chain reaction (StaRT-PCR) and microarray chip techniques found with good agreement between the 2 methodologies that cultured normal human oral keratinocytes (NOK) and the Siman virus 40 T antigen-immortalized oral keratinocyte line SVpgC2a expressed transcripts for UGT2 with lower levels in SVpgC2a. Generally, the comparison of NOK from 2 individuals indicated relatively similar transcript levels of these enzymes. Differences between NOK and SVpgC2a may reflect alteration caused by immortalization.

Luu-The et al. (2009) reported that UGT2B28 and UGT2B4 transcripts were expressed at similar low levels in total human skin, human dermis, human epidermis, and in the reconstructed human skin models EpiSkin™ and FTM (full-thickness model from EpiSkin™): UGT1A1, UGT2B17, and UGT2B15 in the same preparations at a very low level of less than 5,000 copies/µg total mRNA, the LOD in their assay.

Hu et al. (2010) observed by affymetrix analysis the presence of UGT1A6 and UGT1A10 transcripts in full-thickness human buttock skin (pool of samples from 10 subjects) and in all 4 investigated EpiDerm™ samples. UGT2A1 transcripts were seen in full-thickness human buttock skin, but in none of the 4 investigated EpiDerm™ samples, and UGT2A3 transcripts in 1 of the 4 investigated EpiDerm™ samples, but not in full-thickness human buttock skin. UGT2B17, UGT2B28, and UGT3A1 transcripts were not detected in any of the investigated samples. UGT1A8 was observed in all 4 investigated EpiDerm™ samples, but was in full-thickness human buttock skin not detected or only at low levels. UGT1 transcripts were the predominantly expressed UGT group in human skin, UGT1A10 being expressed at high levels (Hu et al. 2010).

##### UGT proteins

As shown by immunoblotting, the expression of phenol UGT protein (according to the new nomenclature presumably UGT1A6) was increased in cultured human keratinocytes by 3-MC, dimethylbenz[a]anthracene, and by retinoic acid (while the expression of GST Pi was not affected) (Vecchini et al. 1995).

UGT proteins were not detected in whole skin or any of the reconstructed human skin models EpiDerm™, EpiSkin™, and SkinEthik™ RHE (Hewitt et al. 2013).

##### UGT activity

*Skin.* UGT activity was readily observed in human skin, although for individual compounds the actual activity may depend on substrate specificities of UGT isoenzymes present in the skin. Many compounds in widespread use such as triclosan (2,4,4′-trichloro-2′-hydroxydiphenyl ether) are converted locally in the human skin to the glucuronide following topical application both in vitro and in vivo (Moss et al. 2000). The same pertains to many drugs topically used on the skin such as salicylic acid (Ademola et al. 1993a). Human skin samples also glucuronidated endogenous compounds, e.g., indolylacetic acid was biotransformed to the glucuronide of indole as terminal metabolite (Ademola et al. 1993c).

For 4-methylumbelliferone, a relatively broad-spectrum UGT substrate, preferential substrate for UGT1 (Tukey and Strassburg 2000), UGT rates in ex vivo human skin microsomes were 1.3 ± 0.2 nmol/min/mg microsomal protein (Table 5) (Götz et al. 2012b).

On the other hand, morphine is only glucuronidated in traces by human skin microsomes, indicating that the biotransformation in the skin can be neglected when morphine is applied topically to humans (Heilmann et al. 2012).

In contrast to most other cutaneous xenobiotic-metabolizing enzymes, UGT activity (toward 4-nitrophenol and bilirubin) was localized (by immunofluorescence) to the stratum corneum (Peters et al. 1987).

*Cells in culture* (for cell lines, see overview in Table 7). Hirel et al. (1996) observed in viable human keratinocytes obtained 24–50 h after death UGT activities toward para-nitrophenol as substrate, which were 16.7 ± 6.7 pmol/min/mg protein in keratinocytes from sun-protected areas and 23.3 ± 10.0 pmol/min/mg protein in keratinocytes from sun-exposed areas (enzyme activity determined in culture medium).

Götz et al. (2012b) found UGT activities toward the UGT1 prototypical substrate 4-methylumbelliferone in three keratinocyte cell types in culture: HaCaT, NCTC 2544, and NHEK. The activities were in the range of those measured in human skin microsomes. The highest activity was observed in HaCaT cells (1.99 ± 0.97 nmol/min/mg protein) (enzyme activity determined in culture medium). In this study, no significant induction by 3-MC (0.25–10 µM) was observed.

In two keratinocytic cell lines, KeratinoSens^®^ and LuSens, and two dendritic cell lines, U937 und THP-1, the UGT activity toward the preferential UGT1 substrate 4-methylumbelliferone was below the LOD (0.173 nmol product/min/mg protein) in all three passages of the tested four cell lines (Fabian et al. 2013). As discussed just above, Götz et al. (2012b) reported that they were able to determine UGT activity toward 4-methylumbelliferone as substrate in HaCaT cells, and Götz et al. (2012b) reported that UGT activity determined with 4-methylumbelliferone as substrate was measurable in microsomes of ex vivo human skin (1.3 ± 0.2 nmol/min/mg protein).

The cofactor necessary for UGT activity, UDP-glucuronic acid, was reported to occur in fibroblast cultures established from human embryonic skin (and used between passages 5 and 20) at 38 ± 1.3 nmol/mg DNA (Särnstrand et al. 1987).

*Reconstructed skin models.* Pham et al. (1990) already observed in a reconstituted epidermis model from the outer root sheath cells of human hair follicles effective glucuronidation of 1-naphthol and bilirubin.

The extent of glucuronidation of salicylic acid was relatively similar in an early 3-dimensional reconstruction of viable human skin supported by a collagen matrix containing fibroblasts overlaid with epidermal keratinocytes (called “living skin equivalent”) compared with human skin and compared with basal human keratinocytes in monolayer culture (0.9 ± 0.01 vs. 0.75 ± 0.3 vs. 1.4 ± 0.1 % of parent compound) (Ademola et al. 1993a).

Hu et al. (2010) reported “significant” (about 35–50 % conversion) UGT activity with 4-methylumbelliferone as a substrate in EpiDerm™ cultures from three donors determined by following the reduction in substrate in the culture medium. The rates were not increased after pretreatment with 3-MC.

Jäckh et al. (2011) observed in the microsomal fractions of EpiDerm™ glucuronidation activity toward 4-methylumbelliferone of 1.98 nmol/min/mg protein and 16-fold lower activity for the full-thickness model PFT (0.12 nmol/min/mg protein).

In microsomes derived from the reconstructed human skin model EpiDerm™, UGT activities toward 4-methylumbelliferone were 1.8 ± 0.2 nmol/min/mg protein, quite similar to the activities in ex vivo human skin microsomes discussed above (1.3 ± 0.2 nmol/min/mg protein) and quite similar to the activities with human liver microsomes (2.7 ± 0.1 nmol/min/mg protein). However, UGT activity in whole intact EpiDerm™ was 0.09 ± 0.03 nmol/min/mg protein, i.e., roughly 20 times lower than those in the microsomal fraction. Systemic treatment with 0.2–10 µM 3-MC elicited no relevant induction of UGT activity in EpiDerm™ (Götz et al. 2012b).

In the human epidermal skin model StrataTest^®^, UGT activity with 4-methylumbelliferone as substrate was in the microsomal fraction below the LOD of 0.5 nmol/min/mg protein and UGT activity with 4-hydroxybiphenyl as substrate was below the LOD of 1,457 ∆-fluorescence units/min/mg protein (Guth 2013).

Most recently, Eilstein et al. (2014) reported comparable intrinsic metabolic clearance values (conjugation efficiency *V*_max_/*K*_m_ through rearrangement of the Michaelis–Menton equation, assuming substrate concentration is below *K*_m_) (same order of magnitude) for UGT with 4-methylumbelliferone as substrate in SkinEthik™ RHE, Episkin™, Episkin™ FTM, and human skin.

#### Sulfotransferase (SULT)

##### SULT transcripts

Dooley et al. (2000) report that they found SULT 1A1 and SULT1A3 transcripts ubiquitously in human epithelial tissues and cultured cells, and in the skin, in addition, SULT1E1, and SULT2B1a/b transcripts.

Vondracek et al. (2002) using standardized and quantitative, reverse transcription-polymerase chain reaction (StaRT-PCR) and microarray chip techniques found in cultured normal human oral keratinocytes (NOK) and the Siman virus 40 T antigen-immortalized oral keratinocyte line SVpgC2a good agreement between the 2 methodologies. The 2 cell types expressed similar levels of SULT1A1 and SULT1A3 transcripts. Generally, the comparison of NOK from 2 individuals indicated relatively similar transcript levels of these enzymes.

In the study by Luu-The et al. (2009), the second highest expression level of all xenobiotic-metabolizing enzyme transcripts investigated was observed for SULT2B1b, a cholesterol and DHEA sulfonation enzyme. mRNA was observed very much predominantly in the epidermis (1–2 million copies/µg total RNA) and at much lower level in the dermis (around 100,000 copies/µg total RNA). mRNA expression in the reconstructed human skin models EpiSkin™ and EpiSkin™ FTM (full-thickness model from EpiSkin™) was similar as in the epidermis (around 2 million copies/µg total RNA). The transcripts for the phenol sulfotransferase SULT1A1 (which catalyzes the sulfate conjugation of catecholamines, phenolic drugs, and neurotransmitters) were also expressed at relatively high levels and selectively in the dermis with low levels in the reconstructed human skin model FTM and very low levels in EpiSkin™. Transcripts for the estrogen sulfotransferase SULT1E1 were observed at low levels in total human skin, human epidermis, Episkin™, and EpiSkin™ FTM, and at very low levels in the human dermis. SULT1B1 and SULT2A1 were not detected in total human skin, human epidermis, EpiSkin™, or FTM (Luu-The et al. 2009).

Hu et al. (2010) observed mRNA expression of SULT1A1, 1A4, 1E1, and 2B1 in full-thickness human buttock skin and in all four reconstructed human skin model EpiDerm™ samples investigated, SULT1A2 in full-thickness human buttock skin, but in none of four EpiDerm™ samples, and SULT1B1, 1C2, 1C4, 2A1, and 4A1 neither in full-thickness human buttock skin nor in any of the four EpiDerm™ samples investigated. SULT2B1 mRNA was expressed at high levels.

##### SULT protein

Falany et al. (2006) report on the presence of SULT2B1b protein, selective for the sulfation of 3β-hydroxysteroids such as dehydroepiandrosterone and pregnenolone, and for cholesterol sulfation, in human skin, but van Eijl et al. (2012) did not observe SULT2B1 protein in human (healthy females 44 ± 13 years old) whole skin (epidermis and dermis) by proteomics using custom-built PROTSIFT software, protein identification based on the presence of at least 2 different tryptic peptides in at least two donors. However, SULT2B1 protein was found in all reconstructed human skin models investigated in their study (EpiDerm™, EpiSkin™, SkinEthik™ RHE) (van Eijl et al. 2012) (the divergence between occurrence in the reconstructed skin models but not in whole skin was confirmed by Hewitt et al. (2013), where in the text the protein is repetitively called SULT1B1 but from Table 2 and context quite obviously SULT2B1 is meant).

##### SULT activity

Many compounds in widespread use such as triclosan (2,4,4′-trichloro-2′-hydroxydiphenyl ether) are converted locally in the human skin to the sulfate following topical application both in vitro and in vivo (Moss et al. 2000). The pesticide Propoxur (2-isopropoxyphenyl *N*-methylcarbamate) applied in vitro to skin from human, rabbit, and pig yielded 2-isopropoxyphenol (IPP), followed by phase II conjugation. In human skin, only sulfate conjugation was observed (while in pig skin, glucuronides and sulfates were formed in equal amounts, and for rabbit skin, glucuronidation was the major route of conjugation with minor amounts of the sulfate conjugate generated) (van de Sandt et al. 1993).

It has also been shown that Minoxidil, an antihypertensive agent and hair growth promoter, is metabolized by sulfation to the active compound, minoxidil sulfate by at least four human SULT activities (Anderson et al. 1998; Dooley 1999).

Hirel et al. (1995) observed in adult human keratinocytes cultured in submerged conditions sulfotransferase activity for paracetamol as substrate. After confluence, the activity was (slightly) decreased. No major differences were observed between keratinocytes in primary culture and those in second subculture. After freezing, the activity was only slightly reduced, if at all. Hirel et al. (1996) observed in viable human keratinocytes obtained 24–50 h after death SULT activities toward paracetamol as substrate (around 0.4 pmol/min/mg protein), which were about half compared with keratinocytes freshly obtained from surgery.

Most recently, Eilstein et al. (2014) investigated SULT activities with para-nitrophenol as substrate in SkinEthik™ RHE, Episkin™, Episkin™ FTM, and human skin. SULT activity toward para-nitrophenol was not detected in any of the three skin models investigated and was observed at the LOD in human skin.

In several structures of the skin, including the sebaceous glands, androgen receptors are present. Androgens regulate the activity of the sebaceous glands. The intracrine steroidogenic activity provides an explanation for the observation that the sebaceous glands develop fully in both boys and girls in utero and at puberty. At puberty, the increase in the secretion of DHEA, and especially of DHEA sulfate, is associated with an increase in sebaceous gland size and sebum production, which frequently leads to the problems of acne (Labrie et al. 2000).

#### *N*-Acetyltransferase (NAT)

##### NAT transcripts

*Human skin.* NAT is expressed in human skin and represents an important contributor to the direct detoxification in the human skin of aromatic amines such as the oxidative hair dye ingredients para-aminophenol and para-phenylenediamine (Yourick and Bronaugh 2000; Kawakubo et al. 2000; Nohynek et al. 2005; Garrigue et al. 2006). Compared with some highly expressed transcripts of XME such as GST Pi (several million copies/µg total RNA in total human skin, human epidermis, and human dermis), NAT expressions were modest (Luu-The et al. 2009). The relatively highest expressed NAT was NAT5 (100,000–200,000 copies/µg total RNA in total human skin, human epidermis, and human dermis), which is part of the major *N*(alpha)-acetyltransferase complex in eukaryotes catalyzing alpha-acetylation of proteins and peptides (Arnesen et al. 2005).

*Cell cultures* (for cell lines, see overview in Table 7). NAT1 mRNA was found in cultures of human keratinocytes and dermal fibroblasts, whereas expression of NAT2 in cultured human keratinocytes was not observed (Reilly et al. 2000) and in cultured human fibroblasts was marginal (Bhaiya et al. 2006). NAT1 but not NAT2 mRNA was found in both neonatal and adult human epidermal keratinocytes (Reilly et al. 2000).

*Reconstructed models.* NAT1 mRNA was expressed at a low level in the reconstructed human skin models EpiSkin™ and FTM, similar to total human skin, human epidermis, and human dermis. NAT2 transcripts were neither observed in the EpiSkin™ and FTM models nor in total human skin, human epidermis, or human dermis (Luu-The et al. 2009). Hu et al. (2010) observed moderate-to-high expression of NAT1 transcripts in all four human skin reconstructed model EpiDerm™ samples investigated and in full-thickness human buttock skin, while the level of expression of NAT2 transcripts was very low in the four human skin reconstructed model EpiDerm™ samples investigated and in full-thickness human buttock skin (Hu et al. 2010).

##### NAT protein

By a proteomic approach using custom-built PROTSIFT software, protein identification based on the presence of at least 2 different tryptic peptides in at least two donors van Eijl et al. (2012) did not detect any NAT isoform in human skin (or liver!) (LOD 3 pmol/mg cytosolic protein). Hewitt et al. (2013) report that neither NAT1 nor NAT2 were detected in human whole skin nor in any of the three reconstructed skin models investigated (EpiDerm™, Episkin™, SkinEthik™RHE). The only NAT isoform protein detected (and only in the EpiSkin™ model) was NAT10 [a protein with histone acetylation activity involved in the regulation of telomerase activity transcriptionally activated by genotoxic agents such as hydrogen peroxide or cisplatin leading to focal accumulation of NAT10 protein in cellular nuclei (Liu et al. 2007)].

##### NAT activity (see also Table 6)

*Human skin*. Several drugs such as benzocaine (Bronaugh et al. 1994) have been reported to be acetylated in human skin.

In ex vivo whole human skin discs, *N*-acetylated products were the only metabolites formed from 4-amino-2-hydroxytoluene, an aromatic amine ingredient in oxidative hair coloring products, and *p*-aminophenol, an aromatic amine known to undergo *N*-acetylation in vivo. When 4-amino-2-hydroxytolune (12.5 mg/kg) was applied topically, *N*-acetyl-4-amino-2-hydroxytoluene was excreted as the major metabolite (66 % of the dose) (in comparison with 37 and 32 % of the same applied dose after i.v. and p.o. administration, respectively) indicating a considerable first-pass effect by the skin in comparison with i.v. and p.o. applications (Goebel et al. 2009).

Blatz et al. (2011) observed in human skin NAT activity of 449 ± 175 pmol/min/mg S9 protein using para-aminobenzoic acid as selective substrate of human NAT1 (Hein et al. 1993; Butcher et al. 2000). NAT1 is notoriously labile, which—at least in part—explains high variability in NAT1 activity determinations (Fabian et al. 2013). Moreover, NAT1 is polymorphic in addition to the more well-known NAT2 polymorphism initially discovered for the human liver (Grant et al. 1997; Minchin et al. 2007).

Götz et al. (2012b) demonstrated in human skin NAT activities using para-toluidine as substrate. Also with this substrate, interindividual variability was high. From 10 individuals tested, four were relatively slow metabolizers of *p*-toluidine (0.63–0.94 nmol/min/mg cytosolic protein) while three other donors showed considerably more rapid conjugation (1.73–3.03 nmol/min/mg cytosolic protein), the remaining three donors displayed intermediate substrate turnover.

*Cells in culture* (for cell lines, see overview in Table 7). Hirel et al. (1995) observed in adult human keratinocytes cultured in submerged conditions NAT activity for procainamide as substrate. After confluence, the activity was (slightly) decreased. No major differences were observed between keratinocytes in primary culture and those in second subculture. After freezing, the activity was only slightly reduced, if at all. Hirel et al. (1996) observed in viable human keratinocytes obtained 24–50 h after death NAT activities toward procainamide as substrate (around 4 pmol/min/mg protein), which were similar to those determined in keratinocytes freshly obtained from surgery.

Neonatal and adult human epidermal keratinocytes (Reilly et al. 2000) were able to *N*-acetylate dapsone and sulfamethoxazole. This is an important detoxication reaction against the delayed-type hypersensitivity caused by these drugs (Reilly et al. 2000): The NAT reaction competes with metabolic toxication to the hydroxylamines, which for both drugs is mediated by CYP2C9, CYP2E1, CYP3A4, and myeloperoxidase (Roychowdhury and Svensson 2005).

In HaCaT cells, *N*-acetylated products were the only metabolites formed from 4-amino-2-hydroxytoluene and *p*-aminophenol (Goebel et al. 2009).

Bonifas et al. (2010) investigated NAT1 activities of three different HaCaT shipments and human primary keratinocytes (NHEK). *N*-acetylation activities for para-aminobenzoic acid were in average 3.4-fold higher in HaCaT compared to NHEK (8 ± 0.5 nmol/mg protein/min) and varied between the HaCaT shipments (range 12.0–44.5 nmol/mg protein/min). This was in good agreement with NAT1 promoter P1-dependent mRNA level and *N*-acetylation of the contact allergen para-phenylenediamine under typical cell-based assay conditions.

Blatz et al. (2011) observed in human keratinocytes NAT activity of 215 ± 70 pmol/min/mg S9 protein using para-aminobenzoic acid as selective substrate of human NAT1. Again, the notorious lability of NAT1 may—at least in part—explain the relatively high variability (Fabian et al. 2013).

Götz et al. (2012b) investigated NAT activities with para-toluidine as substrate in three cell types in culture: HaCaT showed the highest substrate turnover (0.65 ± 0.37 nmol/min/mg protein), the rates were approximately half in NCTC (0.35 ± 0.22 nmol/min/mg protein) and only a forth in NHEK cells (0.16 ± 0.08 nmol/min/mg protein).

In two keratinocytic cell lines, KeratinoSens^®^ and LuSens, and two dendritic cell lines, U937 und THP-1 NAT, activity was observed in the S9 fractions with para-aminobenzoic acid as selective substrate of human NAT1. All three passages of all four investigated cell lines displayed NAT activities (6.00–60.2 nmol product/min/mg protein) (Fabian et al. 2013).

*Reconstructed skin models.* Nohynek et al. (2005) demonstrated the quantitative conversion of the oxidative arylamine hair dye ingredients [^14^C]-para-aminophenol and [^14^C]-para-phenylenediamine in reconstructed human epidermis EpiSkin™, which quantitatively transformed para-aminophenol to *N*-acetyl-*para*-phenylenediamine and para-phenylenediamine to *N*-mono-acetyl-*para*-phenylenediamine and the *N*,*N*’-di-acetylated derivative. No evidence of transformation of *para*-aminophenol or *para*-phenylenediamine to *N*-hydroxylated derivatives was found. The results show efficient NAT activity and suggest that after dermal absorption of para-aminophenol or para-phenylenediamine, humans are systemically exposed to acetylated derivatives.

Jäckh et al. (2011) observed NAT activities toward para-aminobenzoic acid with similar *V*_max_ values of 11.2 nmol/min/mg for the reconstructed human skin model EpiDerm™ and 17.0 nmol/min/mg for the full-thickness model PFT and similar *K*_m_ values of 44 µM for EpiDerm™ and 53 µM for PFT. A high variation in NAT activities was noted between individual skin batches of either the epidermal or the full-thickness skin model. Again, the notorious lability of NAT1 may—at least in part—explain the high variability (Fabian et al. 2013).

In comparison with the rates in skin from the 10 donors discussed above under “*NAT activities*—*Human skin,*” the cytosolic NAT activities (for para-toluidine as substrate) of both EpiDerm™ samples investigated were in the range of the relatively slow metabolizing skin donors. NAT activities in unbroken EpiDerm™ samples (0.56 ± 0.07 nmol/min/mg protein) were similar to those observed in their cytosolic fractions. As expected, NAT activity was not induced by 3-MC (Götz et al. 2012b).

More recently, Guth (2013) observed with para-aminobenzoic acid as substrate in the S9 fraction of the StrataTest^®^ skin model NAT1 activity of 7.2 ± 1.6 nmol/min/mg protein (even higher than in rat liver where the corresponding activity was 3.3 ± 0.1 nmol/min/mg protein).

Most recently, Eilstein et al. (2014) reported comparable intrinsic metabolic clearance values (*V*_max_/*K*_m_) (same order of magnitude) for esterase with para-aminobenzoic acid as substrate in SkinEthik™ RHE, Episkin™, Episkin™ FTM, and human skin. Apparent *V*_max_ somewhat varied between SkinEthic RHE (60 ± 3 pmol/mg protein/min), Episkin™ FTM (47 ± 3 pmol/mg protein/min), Episkin™ (21 ± 2 pmol/mg protein/min), and human skin (33 ± 8 pmol/mg protein/min).

##### Other cutaneous acyltransferases

Also, more specialized acyltransferases exist in human keratinocytes to assure the storage of the important skin retinol through esterification into retinyl esters. This step is catalyzed by two enzymes, acyl-CoA: retinol acyltransferase and lecithin: retinol acyltransferase; their expression is modulated by the differentiation state of the keratinocytes (Antille et al. 2004; Törmä and Vahlquist 1990).

## Overview and conclusions (Here, only key references; for more complete references, see main body of the text)

### Overview on the information gathered in this review

#### A word of caution

Data allowing a solid quantitative comparison of the relative cutaneous xenobiotic-metabolizing potential between the five mammalian species selected in this review are scarce, and data on in vitro systems derived from human tissues in considerable part are still in the course of being collected. The numbers presented in the tables attempt to give a crude comparative picture. However, it appears important to start here with a word of caution that the numbers not only refer to different magnitudes and parameters as given in the footnotes to the individual values in the tables but that the experimental conditions between the individual investigations largely differed. Thus, the numbers presented do not readily lend themselves for solid quantitative comparisons. The main intentions for presenting the numbers are rather to point out that (1) the respective activities are present in the skin or in the skin-derived in vitro systems; (2) the activities are of a sufficient level to lead the authors to present a number (although in many cases it was not determined whether the determined activities were above a [not presented or not determined] LOQ); (3) the numbers may lend themselves for estimating the respective activities as high, moderate, or low; and (4) the standard deviations give one of the many possible indications on the relative trust in the numbers. Opposed to this, the enzymatic activities given in the tables as + signify that the activity is present in the skin, but numerical values are either not given in the original publication or appear uncertain with respect to number, material, or point of reference (e.g., “per mg,” but not defined per mg of what [tissue, protein, DNA]; in several older publications, these points of reference occur, and where they are not stated, it is not possible to guess with confidence).

#### Oxidoreductases

##### Comparison between species

CYPs of the families 1–3 are major contributors to the xenobiotic metabolism in the liver. However, in the skin, their proteins (see text) and their activities (summarized in Table 1) are in part very low, in part below quantification, and in many instances below detection limit. This can be seen in all five species summarized in this review, human, rat, mouse, guinea pig, and pig, although the data pool is scarce. Activities of non-CYP oxidoreductases quite likely may turn out to be relatively more relevant for xenobiotic oxidative metabolism in the skin compared with cutaneous CYPs. Such potentially non-CYP oxidoreductases of potential relevance for cutaneous xenobiotic metabolism include FMOs and COX (Table 2) as well as further with respect to xenobiotic metabolism in the skin almost completely unstudied oxidoreductases including xanthine oxidase and observed, but as yet undefined xenobiotic-metabolizing peroxidases (mentioned in the text). CYPs can oxidatively attack hard nucleophiles and mediate oxidative metabolism at carbon atoms while FMOs are largely restricted to soft nucleophiles as substrates and mainly mediate metabolism at nitrogen and sulfur atoms (for an overview, see Oesch-Bartlomowicz and Oesch 2007). Since, due to the low activities of cutaneous CYPs, the generation of reactive metabolites at carbon atoms may not efficiently be catalyzed by enzymes of the skin, while hydrolases as well as phase II enzymes which predominantly detoxify xenobiotics and their primary metabolites (for an overview also see Oesch-Bartlomowicz and Oesch 2007) are well presented in the skin as exemplary shown in Tables 3, 4, 5, and 6, the human skin as well as the skin of the other species considered in this review may largely be protected against the harmful actions of a large sector of reactive metabolites.

Specifically, activities of xenobiotic-metabolizing CYP activities of families 1–3 are generally low in all five mammalian species considered in this review, often below quantification or even below detection. The scant information available for a comparison of CYPs in the human skin with that of the four other species may be taken to suggest that the data marginally allow for concluding that rat, mouse, and pig may at least qualitatively and perhaps semiquantitatively be reasonable models for human CYP1, CYP2, and CYP3 family-mediated metabolism, the mouse, especially the female mouse, having a tendency for considerably higher CYP activities compared with human. With respect to CYP-mediated xenobiotic metabolism in the porcine skin, an even scarcer data pool is available compared with rat and mouse, while in this respect definitively not sufficient information is disposable for guinea pig skin (Table 1).

The available data for comparing non-CYP-mediated oxidoreductase activities between human skin and that of the other four species researched in this review appear even more limited than the available data on CYP-mediated xenobiotic metabolism in the skin (Table 2). NADH/NADPH quinone reductase activities (with 2,6-dichlorophenolindophenol as substrate) appear within a factor of threefold reasonably similar between human and mouse skin. Alcohol dehydrogenase activities of human, rat, mouse, and guinea pig skin lie within one order of magnitude. For other non-CYP-mediated oxidoreductase activities, no data for a reasonable comparison were found (Table 2).

##### Comparison between human skin in vitro models

For activities of xenobiotic-metabolizing CYP activities of families 1–3, the same general trend as just discussed for the activity in the skin of the five reviewed mammalian species is seen in human cells in culture and also in human reconstructed skin models. These CYP activities are low, in many cases below quantification or even below detection (Tables 9, 10). As may be expected for low enzymatic activities, the range of reported activities toward a given substrate sometimes is quite large, perhaps due to technical difficulties for exact determinations of such low activities, but in part certainly also due to biological variations between individuals and between circumstances. For some specific substrates, some individual human in vitro model appears to be a quite good model in that the range of reported activities in this in vitro model appears to be close to the range in human skin microsomes (taking into account comparable values as indicated by the same footnote in the table), while for some other substrates, this individual human in vitro model is quite different compared with human skin microsomes. So all in all from the limited available information, it may tentatively be concluded that with respect to the activities mediated by CYP families 1–3, these human in vitro models are not worse and not better than non-human mammalian species.

Although with respect to human cells in culture and human reconstructed skin models still more comparative data on non-CYP oxidoreductases are required, the situation is better than the comparative data pool between species. COX activities for arachidonic acid as substrate are known for human skin, primary kerotinocytes in culture, HaCat and NCTC 2544 cell lines, and the reconstructed human skin model EpiDermFT™. Although numerical comparisons must be viewed with great caution, COX-specific activities in primary keratinocytes and in EpiDermFT™ appear to be within an order of magnitude compared with the activity in skin (about 5- to 7-fold lower), but the activities in HaCat and NCTC 2544 cell lines appear to be much lower (500- to 1,000-fold) (Tables 11, 12). Cutaneous COX activities may turn out to be important for the oxidative xenobiotic metabolism in the skin, since COX can metabolize many lipophilic xenobiotic compounds with a low redox potential including many amines (Oesch-Bartlomowicz and Oesch 2007), while cutaneous xenobiotic-metabolizing CYP activities are between low and not detected (Tables 1, 9, 10). For flavin-dependent monooxygenase (FMO), a half-way reasonable comparison appears difficult. More data would definitively be useful, since FMOs are important in the oxidative metabolism of many soft nucleophiles especially at nitrogen and sulfur atoms in xenobiotic compounds (Oesch-Bartlomowicz and Oesch 2007). A meaningful comparison of specific activities of alcohol dehydrogenase (with ethanol as substrate) between human skin versus human cells in culture and reconstructed human skin models does not appear to be possible (Tables 11, 12). The specific activities of NADH/NADPH quinone reductase (NQR) (with 2,6-dichlorophenolindophenol as substrate) appear numerically quite similar in human and in mouse skin (Table 2), and also in primary human keratinocytes (Table 11). In the reconstructed human skin models EpiDerm™, Episkin™, and SkinEthik™ NQR, activities (with menadione as substrate) appear to be within an order of magnitude compared with human skin, the activities in EpiDerm™ and Episkin™ very close to human skin (Table 12).

**Table 12:** Representative non-CYP-xenobiotic-metabolizing enzyme activities^a^ in human skin and human reconstructed skin models^b^

Substrate (preferentially for)	Skin	EpiDerm™	EpiDermFT™	AST-2000	Episkin™	Episkin™ FTM	SkinEthik™ RHE	Phenion^®^ FT	StrataTest^®^
Benzydamine (broad-spectrum FMO substrate)	+	5.95 ± 1.06^c^						4.02 ± 0.38^c^	0.5 ± 0.1^c^
Arachidonic acid (COX)	23.5 ± 8.7^d^	3.6 ± 1.9^d^/~8^d^							
Ethanol (ADH)	0.3–0.4^e^	bd							<36^e^
Propanal (ALDH)									3.1 ± 0.8^e^
2,6-Dichlorophenol indophenol (NQR)	~350^e^								
Menadione (NQR)	7–10^e^/~11^e,f^	~3.6^e^			~11^e^		~44^e^		
4-Methylumbelliferone acetate (E)	0.954 ± 179^g^				0.234 ± 0.049^g^	2.39 ± 0.13^g^	1.096 ± 0.257^g^		
Fluorescein diacetate (E)	~0.5^i^	0.8^i^	1.3^i^	0.59^i^				2.3 to ~5^i^	3.6 ± 0.1^i^
Vitamin E acetate (E)	0.18^h^	0.12^h^							
CDNB (GST)	~290^e,j^ 15 ± 3^k^ (20 ± 6.8^e^)	~410–920^e^ (~62^e^)			~180–430^e^ 51 ± 2^k^	112 ± 12^k^	~390–850^e^ 30 ± 5^k^		
CDNB (microsomal GST)	~3^c^	~11^c^							
4-MU (UGT)	1.3 ± 0.2^c^ 18 ± 11^k^	~1.8–1.98^c^			68 ± 3^k^	54 ± 3^k^	8 ± 1^k^	0.12 ± 0.05^c^	<0.5^c^
Para-nitrophenol (SULT)	Traces				bd	bd	bd		
PABA (NAT)	33 ± 8^k^	11.2 ± 4.1^i^			21 ± 2^k^	47 ± 3^k^	60 ± 3^k^	17.0 ± 5.3^i^	7.2 ± 1.6^i^
Para-toluidine (NAT)	0.63–3.03^e^	~0.68^e^							

More examples and references in the text

*4-MU* 4-methylumbelliferone, *bd* below detection, *CDNB* 1-chloro-2,4-dinitrobenzene, *E* esterase, *FMO* flavin-dependent monooxygenase, *NQR* NADH/NADPH quinone reductase, *PABA* para-aminobenzoic acid

^a^Constitutive; number after slash: induced (highest reported induced activity)

^b^For description of the reconstructed human skin models, see Table 1

^c^nmol/min/mg microsomal protein

^d^pg prostaglandin E2 formed/min/mg microsomal protein

^e^nmol/min/mg cytosolic protein

^f^ Ex vivo epidermis

^g^nmol/min/mg protein (sum of 7,500*g* supernatant + medium)

^h^nmol/h/cm^2^

^i^nmol/min/mg S9 protein

^j^epidermis (scraped from skin surgical samples)

^k^pmol/min/mg protein (sum of 7,500*g* supernatant + medium)

#### Hydrolases

##### Comparison between species

From an anatomical and physiological standpoint, pig skin appears to be relatively close to human skin. Not much is known on xenobiotic-metabolizing enzymes (XME) in pig skin, with the notable exception of esterase activities (Table 3). The data suggest that pig skin is a quite good model for the human skin in this respect. Considerable information on esterase activity is also available for the rat skin, but the pig skin esterase activities appear to be more similar to the human skin compared with the rat skin. Information on skin esterase activities appears scarcer in mouse and scarcest in guinea pig (no information found).

For skin microsomal epoxide hydrolase, the mouse appears to be more similar to the human skin than the rat skin (no information for the pig or guinea pig skin) (Table 3).

##### Comparison between human skin in vitro models

In the cell lines KeratinoSens^®^, LuSens, U937, and THP-1, esterase-specific activities toward fluorescein diacetate as substrate lie numerically within a factor of maximally about fourfold lower compared with primary human keratinocytes (very close in KeratinoSens^®^ and LuSens) and maximally about eightfold higher compared with human skin. In the NCTC 2544 cell line, the esterase activity toward 4-methylumbelliferone heptanoate is practically identical to that in primary human keratinocytes, while the corresponding activities in human skin appear to not have been investigated (Table 11). Thus, all investigated esterase activities in the cell lines KeratinoSens^®^, LuSens, U937, THP-1, and NCTC 2544 lie within an order of magnitude compared with primary human keratinocytes or compared with human skin.

In the several reconstructed human skin models investigated, esterase activities toward 4-methylumbelliferone acetate and fluorescein diacetate as substrates appear to be within an order of magnitude compared with human skin, numerically apparently closest in SkinEthik™ (determined with 4-methylumbelliferone acetate as substrate) and in AST (determined with fluorescein diacetate as substrate). Also with vitamin E acetate as substrate, esterase activity was found to be quite close to human skin (determined in EpiDerm™). Thus, in the investigated reconstructed human skin models, the investigated esterase activities appear to be reasonably close to human skin (Table 12).

For skin epoxide hydrolase too, little information appears to be available in human skin cell lines or reconstructed skin models for any meaningful comparisons.

#### Conjugating enzymes

##### Comparison between species

As far as comparable data are available, cutaneous glutathione *S*-transferase (GST) activities appear quite similar between human, rat, and mouse skin with the possible exceptions of activities for the prototypic substrate for GST Pi, ethacrynic acid (somewhat higher in mouse skin cytosol), and the prototypic substrate for GST A4-4, 4-hydroxynonenal (apparently much lower or absent in rat skin cytosol) (Table 4).

Quantitative data on UGT-glucuronosyltransferase, sulfotransferase, and *N*-acetyltransferase activities for the same substrate in the skin of human and at least one animal species are so scarce that a meaningful comparison appears difficult (Tables 5, 6).

##### Comparison between human skin in vitro models

In cells in culture comparable (i.e., same footnote in Table 11), data exist for glutathione *S*-transferase activity with the broad-spectrum substrate 1-chloro-2,4-dinitrobenzene (CDNB) in primary human keratinocytes and human skin, and again with primary human keratinocytes compared with HaCaT and NCTC 2544 cell lines. Specific activities are similar (up to approximately twofold different) for these cells in culture compared directly or indirectly with human skin (Table 11).

In the reconstructed human skin models EpiDerm™, Episkin™, and SkinEthik™ RHE comparable (i.e., same footnote in Table 12), data lie in average within a factor of about 3 compared with the glutathione *S*-transferase activities in human skin, in Episkin™ FTM at least within the same order of magnitude (Table 12).

UGT-glucuronosyltransferase activities toward 4-methylumbelliferone as substrates appear numerically almost identical between human skin, primary human keratinocytes, and the cell lines HaCat and NCTC 2544. However, the numbers are not comparable, since they were obtained in human skin microsomes as opposed to culture medium for the cells in culture. Yet, the activities in the two cell lines HaCat and NCTC 2544 are close to the activities in the primary human keratinocytes obtained under comparable conditions (apparent numerical differences less than twofold) (Table 11).

Apparent numerical differences in UGT-glucuronosyltransferase activities toward 4-methylumbelliferone as substrate are less than fivefold between comparable (i.e., same footnote in Table 12) activities in reconstructed human skin models (EpiDerm™, Episkin™, Episkin™ FTM, and SkinEthik™ RHE) and human skin with the exception of a numerically apparent slightly more than tenfold difference between Phenion^®^FT and human skin (the result in StrataTest^®^ [below the LOD of 0.5 nmol/min/mg microsomal protein] is compatible with the assumption that also in this reconstructed human skin model, this activity may lie within a factor of 10 compared with human skin of 1.3 ± 0.2 nmol/min/mg microsomal protein) (Table 12).

The comparable (i.e., same footnotes in Table 11) *N*-acetyltransferase-specific activity toward para-aminobenzoic acid as substrate lies in primary human keratinocytes within a factor of about 2 relatively close to that in human skin and the activity of the cell line HaCaT within a factor of about 3 as compared with that in primary human keratinocytes. Thus, these NAT activities are by direct or indirect comparison relatively close to the activity in human skin, while the activity in the cell lines KeratinoSens^®^, LuSens, U937, and THP-1 is considerably higher (Table 11). In addition, the notorious lability of NAT1 may—at least in part—explain the relatively high variability of the NAT activity determinations with para-aminobenzoic acid as substrate (Fabian et al. 2013).

The comparable (i.e., same footnotes in Table 12) *N*-acetyltransferase-specific activities toward para-toluidine and/or para-aminobenzoic acid as substrates allowing for indirect comparison of the investigated reconstructed human skin models (EpiDerm™, Episkin™, Episkin™ FTM, SkinEthik™, Phenion^®^ FT, StrataTest^®^) are within a factor of about two or less compared to human skin (indirect comparison: Phenion^®^ FT and StrataTest^®^ compared to EpiDerm™ with para-aminobenzoic acid as substrate; EpiDerm™ compared to human skin with para-toluidine as substrate) (Table 12).

### Attempt of recommendations derived from the gathered information

*Recommendation* of some individual animal species or some individual in vitro model system with respect to relative meaningfulness for the *predictions of metabolism*-*dependent toxicities and/or desired pharmacological activities* in light of hitherto available information appears difficult. To the above-discussed uncertainties caused by the scarcities of information adds the intrinsic fact that so far all adequately investigated XME which are toxifying some substrates have been found to detoxify other substrates (we established already in 1977 the dual role of microsomal epoxide hydrolase in both, activation and inactivation: Bentley et al. (1977). Toxication versus detoxication depends on the structure of the substrate and can be predicted in several cases, but is as yet unpredictable in many other cases. The difficulties for generalizing predictions are potentiated by the fact that in almost all cases, multiple enzymes with differing affinities to their substrates (be they the starting material or toxic metabolites) are involved in the toxication and in the detoxication of a given toxic species.

If clearly keeping in mind these difficulties, approximations to xenobiotic metabolism-dependent relative appropriateness of different models for predictions of defined toxicities and/or pharmacological activities for distinct purposes may be attempted. Table 13 presents an attempt to approximate an estimation of relative suitability of human skin models with respect to various XME as detailed in the text which follows here below.

**Table 13:** Attempt to approximate an estimation of relative suitability of human skin models with respect to various xenobiotic-metabolizing enzymes^a^

Enzyme	Presumably suitable models^a^
CYP 1 family^b^	Primary human keratinocytes, HaCaT, NCTC 2544, Episkin™, Episkin™ FTM, SkinEthik™ RHE
CYP2B6^c^	Primary human keratinocytes, NCTC 2544
CYP3A^d^	Primary human keratinocytes, HaCaT, NCTC 2544, EpiDerm™
COX^e^	Primary human keratinocytes, EpiDerm™
NQR	Primary human keratinocytes^f^, NCTC 2544^f^, EpiDerm™ ^g^, Episkin™ ^g^, SkinEthik™ RHE^g^
Esterase^h^	Primary human keratinocytes, KeratinoSens^®^, LuSens, U937, THP-1, EpiDerm™, EpiDermFT™, AST-2000, Episkin™, Episkin™ FTM, SkinEthik™RHE, Phenion^®^ FT, StrataTest^®^
Cytosolic GST^i^	Primary human keratinocytes, HaCaT, NCTC 2544, EpiDerm™, Episkin™, Episkin™ FTM, SkinEthik™RHE
Microsomal GST^i^	EpiDerm™
UGT^j^	EpiDerm™, Episkin™, Episkin™ FTM, SkinEthik™ RHE
NAT	Primary human keratinocytes^k^, HaCaT^k^, EpiDerm™ ^l^, Episkin™ ^k^, Episkin™ FTM^k^, SkinEthik™ RHE^k^, Phenion^®^ FT^m^, StrataTest^®m^

*4-MU* 4-methylumbelliferone, *BQOD* benzyloxyquinoline *O*-dealkylase, *CDNB* 1-chloro-2,4-dinitrobenzene, *COX* cyclooxygenase, *CYP* cytochrome P450, *ECOD* 7-ethoxycoumarin *O*-dealkylase, *EROD* 7-ethoxyresorufin *O*-deethylase, *GST* glutathione *S*-transferase, *MROD* 7-methoxyresorufin *O*-demethylase, *NQR* NADH/NADPH quinone reductase, *PABA* para-aminobenzoic acid, *PROD* pentoxyresorufin *O*-depentylase, *NAT* *N*-acetyltransferase, *UGT* UDP-glucuronosyltransferase

^a^According to present state of information. To be taken with great caution, because of very limited comparability of data in most cases (see data and footnotes in Tables 9, 10, 11, 12, and text). Selected for this table if activities in model compared with human skin appear to be within one order of magnitude, underlined if within a factor of 3. For important xenobiotic-metabolizing enzymes not listed in this table, see Table 14

^b^Estimated using EROD^b^ activities. Not applicable for CYP1A2 (based on MROD activities). Reliability of numbers very limited because of technical difficulties of accurate determination of very low CYP activities

^c^Estimated using PROD activities. Reliability of numbers is very limited because of technical difficulties of accurate determination of very low CYP activities

^d^Estimated using BQOD and erythromycin *N*-demethylase activities. Reliability of numbers is very limited because of technical difficulties of accurate determination of very low CYP activities

^e^Estimated using arachidonic acid as substrate

^f^Estimated using 2,6-dichloroindophenol as substrate

^g^Estimated using menadione as substrate

^h^Estimated using 4-methylumbelliferone acetate or fluorescein diacetate as substrate

^i^Estimated using CDNB as substrate

^j^Estimated using 4-MU as substrate

^k^Estimated using PABA as substrate

^l^Estimated using para-toluidine as substrate

^m^Indirect comparison: Phenion^®^ FT and StrataTest^®^ compared to EpiDerm™ with para-aminobenzoic acid as substrate; EpiDerm™ compared to human skin with para-toluidine as substrate

#### Attempt of recommendation of models for xenobiotic metabolism-dependent dermal absorption

Dermal absorption is profoundly influenced by the size and relative lipophilicity/hydrophilicity of the compound or metabolite(s) in question. Hence, models with reasonable predictivities for dermal absorption by human skin should qualitatively and quantitatively possess similar activities of those XME which strongly influence the size and relative lipophilicity/hydrophilicity of the compound in question. Such enzymes include many hydrolases influencing the size of compounds, especially esterases which, in addition of reducing the molecular size of the parent compound, convert relatively lipophilic esters into more hydrophilic alcohols and carboxylic acids (of course, many other XME also substantially influence the size [e.g., glutathione *S*-transferases], lipophilicity/hydrophilicity [e.g., CYPs], or both [e.g., UDP-glucuronosyltransferases]). With respect to these parameters, esterases appear to be especially important, and with respect to *interspecies differences* in esterase activities, the pig skin appears to be closest to human skin, although the scarcity of information regarding the other species covered in this review precludes a firm conclusion. As discussed in more detail above, with respect to in vitro *models,* the investigated esterase-specific activities in the cell lines, KeratinoSens^®^, LuSens, U937, THP-1, and NCTC 2544, and in the reconstructed human skin models, SkinEthik™, AST, EpiDerm™, and StrataTest^®^, were all reasonably close (all within an order of magnitude) compared with human skin. Beside the esterases which especially profoundly influence dermal absorption, practically all XME potentially influence dermal absorption by modulating the lipophilicity/hydrophilicity of xenobiotics and an estimation of the relative importance of the individual enzyme may be gained by considering the structure of the compound in question after which Table 13 may be used in an attempt for choosing the most appropriate model with respect to XME. However, beside paying due attention to xenobiotic metabolism, it has to be kept in mind that the presently available in vitro skin models have a too low barrier function for realistically estimating dermal absorption by the in vivo human skin.

#### Attempt of recommendation of models for xenobiotic metabolism-dependent genotoxicity and sensitization

Metabolic control of reactive species (toxication and detoxication) is with respect to genotoxicity and sensitization largely mediated by the same enzymes. CYPs are the major actors for generation of reactive metabolites, while phases II enzymes predominantly limit their toxicities by inactivation (e.g., glutathione *S*-transferases) or sequestration of their precursors into less toxic or more easily excretable pathways (e.g., UGP-glucuronosyltransferases). Animal species and in vitro systems possessing qualitatively and quantitatively similar activities of those XME which for most structural entities contribute especially much to the control of these reactive metabolites may tentatively be considered as most promising for providing adequate predictions of genotoxic and/or sensitization potential. Of course, structural entities will dictate whether a compound under investigation is a good/moderate/poor or no substrate for an enzyme/isoenzyme present in a model under consideration, and the limitation has to be taken fully into account that all adequately investigated XME have turned out to play dual roles as discussed above.

With respect to species differences, the scant information available may be taken to suggest that the data marginally allow for concluding that the rat and pig may at least qualitatively and perhaps semiquantitatively be reasonable models for human xenobiotic-metabolizing CYPs, while the mouse has a tendency for considerably higher CYP activities compared with human. However, for skin microsomal epoxide hydrolase, the mouse appears to be more similar to the human skin than the rat skin. As far as comparable data are available, cutaneous glutathione *S*-transferase (GST) activities appear quite similar between human, rat, and mouse skin (except the activities for the prototypic GST A4-4 substrate, 4-hydroxynonenal: apparently much lower or absent in rat skin cytosol). Quantitative data on UGT-glucuronosyltransferase, sulfotransferase, and *N*-acetyltransferase activities for the same substrate in the skin of human and at least one animal species are too scarce for a meaningful comparison. Taking these data together, a reasonable approximation to a judgement which animal species is closest to human with respect to the enzymatic control of reactive metabolites will largely depend on the expectation whether based on the structure of the compound in question the first-mentioned enzymes may be limiting for its metabolism and, if the last-mentioned enzymes are expected to also play a relevant role for its metabolism, even an only approximate judgement appears at present not possible.

In human in vitro *models* (cells in culture as well as reconstructed skin models), the activities of the xenobiotic-metabolizing CYPs of families 1–3 are low, in many cases below detection. For some specific substrates, some individual human in vitro model appears to be a quite good model. However, for some other substrates, the same in vitro model possesses quite different activities compared with human skin microsomes. Thus, meaningful comparisons of various models with respect to reactive metabolite-producing CYPs with human skin appear at present not possible. The situation is somewhat better with respect to COX. COX-specific activities with arachidonic acid as substrate in primary keratinocytes and in EpiDermFT™ appear to be within an order of magnitude compared with the activity in skin (about 5- to 7-fold lower) (while the activities in HaCat and NCTC 2544 cell lines appear to be 500- to 1,000-fold lower). Even somewhat better than this is the situation with respect to predominantly reactive metabolite-lowering (inactivating or precursor-sequestering) enzymes. Glutathione *S*-transferase-specific activities for the broad-spectrum substrate 1-chloro-2,4-dinitrobenzene (CDNB) are similar (within a factor of approximately 2) in primary human keratinocytes, in HaCaT and NCTC 2544 cell lines as in human skin. In the reconstructed human skin models, EpiDerm™, Episkin™, Episkin™ FTM, SkinEthik™ RHE comparable glutathione *S*-transferase activities lie within a factor of about 10 compared with human skin (apparently closest in Episkin™ and SkinEthik™ RHE). UGT-glucuronosyltransferase activities toward 4-methylumbelliferone as substrate in the two cell lines HaCat and NCTC 2544 are close to the activities in the primary human keratinocytes (within a factor of about 2) (while the data on human skin microsomes are not comparable). In the reconstructed human skin models EpiDerm™, Episkin™, Episkin™ FTM, and SkinEthik™, the apparent differences in UGT-glucuronosyltransferase activities toward 4-methylumbelliferone as substrate are less than fivefold compared with human skin, slightly more than tenfold between Phenion^®^FT and human skin. The comparable *N*-acetyltransferase-specific activities in primary human keratinocytes, the cell line HaCaT as well as in the reconstructed human skin models EpiDerm™, Episkin™, Episkin™ FTM, SkinEthik™, Phenion^®^ FT, and StrataTest^®^ are relatively close to the activity in human skin (directly or indirectly compared within a factor of about 3).

Thus, comparisons with respect to CYPs appear only borderline possible in large part because of technical difficulties of sufficiently accurate determinations of their very low activities. However, primary human keratinocytes as well as reconstructed human skin models, especially Episkin™ FTM and SkinEthik™ RHE, appear at least with respect to predominantly reactive metabolite-reducing (inactivating or precursor-sequestering) activities in light of the presently available (still relatively scarce) information presumably adequate models for the human skin. In case the structure of the compound in question allows an estimation which ones of these enzymes are relevant for the metabolism of the compound in question, Table 13 may aid in an attempt to approximate an evaluation of relative suitability of individual human skin models for adequately contributing to predictions of genotoxicity and sensitization potential of xenobiotics applied to the skin with respect to various non-CYP-dependent oxidoreductases, hydrolytic and conjugating enzymes.

#### Attempt of recommendation of models for xenobiotic metabolism-dependent skin irritation

XME profoundly influence the predictivity of a skin irritation test system for human when the irritant species is metabolically produced or detoxified. This is typically the case when a little or not irritant ester is converted by esterases to an irritant acid and/or an irritant or irritation augmenting alcohol which, in turn, may be converted by alcohol dehydrogenase and aldehyde dehydrogenase via the corresponding aldehyde to an irritating acid. Thus, beside many other XME, the presence in irritation test systems of esterase, alcohol dehydrogenase, and aldehyde dehydrogenase activities qualitatively and quantitatively similar to the human skin represents especially fundamental prerequisites for correct predictions.

Among the animal species considered in this review, the pig skin appears to be a quite good model for the human skin with respect to esterase activities, although the important limitation must be kept in mind that detailed studies on substrate specificities are lacking. The pig skin esterase activities appear to be more similar to the human skin compared with the rat skin (not enough information on skin esterase activities in mouse and guinea pig skin). Alcohol dehydrogenase activities of human, rat, mouse, and guinea pig skin lie within one order of magnitude.

In the *cell lines* KeratinoSens^®^, LuSens, U937, and THP-1, esterase-specific activities toward fluorescein diacetate as substrate lie within a factor of less than eightfold compared with human skin S9 (very close in U937 and THP-1). Alcohol dehydrogenase and aldehyde dehydrogenase activities in human cell lines cannot readily be compared with those in native human skin.

In the reconstructed human skin models investigated, esterase activities appear to be within an order of magnitude compared with human skin, numerically apparently closest in SkinEthik™RHE (with 4-methylumbelliferone acetate as substrate) and in AST-2000 (with fluorescein diacetate as substrate). Also, with vitamin E acetate as substrate, esterase activity was close to human skin (determined in EpiDerm™). Thus, in the investigated reconstructed human skin models, the investigated esterase activities appear to be reasonably close to human skin. Alcohol dehydrogenase and aldehyde dehydrogenase activities in human reconstructed skin models cannot readily be compared with those in native human skin.

#### Brief summary of the attempt of recommendations derived from the gathered information

Thus, if all above-discussed severe limitations are strictly taken into consideration, the following crude generalizations for the xenobiotic metabolism-related relative appropriateness of test systems for toxicities and/or pharmacological activities of compounds coming in contact with the human skin may be attempted.

For taking into account XME important for *DERMAL ABSORPTION* among the various animal species considered in this review, the pig skin appears to be the closest to human skin. Among the various in vitro *models* considered in this review, the *cell lines* KeratinoSens^®^, LuSens, U937, THP-1, and NCTC 2544 as well as the *reconstructed human skin models* SkinEthik™RHE, AST-2000, EpiDerm™, and StrataTest^®^ were all reasonably close to human skin. However, presently available in vitro skin models have a too low barrier function for realistically estimating dermal absorption by the in vivo human skin.

For taking into account XME important for *REACTIVE* *METABOLITE*-*CONTROLLED GENOTOXICITY/CARCINOGENICITY* as well as *SKIN SENSITIZATION* among the various *animal species* scrutinized in this review, a reasonable approximation to a judgement which animal species is closest to human will largely depend on the expectation whether based on the structure of the compound in question CYPs, microsomal epoxide hydrolase and/or glutathione *S*-transferase may be limiting for its metabolism. If UDP-glucuronosyltransferase, sulfotransferase, and/or *N*-acetyltransferase are expected to also play a relevant role for its metabolism, even an only approximate judgement which animal species is closest to human appears at present not possible.

Among the various in vitro models summarized in this review, primary human keratinocytes as well as reconstructed human skin models, especially Episkin™ FTM and SkinEthik™ RHE, appear at least with respect to predominantly reactive metabolite-reducing (inactivating or precursor-sequestering) activities as presumably adequate models for the human skin.

For taking into account XME important for *SKIN IRRITATION* among the various *animal species* scrutinized in this review, the pig skin appears to be a quite good model for the human skin with respect to esterase activities (but detailed studies on substrate specificities are lacking). Among the various in vitro *models* summarized in this review, the *cell lines* KeratinoSens^®^, LuSens, U937, and THP-1 esterase-specific activities are reasonably close to human skin (U937 and THP-1 very close). In the *reconstructed human skin models* investigated, esterase activities appear to be reasonably close to human skin (especially SkinEthik™RHE, AST-2000, and EpiDerm™). However, alcohol dehydrogenase and aldehyde dehydrogenase activities in the non-human animal species considered in this review, in human cell lines and in human reconstructed skin models cannot readily be compared with those in native human skin.

If for a given compound it can be estimated from its structure which XME may be important for its metabolism, Table 13 presents an attempt to approximate an estimation of relative suitability of human skin models with respect to various XME. However, it has to be kept in mind that on grounds of the still scarce information at the present time, this is a very crude approximation and, especially important, that for most XME, these data do not yet distinguish between isoenzymes, which may substantially vary in their substrate specificities.

### Research gaps

For founded comparisons of cutaneous xenobiotic enzymes between human and non-human mammalian species and also between human skin, human cells in culture, and reconstructed human skin models, the available data pool are not yet comforting; especially, investigations on isoenzymes with different substrate specificities are largely lacking, yet are essential for the predictions of metabolism and metabolism-dependent toxicities as well as pharmacologically desired effects of xenobiotics applied to the human skin.

As evident from the representative data shown in Tables 1, 2, 11, and 12, more research on cutaneous oxidoreductases is highly recommendable; especially, the differences in substrate specificities between CYPs and other oxidoreductases, as well as their individual isoenzymes, deserve attention. CYPs can oxidatively attack hard nucleophiles and mediate oxidative metabolism at carbon atoms while FMOs are largely restricted to soft nucleophiles as substrates and mainly mediate metabolism at nitrogen and sulfur atoms. COX are efficient at oxidizing (lipophilic) xenobiotics of low redox potential, which includes many amines (for an overview, see Oesch-Bartlomowicz and Oesch 2007). The available data for comparing non-CYP-mediated oxidoreductase activities between human skin and that of the other four species researched in this review and also between human skin and reconstructed human skin models are very limited (Tables 2, 12). Further research in this area appears especially recommendable, since these non-CYP-mediated oxidoreductase activities may turn out to have a higher impact in the cutaneous oxidative xenobiotic metabolism compared with the hitherto observed low activities of xenobiotic-metabolizing CYPs in the skin, as pointed out above.

It also becomes evident from the representative information shown in Tables 3, 4, 5, and 6 that the database on hydrolases and on conjugating enzymes in the various species researched in this review is scarce.

Also, it can be realized from the data presented in the first six tables that unfortunately in several cases, data on specialized xenobiotic-metabolizing enzymes appear to be missing for the human skin such that the relevance of these data on these specialized XME in non-human animal models cannot readily be estimated by comparison with the native human skin.

On a trivial technical issue, in many publications, unfortunately some parameters are not investigated or not reported, which are required for a founded comparison, such as essential details concerning subjects, material and experimental procedure, reference point of the reported activity (activity per mg of what? In unbroken material, total homogenate, fraction derived from it?), linearity of metabolic rate with respect to time and with respect to the amount of material investigated or obtained number just assumed to be within linearity and therefore just divided by minutes and by milligrams? Moreover, as already pointed out above, *in most cases,* the limit of detection (LOD) and/or limit of quantification (LOQ) are not given in the original publications (and probably were not determined) such that low activities versus zero activity or “not detected” may have different and ill-defined meanings between different original publications. Especially important research gaps in human models are summarized in Table 14.

**Table 14:** Important research gaps in human models^a^

CYP^b^ 1A2 (almost no data)
CYP2Cs (very few data)
CYP2D6 (almost no data)
CYP2E1 (almost no data)
FMO (very few data)
COX (very few data)
ADH and ALDH (very few data)
EH (almost no data)

*COX* cyclooxygenase, *CYP* cytochrome P450, *ADH* alcohol dehydrogenase, *ALDH* aldehyde dehydrogenase, *EH* epoxide hydrolase, *FMO* flavin-dependent monooxygenase

^a^Primary cells, cell lines, reconstructed skin models compared with human skin

Thus, beside all shortcomings, as discussed in detail above, the available information at least suggests that with respect to some cutaneous XME, some non-human animal species, some cells in culture, and/or some human reconstructed skin models appear to be or promise to become quite reasonable models for predicting metabolism and metabolism-dependent toxicities as well as metabolism-dependent desired pharmacological effects of some structurally related xenobiotics applied to or just coming in contact with the human skin.

## References

[CR1] Adamson B, Schwarz D, Klugston P, Gilmont R, Perry L, Fisher J, Lindblad W, Rees R (1996). Delayed repair: the role of glutathione in a rat incisional wound model. J Surg Res.

[CR2] Ademola JI, Bloom E, Maczulak AE, Maibach H (1993). Skin Penetration and Metabolism: comparative Evaluation of Skin Equivalent, Cell Culture, and Human Skin. J Toxicol-Cut & Ocular Toxicol.

[CR3] Ademola JI, Wester RC, Maibach HI (1993). Absorption and metabolism of 2-chloro-2,6-diethyl-N-(butoxymethyl)acetanilide (butachlor) in human skin in vitro. Toxicol Appl Pharmacol.

[CR4] Ademola JI, Wester RC, Maibach HI (1993). Metabolism of 3-indolylacetic acid during percutaneous absorption in human skin. J Pharm Sci.

[CR5] Agarwal R, Wang ZY, Bik DP, Mukhtar H (1991). Nordihydroguaiaretic acid, an inhibitor of lipoxygenase, also inhibits cytochrome P-450-mediated monooxygenase activity in rat epidermal and hepatic microsomes. Drug Metab Dispos.

[CR6] Agarwal R, Raza H, Allyn DL, Bickers DR, Mukhtar H (1992). Glutathione S-transferase-dependent conjugation of leukotriene A4-methyl ester to leukotriene C4-methyl ester in mammalian skin. Biochem Pharmacol.

[CR7] Akintobi AM, Villano CM, White LA (2007). 2,3,7,8-Tetrachlorodibenzo-p-dioxin (TCDD) exposure of normal human dermal fibroblasts results in AhR-dependent and -independent changes in gene expression. Toxicol Appl Pharmacol.

[CR8] Aldini G, Granata P, Orioloi M, Santaniello E, Carini M (2003). Detoxification of 4-hydroxynonenal (HNE) in keratinocytes: characterization of conjugated metabolites by liquid chromatography/electrospray ionization tandem mass spectrometry. J Mass Spectrom.

[CR9] Ali-Osman F, Akande O, Antoun G, Mao JX, Buolamwini J (1997). Molecular cloning, characterization, and expression in Escherichia coli of full-length cDNAs of three human glutathione S-transferase Pi gene variants. Evidence for differential catalytic activity of the encoded proteins. J Biol Chem.

[CR10] Alvares AP, Kappas A, Levin W, Conney AH (1973). Inducibility of benzo(a)pyrene hydroxylase in human skin by polycyclic hydrocarbons. Clin Pharmacol Ther.

[CR11] Alvares AP, Leigh S, Kappas A, Levin W, Conney AH (1973). Induction of aryl hydrocarbon hydroxylase in human skin. Drug Metab Dispos.

[CR12] Anderson RJ, Kudlacek PE, Clemens DL (1998). Sulfation of minoxidil by multiple human cytosolic sulfotransferases. Chem Biol Interact.

[CR13] Antille C, Tran C, Sorg O, Saurat JH (2004). Topical beta-carotene is converted to retinyl esters in human skin ex vivo and mouse skin *in vivo*. Exp Dermatol.

[CR14] Arnesen T, Gromyko D, Horvli O, Fluge O, Lillehaug J, Varhaug JE (2005). Expression of N-acetyl transferase human and human arrest defective 1 proteins in thyroid neoplasms. Thyroid.

[CR15] Asokan P, Das M, Bik DP, Howard PC, McCoy GD, Rosenkranz HS, Bickers DR, Mukhtar H (1986). Comparative effects of topically applied nitrated arenes and their nonnitrated parent arenes on cutaneous and hepatic drug and carcinogen metabolism in neonatal rats. Toxicol Appl Pharmacol.

[CR16] Bakken PC, Evans VJ, Earle WR, Stevenson RE (1961). Establishment of a strain of human skin cells on chemically defined medium NCTC 109. Am J Hyg.

[CR17] Barker CL, Clothier RH (1997). Human keratinocyte cultures as models of cutaneous esterase activity. Toxicol In Vitro.

[CR18] Baron J, Kawabata TT, Redick JA, Knapp SA, Wick DG, Wallace RB, Jakoby WB, GuengerichFP(1983) Localization of carcinogen-metabolizing enzymes in human and animal tissues. In: Rydström J, Montelius J, Bengtsson M (eds) Extrahepatic drug metabolism and chemical carcinogenesis. Elsevier, Amsterdam, pp 73–88

[CR19] Baron JM, Höller D, Schiffer R, Frankenberg S, Neis M, Merk HF, Jugert FK (2001). Expression of multiple cytochrome p450 enzymes and multidrug resistance-associated transport proteins in human skin keratinocytes. J Invest Dermatol.

[CR20] Baron JM, Wiederholt T, Heise R, Merk HF, Bickers DR (2008). Expression and function of cytochrome p450-dependent enzymes in human skin cells. Curr Med Chem.

[CR21] Baschong W, Artmann C, Hueglin D, Roeding J (2001). Direct evidence for bioconversion of vitamin E acetate into vitamin E: an ex vivo study in viable human skin. J Cosmet Sci.

[CR22] Basketter DA, Pendlington RU, Sarll A, Scholes E (2008). The role of P4501A1 in the activation of prohaptens in skin sensitization. J Invest Dermatol.

[CR23] Bätz FM, Klipper W, Korting HC, Henkler F, Landsiedel R, Luch A, von Fritschen U, Weindl G, Schäfer-Korting M (2013). Esterase activity in excised and reconstructed human skin—biotransformation of prednicarbate and the model dye fluorescein diacetate. Eur J Pharm Biopharm.

[CR283] Bauch C, Kolle SN, Fabian E, Pachel C, Ramirez T, Wiench B, Wruck CJ, van Ravenzwaay B, Landsiedel R (2011) Intralaboratory validation of four in vitro assays for the prediction of the skin sensitizing potential of chemicals. Toxicol In Vitro 25:1162–1168 10.1016/j.tiv.2011.05.030 21669280

[CR24] Beckley-Kartey SA, Hotchkiss SA, Capel M (1997). Comparative in vitro skin absorption and metabolism of coumarin (1,2-benzopyrone) in human, rat, and mouse. Toxicol Appl Pharmacol.

[CR25] Beisson F, Aoubala M, Marull S, Moustacas-Gardies AM, Voultoury R, Verger R, Arondel V (2001). Use of the tape stripping technique for directly quantifying esterase activities in human stratum corneum. Analyt Biochem.

[CR26] Bentley P, Schmassmann H, Sims P, Oesch F (1976). Epoxides derived from various polycyclic hydrocarbons as substrates of homogeneous and microsome-bound epoxide hydratase. A general assay and kinetic properties. Eur J Biochem.

[CR27] Bentley P, Oesch F, Glatt HR (1977). Dual role of epoxide hydratase in both activation and inactivation. Arch Toxicol.

[CR28] Berghard A, Gradin K, Toftgård R (1990). Serum and extracellular calcium modulate induction of cytochrome P-450IA1 in human keratinocytes. J Biol Chem.

[CR29] Bhaiya P, Roychowdhury S, Vyas PM, Doll MA, Hein DW, Svensson CK (2006). Bioactivation, protein haptenation and toxicity of sulfamethoxazole and dapsone in normal human dermal fibroblasts. Toxicol Appl Pharmacol.

[CR30] Bi M, Singh J (2000). Effect of buffer pH, buffer concentration and skin with or without enzyme inhibitors on the stability of [Arg(8)]-vasopressin. Int J Pharm.

[CR31] Bickers DR, Mukhtar H, Dutta-Choudhury T, Marcelo CL, Voorhees JJ (1984). Aryl hydrocarbon hydroxylase, epoxide hydrolase, and benzo[a]-pyrene metabolism in human epidermis: comparative studies in normal subjects and patients with psoriasis. J Invest Dermatol.

[CR32] Bickers DR, Mukhtar H, Meyer LW, Speck WT (1985). Epidermal enzyme-mediated mutagenicity of the skin carcinogen, 2-aminoanthracene. Mutat Res.

[CR33] Bikle DD, Pillai S (1993). Vitamin D, calcium, and epidermal differentiation. Endocr Rev.

[CR34] Blacker KL, Olsen E, Vessey DA, Boyer TD (1991). Characterization of glutathione S-transferase in cultured human keratinocytes. J Invest Dermatol.

[CR35] Blatz V, Jackh C, Reisinger K, Fabian E, van Ravenzwaay B, Landsiedel R (2011). Metabolic activities of reconstructed human skin equivalents and native human skin. Naunyn-Schmied Arch Pharmacol.

[CR36] Bock KW, von Clausbruch UC, Kaufmann R, Lilienblum W, Oesch F, Pfeil H, Platt KL (1980). Functional heterogeneity of UDP-glucuronyltransferase in rat tissues. Biochem Pharmacol.

[CR37] Boderke P, Schittkowski K, Wolf M, Merkle HP (2000). Modeling of diffusion and concurrent metabolism in cutaneous tissue. J Theor Biol.

[CR38] Boehnlein J, Sakr A, Lichtin JL, Bronaugh RL (1994). Characterization of esterase and alcohol dehydrogenase activity in skin. Metabolism of retinyl palmitate to retinol (vitamin A) during percutaneous absorption. Pharm Res.

[CR39] Bonifas J, Hennen J, Dierolf D, Kalmes M, Blömeke B (2010). Evaluation of cytochrome P450 1 (CYP1) and N-acetyltransferase 1 (NAT1) activities in HaCaT cells: implications for the development of in vitro techniques for predictive testing of contact sensitizers. Toxicol In Vitro.

[CR40] Bonina F, Santagati NA, Puglia C (2003). Ketoprofen 1-alkylazacycloalkan-2-one esters as dermal prodrugs: in vivo and in vitro evaluations. Drug Dev Ind Pharm.

[CR41] Boukamp P, Petrussevska RT, Breitkreutz D, Hornung J, Markham A, Fusenig NE (1988). Normal keratinization in a spontaneously immortalized aneuploid human keratinocyte cell line. J Cell Biol.

[CR42] Briggs MH, Briggs M (1973). Induction by topical corticosteroids of skin enzymes metabolizingcarcinogenic hydrocarbons. Br J Dermatol.

[CR43] Brinkmann J, Stolpmann K, Trappe S, Otter T, Genkinger D, Bock U, Liebsch M, Henkler F, Hutzler C, Luch A (2013). Metabolically competent human skin models: activation and genotoxicity of benzo[a]pyrene. Toxicol Sci.

[CR44] Bronaugh RL, Stewart RF, Storm JE (1989). Extent of cutaneous metabolism during percutaneous absorption of xenobiotics. Toxicol Appl Pharmacol.

[CR45] Bronaugh RL, Collier SW, Macpherson SE, Kraeling ME (1994). Influence of metabolism in skin on dosimetry after topical exposure. Environ Health Perspect.

[CR46] Buckman SY, Gresham A, Hale P, Hruza G, Anast J, Masferrer J, Pentland AP (1998). COX-2 expression is induced by UVB exposure in human skin: implications for the development of skin cancer. Carcinogenesis.

[CR47] Butcher NJ, Ilett KF, Minchin RF (2000). Substrate-dependent regula-tion of human arylamine *N*-acetyltransferase-1 in cultured cells. Mol Pharmacol.

[CR48] Cereser C, Boget S, Parvaz P, Revol A (2001). Thiram-induced cytotoxicity is accompanied by a rapid and drastic oxidation of reduced glutathione with consecutive lipid peroxidation and cell death. Toxicology.

[CR49] Chang SK, Williams PL, Dauterman WC, Riviere JE (1994). Percutaneous absorption, dermatopharmacokinetics and related bio-transformation studies of carbaryl, lindane, malathion, and parathion in isolated perfused porcine skin. Toxicology.

[CR50] Cheng JB, Motola DL, Mangelsdorf DJ, Russell DW (2003). De-orphanization of cytochrome P450 2R1: a microsomal vitamin D 25-hydroxylase. J Biol Chem.

[CR51] Cheung C, Smith CK, Hoog JO, Hotchkiss SAM (1999). Expression and localisation ofhuman alcohol and aldehyde dehydrogenase enzymes in skin. Biochem Biophys Res Commun.

[CR52] Cheung C, Hotchkiss SA, Pease CK (2003). Cinnamic compound metabolism in human skin and the role metabolism may play in determining relative sensitisation potency. J Dermatol Sci.

[CR53] Cheung C, Davies NG, Hoog JO, Hotchkiss SA, Smith Pease CK (2003). Species variations in cutaneous alcohol dehydrogenases and aldehyde dehydrogenases may impact on toxicological assessments of alcohols and aldehydes. Toxicology.

[CR54] Chuang SS, Helvig C, Taimi M, Ramshaw HA, Collop AH, Amad M, White JA, Petkovich M, Jones G, Korczak B (2004). CYP2U1, a novel human thymus- and brainspecific cytochrome P450, catalyzes omega- and (omega-1)-hydroxylation of fatty acids. J Biol Chem.

[CR55] Coomes MW, Norling AH, Pohl RJ, Müller D, Fouts JR (1983). Foreign compound metabolism by isolated skin cells from the hairless mouse. J Pharmacol Exp Ther.

[CR56] Cotovio J, Roguet R, Pion FX, Rougier A, Leclaire J (1996). Effect of imidazole derivatives on cytochrome P-450 enzyme activities in a reconstructed human epidermis. Skin Pharmacol.

[CR57] Damen FJM, Mier PD (1982). Cytochrome P-450-dependent 0-deethylase activity in mammalian skin. Brit J Pharmacol.

[CR58] Das M, Bickers DR, Santella RM, Mukhtar H (1985). Altered patterns of cutaneous xenobiotic metabolism in UVB-induced squamous cell carcinoma in SKH-1 hairless mice. J Invest Dermatol.

[CR59] Decker M, Adamska M, Cronin A, Di Giallonardo F, Burgener J, Marowsky A, Falck JR, Morisseau C, Hammock BD, Gruzdev A, Zeldin DC, Arand M (2012). EH3 (ABHD9): the first member of a new epoxide hydrolase family with high activity for fatty acid epoxides. J Lipid Res.

[CR60] Delescluse C, Ledirac N, de Sousa G, Pralavorio M, Botta-Fridlund D, Letreut Y, Rahmani R (1997). Comparative study of CYP1A1 induction by 3-methylcholanthrene in various human hepatic and epidermal cell types. Toxicol In Vitro.

[CR61] Ding S, Lake BG, Friedberg T, Wolf CR (1995). Expression and alternative splicing of the cytochrome P-450 CYP2A7. Biochem J.

[CR62] Dooley TP (1999). Molecular biology of the human cytosolic sulfotransferase gene superfamily implicated in the bioactivation of minoxidil and cholesterol in skin. Exp Dermatol.

[CR63] Dooley TP, Haldeman-Cahill R, Joiner J, Wilborn TW (2000). Expression profiling of human sulfotransferase and sulfatase gene superfamilies in epithelial tissues and cultured cells. Biochem Biophys Res Commun.

[CR86] dos Santos GG, Reinders J, Ouwehand K, Rustemeyer T, Scheper RJ, Gibbs S (2009). Progress on the development of human in vitro dendritic cell based assays for assessment of the sensitizing potential of a compound. Toxicol Appl Pharmacol.

[CR64] dos Santos GG, Spiekstra SW, Sampat-Sardjoepersad SC, Reinders J, Scheper RJ, Gibbs S (2011). A potential in vitro epidermal equivalent assay to determine sensitizer potency. Toxicol In Vitro.

[CR65] Dressler WE, Appelqvist T (2006). Plasma/blood pharmacokinetics and metabolism after dermal exposure to para-aminophenol or para-phenylenediamine. Food Chem Toxicol.

[CR66] Du L, Hoffman SM, Keeney DS (2004). Epidermal CYP2 family cytochromes P450. Toxicol Appl Pharmacol.

[CR67] Du L, Neis MM, Ladd PA, Lanza DL, Yost GS, Keeney DS (2006). Effects of the differentiated keratinocyte phenotype on expression levels of CYP1-4 family genes in human skin cells. Toxicol Appl Pharmacol.

[CR68] Du L, Neis MM, Ladd PA, Keeney DS (2006). Differentiation-specific factors modulate CYP1-4 gene expression in human skin in response to retinoic acid and classic aryl hydrocarbon receptor ligands. J Pharmacol Exp Ther.

[CR69] Du L, Yin H, Morrow DJ, Strobel HW, Keeney DS (2009). 20-Hydroxylation is the CYP-dependent and retinoid-inducible leukotriene B4 inactivation pathway in human and mouse skin cells. Arch Biochem Biophys.

[CR70] Dudley BF, Brimfield AA, Winston GW (2000). Oxidation of thiodiglycol (2,2′-thiobis-ethanol) by alcohol dehydrogenase: comparison of human isoenzymes. J Biochem Mol Toxicol.

[CR71] Duester G, Farres J, Felder MR, Holmes RS, Hoog JO, Pares X, Plapp BV, Yin SJ, Jornvall H (1999). Recommended nomenclature for the vertebrate alcohol dehydrogenase gene family. Biochem Pharmacol.

[CR72] Egelrud T (1992). Stratum corneum chymotryptic enzyme: evidence of its location in the stratum corneum intercellular space. Eur J Dermatol.

[CR73] Eilstein J, Lereaux G, Daronnat E, Meunier JR, Leclaire J, Duche D (2010). Characterization of N-acetyl and glutathione-S-transferase activities in skin and reconstructed human skin models. Drug Metab Rev.

[CR74] Eilstein J, Léreaux G, Budimir N, Hussler G, Wilkinson S, Duché D (2014) Comparison of xenobiotic metabolizing enzyme activities in ex vivo human skin and reconstructed human skin models from SkinEthic. Arch Toxicol 88:1681–1694 10.1007/s00204-014-1218-6 24658324

[CR75] Einolf HJ, Story WT, Marcus CB, Larsen MC, Jefcoate CR, Greenlee WF, Yagi H, Jerina DM, Amin S, Park SS, Gelboin HV, Baird WM (1997). Role of cytochrome P450 enzyme induction in the metabolic activation of benzo[c]phenanthrene in hum an celllines and mouse epidermis. Chem ResToxicol.

[CR76] Ekins S, Wrighton SA (1999). The role of CYP2B6 in human xenobiotic metabolism. Drug Metab Rev.

[CR77] Enayetallah AE, French RA, Thibodeau MS, Grant DF (2004). Distribution of soluble epoxide hydrolase and of cytochrome P450 2C8, 2C9, and 2J2 in human tissues. J Histochem Cytochem.

[CR78] Fabian E, Vogel D, Blatz V, Ramirez T, Kolle S, Eltze T, van Ravenzwaay B, Oesch F, Landsiedel R (2013). Xenobiotic metabolizing enzyme activities in cells used for testing skin sensitization in vitro. Arch Toxicol.

[CR79] Falany CN, He D, Dumas N, Frost AR, Falany JL (2006). Human cytosolic sulfotransferase 2B1: isoform expression, tissue specificity and subcellular localization. J Steroid Biochem Mol Biol.

[CR80] Finnen MJ, Herdman ML, Shuster S (1984). Induction of drug metabolizing enzymes in the skin by topical steroids. J Steroid Biochem.

[CR81] Fisher GJ, Voorhees JJ (1996). Molecular mechanisms of retinoid actions in skin. FASEB J.

[CR82] Flowers MT, Paton CM, O’Byrne SM, Schiesser K, Dawson JA, Blaner WS, Kendziorski C, Ntambi JM (2011). Metabolic changes in skin caused by Scd1 deficiency: a focus on retinol metabolism. PLoS ONE.

[CR83] Friedberg T, Siegert P, Grassow MA, Bartlomowicz B, Oesch F (1990). Studies of the expression of the cytochrome P450IA, P450IIB, and P450IIC gene family in extrahepatic and hepatic tissues. Environ Health Perspect.

[CR84] Friedberg T, Grassow MA, Bartlomowicz-Oesch B, Siegert P, Arand M, Adesnik M, Oesch F (1992). Sequence of a novel cytochrome CYP2B cDNA coding for a protein which is expressed in a sebaceous gland, but not in the liver. Biochem J.

[CR85] Fritsche E, Schäfer C, Calles C, Bernsmann T, Bernshausen T, Wurm M, Hübenthal U, Cline JE, Hajimiragha H, Schroeder P, Klotz LO, Rannug A, Fürst P, Hanenberg H, Abel J, Krutmann J (2007). Lightening up the UV response by identification of the arylhydrocarbon receptor as a cytoplasmatic target for ultraviolet B radiation. Proc Natl Acad Sci USA.

[CR87] Garrigue JL, Ballantyne M, Kumaravel T, Lloyd M, Nohynek GJ, Kirkland D, Toutain H (2006). In vitro genotoxicity of para-phenylenediamine and its N-monoacetyl or N, N’- diacetyl metabolites. Mutat Res.

[CR88] Gelardi A, Morini F, Dusatti F, Penco S, Ferro M (2001). Induction by xenobiotics of phase I and phase II enzyme activities in the human keratinocyte cell line NCTC 2544. Toxicol Invitro.

[CR89] Gibbs S, van de Sandt JJ, Merk HF, Lockley DJ, Pendlington RU, Pease CK (2007). Xenobiotic metabolism in human skin and 3D human skin reconstructs: a review. Curr Drug Metab.

[CR90] Giuliano F, Warner TD (2002). Origins of prostaglandin E2: involvements of cyclooxygenase (COX)-1 and COX-2 in human and rat systems. J Pharmacol Exp Ther.

[CR91] Glatt HR, Mertes I, Wölfel T, Oesch F, Greim H, Jung R, Kramer M, Marquardt H, Oesch F (1984). Epoxide hydrolases in laboratory animals and in man. Biochemical basis of chemical carcinogenesis.

[CR92] Goebel C, Hewitt NJ, Kunze G, Wenker M, Hein DW, Beck H, Skare J (2009). Skin metabolism of aminophenols: human keratinocytes as a suitable in vitro model to qualitatively predict the dermal transformation of 4-amino-2-hydroxytoluene in vivo. Toxicol Appl Pharmacol.

[CR93] Goerz G, Barnstorf W, Winnekendonk G, Bolsen K, Fritsch C, Kalka K, Tsambaos D (1996). Influence of UVA and UVB irradiation on hepatic and cutaneous P450 isoenzymes. Arch Dermatol Res.

[CR94] Gonzalez MC, Marteau C, Franchi J, Migliore-Samour D (2001). Cytochrome P450 4A11 in human keratinocytes: effects of ultraviolet irradiation. Br J Dermatol.

[CR501] Götz C, Ruwiedel K, Pfeiffer R, Hübenthal U, Edwards R, Carmichael P, Aeby P, Goebel C, Pease C, Fritsche E (2010) The COLIPA skin metabolism project: do in vitro alternatives comprise adequate detoxification capacities for chemical testing in skin? In: Poster presented at IUTOX 2010

[CR95] Götz C, Pfeiffer R, Tigges J, Blatz V, Jäckh C, Freytag EM, Fabian E, Landsiedel R, Merk HF, Krutmann J, Edwards RJ, Pease C, Goebel C, Hewitt N, Fritsche E (2012). Xenobiotic metabolism capacities of human skin in comparison with a 3D epidermis model and keratinocyte-based cell culture as in vitro alternatives for chemical testing: activating enzymes (Phase I). Exp Dermatol.

[CR96] Götz C, Pfeiffer R, Tigges J, Ruwiedel K, Hübenthal U, Merk HF, Krutmann J, Edwards RJ, Abel J, Pease C, Goebel C, Hewitt N, Fritsche E (2012). Xenobiotic metabolism capacities of human skin in comparison with a 3D-epidermis model and keratinocyte-based cell culture as in vitro alternatives for chemical testing: phase II enzymes. Exp Dermatol.

[CR97] Grant DM, Hughes NC, Janezic SA, Goodfellow GH, Chen HJ, Gaedigk A, Yu VL, Grewal R (1997). Human acetyltransferase polymorphisms. Mutat Res.

[CR98] Gundert-Remy U, Bernauer U, Blömeke B, Döring B, Fabian E, Goebel C, Hessel S, Jäckh C, Lampen A, Oesch F, Petzinger E, Völkel W, Roos PH (2014). Extrahepatic metabolism at the body’s internal-external interfaces. Drug Metab Rev.

[CR99] Guo JF, Brown R, Rothwell CE, Bernstein IA (1990). Levels of cytochrome P-450-mediated aryl hydrocarbon hydroxylase (AHH) are higher in differentiated than in germinative cutaneous keratinocytes. J Invest Dermatol.

[CR100] Guth K (2013) PhD thesis, FU (Freie Universität) Berlin. http://www.diss.fu-berlin.de/diss/receive/FUDISS_thesis_000000094743?lang=en. Static URL: http://www.diss.fu-berlin.de/diss/receive/FUDISS_thesis_000000094743. NBN: urn:nbn:de:kobv:188-fudissthesis000000094743-4

[CR101] Gysler A, Lange K, Korting HC, Schafer-Korting M (1997). Prednicarbate biotransformation in human foreskin keratinocytes and fibroblasts. Pharm Res.

[CR102] Gysler A, Kleuser B, Sippl W, Lange K, Korting HC, Holtje HD, Korting HC (1999). Skin penetration and metabolism of topical glucocorticoids in reconstructed epidermis and in excised human skin. Pharm Res.

[CR103] Hansson J, Berhane K, Castro VM, Jungnelius U, Mannervik B, Ringborg U (1991). Sensitization of human melanoma cells to the cytotoxic effect of melphalan by the glutathione transferase inhibitor ethacrynic acid. Cancer Res.

[CR104] Hardingham TE, Phelps CF (1968). The tissue content and turnover rates of intermediates in the biosynthesis of glycosaminoglacans in young rat skin. Biochem J.

[CR105] Harris IR, Siefken W, Beck-Oldach K, Brandt M, Wittern KP, Pollet D (2002). Comparison of activities dependent on glutathione S-transferase and cytochrome P-450 IA1 in cultured keratinocytes and reconstructed epidermal models. Skin Pharmacol Appl Skin Physiol.

[CR106] Harris IR, Siefken W, Beck-Oldach K, Wittern KP, Pollet D (2002). NAD(P)H:quinone reductase activity in human epidermal keratinocytes and reconstructed epidermal models. Skin Pharmacol Appl Skin Physiol.

[CR107] Hayden P, Klausner M, Kubilus J, Burnham B, Jackson G (2003). In vitro models of full-thickness human skin (EpiDerm-FT) and airway epithelium (EpiAirway-FT) for toxicology and drug development applications. Vitro Cell Devel Biol Plant.

[CR108] Hayden P, Bolmarcich J, Stolper G, Hu T, Aardema M, Curren R, Klausner M (2006). Xenobiotic metabolizing capabilities of the EpiDerm in vitro human skin equivalent: utility for assessing dermal biotransformation of pharmaceuticals and environmental chemicals. Toxicol Lett.

[CR109] Heilmann S, Küchler S, Schäfer-Korting M (2012). Morphine metabolism in human skin microsomes. Skin Pharmacol Physiol.

[CR110] Hein DW, Doll MA, Rustan TD, Gray K, Feng Y, Ferguson RJ, Grant DM (1993). Metabolic activation and deactivation of arylamine carcinogens by recombinant human NAT1 and polymorphic NAT2 acetyltransferases. Carcinogenesis.

[CR111] Hewitt PG, Perkins J, Hotchkiss SA (2000). Metabolism of fluroxypyr, fluroxypyr methyl ester, and the herbicide fluroxypyr methylheptyl ester. I: during percutaneous absorption through fresh rat and human skin in vitro. Drug Metab Dispos.

[CR112] Hewitt PG, Perkins J, Hotchkiss SA (2000). Metabolism of fluroxypyr, fluroxypyr methyl ester, and the herbicide fluroxypyr methylheptyl ester. II: in rat skin homogenates. Drug Metab Dispos.

[CR113] Hewitt NJ, Edwards RJ, Fritsche E, Goebel C, Aeby P, Scheel J, Reisinger K, Ouédraogo G, Duche D, Eilstein J, Latil A, Kenny J, Moore C, Kuehnl J, Barroso J, Fautz R, Pfuhler S (2013). Use of human in vitro skin models for accurate and ethical risk assessment: metabolic considerations. Toxicol Sci.

[CR114] Hiratsuka A, Yamane H, Yamazaki S, Ozawa N, Watabe T (1997). Subunit Ya-specific glutathione peroxidase activity toward cholesterol 7-hydroperoxides of glutathione S-transferases in cytosols from rat liver and skin. J Biol Chem.

[CR115] Hiratsuka A, Saito H, Hirose K, Watabe T (1999). Marked expression of glutathione S-transferase A4-4 detoxifying 4-hydroxy-2(E)-nonenal in the skin of rats irradiated by ultraviolet B-band light (UVB). Biochem Biophys Res Commun.

[CR116] Hirel B, Chesne C, Pailheret JP, Guillouzo A (1995). In Vitro expression of drug metabolizing enzyme activities in human adult keratinocytes under various culture conditions and their response to inducers. Toxicol In Vitro.

[CR117] Hirel B, Watier E, Chesne C, Patoux-Pibouin M, Guillouzo A (1996). Culture and drug biotransformation capacity of adult human keratinocytes from post-mortem skin. Br J Dermatol.

[CR118] Hoffmann J, Peters P, Frost P, Fuchs HW (2003). Advanced Skin Test 2000 (AST-2000): reconstructed human skin designed for dermatological and pharmaceutical research. Eur J Cell Biol.

[CR119] Hoffmann J, Heisler E, Karpinski S, Losse J, Thomas D, Siefken W, Ahr HJ, Vohr HW, Fuchs HW (2005). Epidermal-skintest 1,000 (EST-1,000)—a new reconstructed epidermis for in vitroskin corrosivity testing. Toxicol In Vitro.

[CR120] Hu T, Khambatta ZS, Hayden PJ, Bolmarcich J, Binder RL, Robinson MK, Carr GJ, Tiesman JP, Jarrold BB, Osborne R, Reichling TD, Nemeth ST, Aardema MJ (2010). Xenobiotic metabolism gene expression in the EpiDerm in vitro 3D human epidermis model compared to human skin. Toxicol In Vitro.

[CR121] Imai T, Takase Y, Iwase H, Hashimoto M (2013). Involvement of Carboxylesterase in Hydrolysis of Propranolol Prodrug during Permeation across Rat Skin. Pharmaceutics.

[CR122] Inbaraj J, Chignell CF (2004). Cytotoxic action of jugolone and plumbagin: a mechanistic study using HaCaT keratinocytes. Chem Res Toxicol.

[CR123] Jäckh C, Blatz V, Fabian E, Guth K, van Ravenzwaay B, Reisinger K, Landsiedel R (2011). Characterization of enzyme activities of cytochrome P450 enzymes, Flavin-dependent monooxygenases, N-acetyltransferases and UDP-glucuronyltransferases in human reconstructed epidermis and full-thickness skin models. Toxicol In Vitro.

[CR124] Jäckh C, Fabian E, van Ravenzwaay B, Landsiedel R (2012). Relevance of xenobiotic enzymes in human skin in vitro models to activate pro-sensitizers. J Immunotoxicol.

[CR125] Jacques C, Perdu E, Dorio C, Bacqueville D, Mavon A, Zalko D (2010). Percutaneous absorption and metabolism of [14C]-ethoxycoumarin in a pig ear skin model. Toxicol In Vitro.

[CR126] Jacques C, Perdu E, Duplan H, Jamin EL, Canlet C, Debrauwer L, Cravedi JP, Mavon A, Zalko D (2010). Disposition and biotransformation of 14C-benzo(a)pyrene in a pig ear skin model: ex vivo and in vitro approaches. Toxicol Lett.

[CR127] Janmohamed A, Dolphin CT, Phillips IR, Shephard EA (2001). Quantification and cellular localization of expression in human skin of genes encoding flavin-containing monooxygenases and cytochromes P450. Biochem Pharmacol.

[CR128] Jewell C, Heylings J, Clowes HM, Williams FM (2000). Percutaneous absorption and metabolism of dinitrochlorobenzene in vitro. Arch Toxicol.

[CR129] Jewell C, Prusakiewicz JJ, Ackermann C, Payne NA, Fate G, Voorman R, Williams FM (2007). Hydrolysis of a series of parabens by skin microsomes and cytosol from human and minipigs and in whole skin in short-term culture. Toxicol Appl Pharmacol.

[CR130] Jourdan E, Jeanne RM, Steiman R, Guiraud P (2004). Zink-metallothionein genoprotective effect is independent of the glutathione depletion in HaCaT keratinocytes after solar light irradiation. J Cell Biochem.

[CR131] Jugert FK, Agarwal R, Kuhn A, Bickers DR, Merk HF, Mukhtar H (1994). Multiple cytochrome P450 isozymes in murine skin: induction of P450 1A, 2B, 2E, and 3A by dexamethasone. J Invest Dermatol.

[CR132] Kang-Sickel JC, Stober VP, French JE, Nylander-French LA (2010). Exposure to naphthalene induces naphthyl-keratin adducts in human epidermis in vitro and in vivo. Biomarkers.

[CR133] Kao J, Patterson FK, Hall J (1985). Skin penetration and metabolism of topically applied chemicals in 6 mammalian-species, including man—an in vitro study with benzo[a]pyrene and testosterone. Toxicol Appl Pharmacol.

[CR134] Katiyar SK, Matsui MS, Mukhtar H (2000). Ultraviolet-B exposure of human skin induces cytochromes P450 1A1 and 1B1. J Invest Dermatol.

[CR135] Katoh M, Hamajima F, Ogasawara T, Hata K (2009). Assessment of human epidermal model LabCyte EPI-MODEL for in vitro skin irritation testing according to European Centre for the Validation of Alternative Methods (ECVAM)-validated protocol. J Toxicol Sci.

[CR136] Kawakubo Y, Merk HF, Masaoudi TA, Sieben S, Blomeke B (2000). N-Acetylation of paraphenylenediamine in human skin and keratinocytes. J Pharmacol Exp Ther.

[CR137] Keeney DS, Skinner C, Wei S, Friedberg T, Waterman MR (1998). A keratinocyte-specific epoxygenase, CYP2B12, metabolizes arachidonic acid with unusual selectivity, producing a single major epoxyeicosatrienoic acid. J Biol Chem.

[CR138] Keeney DS, Skinner C, Travers JB, Capdevila JH, Nanney LB, King LE, Waterman MR (1998). Differentiating keratinocytes express a novel cytochrome P450 enzyme, CYP2B19, having arachidonate monooxygenase activity. J Biol Chem.

[CR139] Khan WA, Park SS, Gelboin HV, Bickers DR, Mukhtar H (1989). Epidermal cytochrome P-450: immunochemical characterization of isoform induced by topical application of3-methylcholanthrene to neonatal rat. J Pharmacol Exp Ther.

[CR140] Khan IU, Bickers DR, Haqqi TM, Mukhtar H (1992). Induction of CYP1A1 mRNA in rat epidermis and cultured human epidermal keratinocytes by benz(a)anthracene and betanaphthoflavone. Drug Metab Dispos.

[CR141] Kim PM, Wells PM (1996). Genoprotection by UDP-glucuronosyltransferases in peroxidasedependent, reactive oxygen species-mediated micronucleus initiation by the carcinogens-(methylnitrosamino)-1-(3-pyridyl)-1-butanone and benzo[a]pyrene. Cancer Res.

[CR142] Kim PM, Winn LM, Parman T, Wells PG (1997). UDP-glucuronosyltransferase-mediated protection against in vitro DNA oxidation and micronucleus formation initiated by phenytoin and its embryotoxic metabolite 5-(p-hydroxyphenyl)-5-phenylhydantoin. J Pharmacol Exp Ther.

[CR143] Klipper W, Weindl G, Schafer-Korting M (2010) Biotransformation of prednicarbate in reconstructed human skin models compared to excised human skin. EUSAAT 2010 10.1016/j.ejpb.2012.11.008 23201050

[CR144] Korać B, Buzadzić B (2001). Doxorubicin toxicity to the skin: possibility of protection with antioxidants enriched yeast. J Dermatol Sci.

[CR145] Labrie F, Luu-The V, Labrie C, Pelletier G, El-Alfy M (2000). Intracrinology and the skin. Horm Res.

[CR146] Laizure SC, Herring V, Hu Z, Witbrodt K, Parker RB (2013). The role of human carboxylesterases in drug metabolism: have we overlooked their importance?. Pharmacotherapy.

[CR147] Lamb KA, Denyer SP, Sanderson FD, Shaw PN (1994). The metabolism of a series of ester pro-drugs by NCTC 2544 cells, skin homogenate and LDE testskin. J Pharm Pharmacol.

[CR148] Ledirac N, Delescluse C, de Sousa G, Pralavorio M, Lesca P, Amichot M, Berge JB, Rahmani R (1997). Carbaryl induces CYP1A1 gene expression in HepG2 and HaCaT cells but is not a ligand of the human hepatic Ah receptor. Toxicol Appl Pharmacol.

[CR149] Lee AY, Lee KH, Ko DS, Chey WY (2001). Constitutive expression and changes of cytochrome P450 isozymes mRNAs by vehicles (petrolatum, DMSO, ethanol) in rat skin using semi-quantitative RT-PCR. Korean J Physiol Pharmacol.

[CR150] Lee JL, Mukhtar H, Bickers DR, Kopelovich L, Athar M (2003). Cyclooxygenases in the skin: pharmacological and toxicological implications. Toxicol Appl Pharmacol.

[CR151] Leong J, Hughes-Fulford M, Rakhlin N, Habib A, Maclouf J, Goldyne ME (1996). Cyclooxygenases in human and mouse skin and cultured human keratinocytes: association of COX-2 expression with human keratinocyte differentiation. Exp Cell Res.

[CR152] Levin W, Conney AH, Alvares AP, Merkatz I, Kappas A (1972). Induction of benzo()pyrene hydroxylase in human skin. Science.

[CR153] Levtchenko E, de Graaf-Hess A, Wilmer M, von den Heuvel L, Monnens L, Blom H (2005). Altered status of glutathione and its metabolites in cystinotic cells. Nephrol Dialysis Transplant.

[CR154] Li W, Tang Y, Hoshino T, Neya S (2009). Molecular modeling of human cytochrome P450 2W1 and its interactions with substrates. J Mol Graph Model.

[CR155] Liu H, Ling Y, Gong Y, Sun Y, Hou L, Zhang B (2007). DNA damage induces N-acetyltransferase NAT10 gene expression through transcriptional activation. Mol Cell Biochem.

[CR156] Lockley DJ, Howes D, Williams FM (2005). Cutaneous metabolism of glycol ethers. Arch Toxicol.

[CR157] Luu-The V, Duche D, Ferraris C, Meunier JR, Leclaire J, Labrie F (2009). Expression profiles of phases 1 and 2 metabolizing enzymes in human skin and the reconstructed skin models Episkin and full thickness model from Episkin. J Steroid Biochem Mol Biol.

[CR158] Mahns A, Wolber R, Stäb F, Klotz LO, Sies H (2004). Contribution of UVB and UVA to UV-dependent stimulation of cyclooxygenase-2 expression in artificial epidermis. Photochem Photobiol Sci.

[CR159] McCracken NW, Blain PG, Williams FM (1993). Nature and role of xenobiotic metabolizing esterases in rat liver, lung, skin and blood. Biochem Pharmacol.

[CR160] Merk HF (2009). Drug skin metabolites and allergic drug reactions. Curr Opin Allergy Clin Immunol.

[CR161] Merk HF, Baron JM (2004). Die Wirkung kleinmolekularer Substanzen auf die menschliche Haut. Molekulare Mechanismen und Konsequenzen. Hautarzt.

[CR162] Merk HF, Mukhtar H, Kaufmann I, Das M, Bickers DR (1987). Human hair follicle benzo[a]pyrene and benzo[a]pyrene 7,8-diol metabolism: effect of exposure to a coal tar-containing shampoo. J Invest Dermatol.

[CR163] Merk HF, Mukhtar H, Schutte B, Kaufmann I, Das M, Bickers DR (1987). 7-Ethoxyresorufin-O-deethylase activity in human hair roots: a potential marker for toxifying species of cytochrome P-450 isozymes. Biochem Biophys Res Commun.

[CR164] Merk HF, Khan WA, Kuhn C, Bickers DR, Mukhtar H (1989). Effect of topical application of clotrimazole to rats on epidermal and hepatic monooxygenase activities and cytochrome P-450. Arch Dermatol Res.

[CR165] Merk H, Jugert F, Bonnekoh B, Mahrle G (1991). Induction and inhibition of NAD(P)H: quinone reductase in murine and human skin. Skin Pharmacol.

[CR166] Mewes KR, Raus M, Bernd A, Zöller NN, Sättler A, Graf R (2007). Elastin expression in a newly developed full-thickness skin equivalent. Skin Pharmacol Physiol.

[CR167] Minchin RF, Hanna PE, Dupret JM, Wagner CR, Rodrigues-Lima F, Butcher NJ (2007). Int J Biochem Cell Biol.

[CR168] Moloney SJ, Fromson JM, Bridges JW (1982). Cytochrome P-450 dependent deethylase activity in rat and hairless mouse skin microsomes. Biochem Pharmacol.

[CR169] Moloney SJ, Bridges JW, Fromson JM (1982). UDP-glucuronosyltransferase activity in ratand hairless mouse skin-microsomes. Xenobiotica.

[CR170] Morris AP, Brain KR, Heard CM (2009). Skin permeation and ex vivo skin metabolism of O-acyl haloperidol ester prodrugs. Int J Pharm.

[CR171] Moss T, Howes D, Williams FM (2000). Percutaneous penetration and dermal metabolism of triclosan (2, 4, 4′-trichloro-2′-hydroxydiphenyl ether). Fd Chem Toxicol.

[CR172] Mukhtar H, Bickers DR (1981). Drug metabolism in skin. Comparative activity of the mixedfunction oxidases, epoxide hydratase, and glutathione S-transferase in liver and skin of the neonatal rat. Drug Metab Dispos.

[CR173] Mukhtar H, Bickers DR (1983). Age related changes in benzo(a)pyrene metabolism and epoxide metabolizing enzyme activities in rat skin. Drug Metab Dispos.

[CR174] Mukhtar H, Bresnick E (1976). Glutathione-S-epoxide transferase in mouse skin and human foreskin. J Invest Dermatol.

[CR175] Mukhtar H, Del Tito BJ, Jr Das M, Cherniack EP, Cherniack AD, Bickers DR (1984). Clotrimazole, an inhibitor of epidermal benzo(a)pyrene metabolism and DNA binding and carcinogenicity of the hydrocarbon. Cancer Res.

[CR176] Mukhtar H, Athar M, Bickers DR (1987). Cytochrome P-450 dependent metabolism of testosterone in rat skin. Biochem Biophys Res Commun.

[CR177] Mukhtar H, Bik DP, Ruzicka T, Merk HF, Bickers DR (1989). Cytochrome P-450-dependent omega-oxidation of leukotriene B4 in rodent and human epidermis. J Invest Dermatol.

[CR178] Murray GI, Taylor MC, McFadyen MC, McKay JA, Greenlee WF, Burke MD, Melvin WT (1997). Tumor-specific expression of cytochrome P450 CYP1B1. Cancer Res.

[CR179] Nabi Z, Tavakkol A, Dobke M, Polefka TG (2001). Bioconversion of vitamin E acetate in human skin. Curr Probl Dermatol.

[CR180] Nebert DW, Dalton TP, Okey AB, Gonzalez FJ (2004). Role of aryl hydrocarbon receptormediated induction of the CYP1 enzymes in environmental toxicity and cancer. J Biol Chem.

[CR181] Neis MM, Wendel A, Wiederholt T, Marquardt Y, Joussen S, Baron JM, Merk HF (2010). Expression and induction of cytochrome p450 isoenzymes in human skin equivalents. Skin Pharmacol Physiol.

[CR182] Netzlaff F, Lehr CM, Wertz PW, Schaefer UF (2005). The human epidermis models EpiSkin, SkinEthic and EpiDerm: an evaluation of morphology and their suitability for testing phototoxicity, irritancy, corrosivity, and substance transport. Eur J Pharm Biopharm.

[CR183] Nohynek GJ, Duche D, Garrigues A, Meunier PA, Toutain H, Leclaire J (2005). Under the skin: biotransformation of para-aminophenol and para-phenylenediamine in reconstructed human epidermis and human hepatocytes. Toxicol Lett.

[CR184] Oesch F, Arand M, Marquardt H, Schäfer S, McLellan D, Welsch C (1999). Xenobiotic metabolism. Toxicology.

[CR185] Oesch F, Bentley P (1976). Antibodies against homogeneous epoxide hydratase provide evidence for a single enzyme hydrating styrene oxide and benz(a)pyrene 4,5-oxide. Nature.

[CR187] Oesch F, Glatt HR, Schmassmann HU (1977). The apparent ubiquity of epoxide hydratase in rat organs. Biochem Pharmacol.

[CR188] Oesch F, Schmassmann H, Bentley P (1978). Specificity of human, rat and mouse skin epoxide hydratase towards K-region epoxides of polycyclic hydrocarbons. Biochem Pharmacol.

[CR189] Oesch F, Tegtmeyer F, Kohl FV, Rudiger H, Glatt HR (1980). Interindividual comparison of epoxide hydratase and glutathione S-transferase activities in cultured human fibroblasts. Carcinogenesis.

[CR190] Oesch F, Fabian E, Oesch-Bartlomowicz B, Werner C, Landsiedel R (2007). Drug-metabolizing enzymes in the skin of man, rat, and pig. Drug Metab Rev.

[CR191] Oesch-Bartlomowicz B, Oesch F, Testa B, van de Waterbeemd H (2007). Mechanisms of toxification and detoxification which challenge drug candidates and drugs. Comprehensive medicinal chemistry.

[CR192] Oh SY, Fujii M, Takeda Y, Yoda K, Utoguchi N, Matsumoto M, Watanabe Y (2002). The effect of ethanol on the simultaneous transport and metabolism of methyl p-hydroxybenzoate in excised skin of Yucatan micropig. Int J Pharm.

[CR193] Okun JG, Sauer S, Bahr S, Lenhartz H, Mayatepek E (2004). S-Acetylglutathione normalizes intracellular glutathione content in cultured fibroblasts from patients with glutathione synthetase deficiency. J Inherit Metab Dis.

[CR194] Ozawa N, Yamazaki S, Chiba K, Aoyama H, Tomisawa H, Tateishi M, Watabe T (1991). Biochem Biophys Res Commun.

[CR195] Pendlington RU, Williams DL, Naik JT, Sharma RK (1994). Distribution of xenobiotic metabolizing enzymes in skin. Toxicol In Vitro.

[CR196] Peters WH, Allebes WA, Jansen PL, Poels LG, Cape PJ (1987). Characterization and tissue specificity of a monoclonal antibody against human uridine 5′-diphosphateglucuronosyltransferase. Gastroenterology.

[CR197] Pham MA, Magdalou J, Totis M, Fournel-Gigleux S, Siest G, Hammock BD (1989). Characterization of distinct forms of cytochromes P-450, epoxide metabolizing enzymes and UDP-glucuronosyltransferases in rat skin. Biochem Pharmacol.

[CR198] Pham MA, Magdalou J, Siest G, Lenoir MC, Bernard BA, Jamoulle JC, Shroot B (1990). Reconstituted epidermis: a novel model for the study of drug metabolism in human epidermis. J Invest Dermatol.

[CR199] Phillips TL, Mitchell JB, de Graff W, Russo A, Glatstein E (1986). Variation in sensitizing efficiency for SR 2508 in human cells dependent on glutathione content. Int J Radiat Oncol Biol Phys.

[CR200] Pillai O, Hamad MO, Crooks PA, Stinchcomb AL (2004). Physicochemical evaluation, in vitro human skin diffusion, and concurrent biotransformation of 3-O-alkyl carbonate prodrugs of naltrexone. Pharm Res.

[CR201] Pohl RJ, Philpot RM, Fouts JR (1976). Cytochrome P-450 content and mixed-function oxidase activity in microsomes isolated from mouse skin. Drug Metab Dispos.

[CR202] Prusakiewicz JJ, Ackermann C, Voorman R (2006). Comparison of skin esterase activities from different species. Pharm Res.

[CR502] Qin Z (2012) The use of THP-1 cells as a model for mimicking the function and regulation of monocytes and macrophages in the vasculature. Atherosclerosis 221:2–114 10.1016/j.atherosclerosis.2011.09.003 21978918

[CR203] Raffali F, Rougier A, Roguet R (1994). Measurement and modulation of cytochrome-P450-dependent enzyme activity in cultured human keratinocytes. Skin Pharmacol.

[CR284] Ramirez T, Mehling A, Kolle SN, Wruck CJ, Teubner W, Eltze T, Aumann A, Urbisch D, van Ravenzwaay B, Landsiedel R (2014) LuSens: A keratinocyte based ARE reporter gene assay for use in integrated testing strategies for skin sensitization hazard identification. Toxicol In Vitro 28:1482–1497 10.1016/j.tiv.2014.08.002 25172300

[CR204] Rangarajan M, Zatz JL (2001). Effect of formulation on the delivery and metabolism of alphatocopheryl acetate. J Cosmet Sci.

[CR205] Rassmussen C, Gratz N, Simon N, Van der Zanden C, Johnston C, Allen-Hoffmann BL (2011). Expression and induction of xenobiotic metabolism genes in the Strata Test human skin model. SOT 2011. Toxicologist.

[CR206] Raza H, Awasthi YC, Zaim MT, Eckert RL, Mukhtar H (1991). Glutathione S-transferasesin human and rodent skin: multiple forms and species specific expression. J Invest Dermatol.

[CR207] Raza H, Agarwal R, Bickers DR, Mukhtar H (1992). Purification and molecular characterization of beta-naphthoflavone-inducible cytochrome P-450 from rat epidermis. J Invest Dermatol.

[CR208] Rea MA, Gregg JP, Rice RH (2002). Expression profiling of TCDD-treated human epidermal cells in culture. Organohalogen Compd.

[CR209] Rees RS, Kingman GJ, Cashmer B, Gilmont RR, Reeves C, Welsh MJ, Smith DJ (1994). dT diaphorase: increased enzyme activity and mRNA expression in oxidant stress of skin. J Surg Res.

[CR210] Reilly TP, Lash LH, Doll MA, Hein DW, Woster PM, Svensson CK (2000). A role for bioactivation and covalent binding within epidermal keratinocytes in sulfonamide-induced cutaneous drug reactions. J Invest Dermatol.

[CR211] Reiners JJ, Cantu AR, Thai G, Schöller A (1992). Differential expression of basal and hydrocarbon-induced cytochrome P-450 monooxygenase and quinone reductase activities in subpopulations of murine epidermal cells differing in their stages of differentiation. Drug Metab Dispos.

[CR212] Richter-Hintz D, Their R, Steinwachs S, Kronenberg S, Fritsche E, Sachs B, Wulferink M, Tonn T, Esser C (2003). Allelic variants of drug metabolizing enzymes as risk factors in psoriasis. J Invest Dermatol.

[CR213] Rittirod T, Hatanaka T, Uraki A, Hino K, Katayama K, Koizumi T (1999). Species difference in simultaneous transport and metabolism of ethyl nicotinate in skin. Int J Pharm.

[CR214] Rolsted K, Kissmeyer AM, Rist GM, Hansen SH (2008). Evaluation of cytochrome P450 activity in vitro, using dermal and hepatic microsomes from four species and two keratinocyte cell lines in culture. Arch Dermatol Res.

[CR215] Romeu M, Mulero M, Giralt M, Folch J, Nogues MR, Torres A, Fortuno A, Sureda FX, Cabre M, Paternain JL, Mallo J (2002). Parameters related to oxygen free radicals in erythrocytes, plasma and epidermis of the hairless rat. Life Sci.

[CR216] Roychowdhury S, Svensson CK (2005). Mechanisms of drug-induced delayed-type hypersensitivity reactions in the skin. AAPS J.

[CR217] Ruzicka T, Printz MP (1982). Arachidonic acid metabolism in guinea pig skin. Biochim Biophys Acta.

[CR218] Saarikoski ST, Wikman HA, Smith G, Wolff CH, Husgafvel-Pursiainen K (2005). Localization of cytochrome P450 CYP2S1 expression in human tissues by in situ hybridisation and immunohistochemistry. J Histochem Cytochem.

[CR219] Saarikoski ST, Rivera SP, Hankinson O, Husgafvel-Pursiainen K (2005). CYP2S1: a short review. Toxicol Appl Pharmacol.

[CR220] Saeki M, Saito Y, Nagano M, Teshima R, Ozawa S, Sawada J (2002). mRNA expression of multiple cytochrome p450 isozymes in four types of cultured skin cells. Int Arch Allergy Immunol.

[CR221] Saleem MM, Al-Tamer YY, Skursky L, Al-Habbal Z (1984). Alcohol dehydrogenase activity in the human tissues. Biochem Med.

[CR222] Samuelsson K, Bergström MA, Jonsson CA, Westman G, Karlberg AT (2011). Diphenylthiourea, a common rubber chemical, is bioactivated to potent skin sensitizers. Chem Res Toxicol.

[CR223] Särnstrand B, Eriksson G, Malmström A (1987). Glucocorticoids change the nucleotide and sugar nucleotide pool sizes in cultured human skin fibroblasts. Arch Biochem Biophys.

[CR224] Schober W, Luch A, Soballa VJ, Raab G, Stegeman JJ, Doehmer J, Jacob J, Seidel A (2006). On the species-specific biotransformation of dibenzo[a, l]pyrene. Chem Biol Interact.

[CR225] Semak I, Korik E, Naumova M, Wortsman J, Slominski A (2004). Serotonin metabolism in rat skin: characterization by liquid chromatography-massspectrometry. Arch Biochem Biophys.

[CR226] Shuster S, Rawlins MD, Chapman PH, Rogers S (1980). Decreased epidermal aryl hydrocarbon hydroxylase and localized pustular psoriasis. Br J Dermatol.

[CR227] Sieben S, Baron JM, Blomeke B, Merk HF (1999). Multiple cytochrome P450-isoenzymes mRNA are expressed in dendritic cells. Int Arch Allergy Immunol.

[CR228] Sivarajah K, Lasker JM, Eling TE (1981). Prostaglandin synthetase-dependent cooxidation of (±)-benzo(a)pyrene-7,8-dihydrodiol by human lung and other mammalian tissues. Cancer Res.

[CR229] Slavik MA, Allen-Hoffmann BL, Liu BY, Alexander CM (2007). Wnt signaling induces differentiation of progenitor cells in organotypic keratinocyte cultures. BMC Dev Biol.

[CR230] Smith CK, Moore CA, Elahi EN, Smart AT, Hotchkiss SA (2000). Human skin absorption and metabolism of the contact allergens, cinnamic aldehyde, and cinnamic alcohol. Toxicol Appl Pharmacol.

[CR231] Smith G, Dawe RS, Clark C, Evans AT, Comrie MM, Wolf CR, Ferguson J, Ibbotson SH (2003). Quantitative real-time reverse transcription-polymerase chain reaction analysis of drug metabolizing and cytoprotective genes in psoriasis and regulation by ultraviolet radiation. J Invest Dermatol.

[CR232] Smith G, Wolf CR, Deeni YY, Dawe RS, Evans AT, Comrie MM, Ferguson J, Ibbotson SH (2003). Cutaneous expression of cytochrome P450 CYP2S1: individuality in regulation by therapeutic agents for psoriasis and other skin diseases. Lancet.

[CR233] Smith G, Ibbotson SH, Comrie MM, Dawe RS, Bryden A, Ferguson J, Wolf CR (2006). Regulation of cutaneous drug-metabolizing enzymes and cytoprotective gene expression by topical drugs in human skin in vivo. Br J Dermatol.

[CR234] Storm JE, Collier SW, Stewart RF, Bronaugh RL (1990). Metabolism of Xenobiotics during Percutaneous Penetration: role of Absorption Rate and Cutaneous Enzyme Activity. Fund Appl Toxicol.

[CR235] Strohm BH, Kulkarni AP (1986). Peroxidase, an alternate pathway to cytochrome P-450 for xenobiotic metabolism in skin: partial purification and properties of the enzyme from neonatal rat skin. J Biochem Toxicol.

[CR236] Sugibayashi K, Hayashi T, Morimoto Y (1999). Simultaneous transport and metabolism of ethyl nicotinate in hairless rat skin after its topical application: the effect of enzyme distribution in skin. J Control Rel.

[CR237] Sugibayashi K, Hayashi T, Matsumoto K, Hasegawa T (2004). Utility of a three-dimensional cultured human skin model as a tool to evaluate the simultaneous diffusion and metabolism of ethyl nicotinate in skin. Drug Metab Pharmacokin.

[CR238] Suppasansatorn P, Wang G, Conway BR, Wang W, Wang Y (2006). Skin delivery potency and antitumor activities of temozolomide ester prodrugs. Cancer Lett.

[CR239] Sutter TR, Tang YM, Hayes CL, Wo YY, Jabs EW, Li X, Yin H, Cody CW, Greenlee WF (1994). Complete cDNA sequence of a human dioxin-inducible mRNA identifies a new gene subfamily of cytochrome P450 that maps to chromosome 2. J Biol Chem.

[CR240] Swanson HI (2004). Cytochrome P450 expression in human keratinocytes: an aryl hydrocarbon receptor perspective. Chem Biol Interact.

[CR241] Szotáková B, Baliharová V, Lamka J, Nozinová E, Wsól V, Velík J, Machala M, Neca J, Soucek P, Susová S, Skálová L (2004). Comparison of in vitro activities of biotransformation enzymes in pig, cattle, goat and sheep. Res Vet Sci.

[CR242] Takahara H, Zaidi SI, Mukhtar H, Handa M, Epstein WL, Fukuyama K (1993). Purification and characterization of NADPH-cytochrome P-450 reductase from rat epidermis. J Cell Biochem.

[CR243] Thiele B, Merk HF, Bonnekoh B, Mahrle G, Steigleder GK (1987). Epidermal cell growth-dependent arylhydrocarbon-hydroxylase (AHH) activity in vitro. Arch Dermatol Res.

[CR244] Tinois E, Tiollier J, Gaucherand M, Dumas H, Tardy M, Thivolet J (1991). In vitro and post-transplantation differentiation of human keratinocytes grown on the human type IV collagen film of a bilayered dermal substitute. Exp Cell Res.

[CR245] Törmä H, Vahlquist A (1990). Vitamin A esterification in human epidermis: a relation to keratinocyte differentiation. J Invest Dermatol.

[CR246] Tse FL, Laplanche R (1998). Absorption, metabolism, and disposition of [14C]SDZ ENA 713, an acetylcholinesterase inhibitor, in minipigs following oral, intravenous, and dermal administration. Pharm Res.

[CR247] Tukey RH, Strassburg CP (2000). Human UDP-glucuronosyltransferases: metabolism, expression, and disease. Annu Rev Pharmacol Toxicol.

[CR248] Tunali T, Sener G, Yarat A, Emekli N (2004). Melatonin reduces oxidative damage to skin and normalizes blood coagulation in a rat model of thermal injury. Life Sci.

[CR249] Ueda O, Kitamura S, Ohashi K, Sugihara K, Ohta S (2003). Xanthine oxidase-catalyzed metabolism of 2-nitrofluorene, a carcinogenic air pollutant, in rat skin. Drug Metab Dispos.

[CR250] van de Sandt JJ, Rutten AA, van Ommen B (1993). Species-specific cutaneous biotransformation of the pesticide propoxur during percutaneous absorption in vitro. Toxicol Appl Pharmacol.

[CR251] van Eijl S, Zhu Z, Cupitt J, Gierula M, Götz C, Fritsche E, Edwards RJ (2012). Elucidation of xenobiotic metabolism pathways in human skin and human skin models by proteomic profiling. PLoS ONE.

[CR252] Vance WA, Wang YY, Okamoto HS (1987). Disubstituted amino-, nitroso- and nitrofluorenes: a physicochemical basis for structure-activity relationships in *Salmonella typhimurium*. Environ Mutagen.

[CR253] Vanden Bossche H, Willemsens G, Janssen PA (1988). Cytochrome-P-450-dependent metabolism of retinoic acid in rat skin microsomes: inhibition by ketoconazole. Skin Pharmacol.

[CR254] Vecchini F, Mace K, Magdalou J, Mahe Y, Bernard BA, Shroot B (1995). Constitutive and inducible expression of drug metabolizing enzymes in cultured human keratinocytes. Br J Dermatol.

[CR255] Vickers AEM, Biggi WA, Dannecker R, Fischer V (1995). Uptake and metabolism of cyclosporin A and SDZ IMM 125 in the human in vitro Skin2 dermal and barrier function models. Life Sci.

[CR256] Vienneau DS, DeBoni U, Wells PG (1995). Potential genoprotective role for UDP-glucuronosyltransferases in chemical carcinogenesis: initiation of micronuclei by benzo[a]pyrene and benzo[e]pyrene in UDP-glucuronosyltransferase–deficient cultured rat skin fibroblasts. Cancer Res.

[CR257] Villard PH, Sampol E, Elkaim JL, Puyoou F, Casanova D, Seree E, Durand A, Lacarelle B (2002). Increase of CYP1B1 transcription in human keratinocytes and HaCaT cells after UV-B exposure. Toxicol Appl Pharmacol.

[CR258] Vondracek M, Xi Z, Larsson P, Baker V, Mace K, Pfeifer A, Tjalve H, Donato MT, Gomez-Lechon MJ, Grafstrom RC (2001). Cytochrome P450 expression and related metabolism in human buccal mucosa. Carcinogenesis.

[CR259] Vondracek M, Weaver DA, Sarang Z, Hedberg JJ, Willey JC, Wärngård L, Grafström RC (2002). Transcript profiling of enzymes involved in detoxification of xenobiotics and reactive oxygen in human normal and simian virus 40 T antigen-immortalized oral keratinocytes. Int J Cancer.

[CR260] Vorrink SU, Severson PL, Kulak MV, Futscher BW, Domann FE (2014). Hypoxia perturbs aryl hydrocarbon receptor signaling and CYP1A1 expression induced by PCB 126 in human skin and liver-derived cell lines. Toxicol Appl Pharmacol.

[CR261] Vyas PM, Roychowdhury S, Koukouritak SB, Hines RN, Krueger SK, Williams DE, Nauseef WM, Svensson CK (2006). Enzyme-mediated protein haptenation of dapsoneand sulfamethoxazole in human keratinocytes: II. Expression and role of flavin-containing monooxygenases and peroxidases. J Pharmacol Exp Ther.

[CR262] Walraven JM, Doll MA, Hein DW (2006). Identification and characterization of functional rat arylamine N-acetyltransferase 3: comparisons with rat arylamine N-acetyltransferases 1 and 2. J Pharmacol Exp Ther.

[CR263] Wang K, Guengerich FP (2013). Reduction of aromatic and heterocyclic aromatic N-hydroxylamines by human cytochrome P450 2S1. Chem Res Toxicol.

[CR264] Wang L, Raghavan N, He K, Luettgen JM, Humphreys WG, Knabb RM, Pinto DJ, Zhang D (2009). Sulfation of o-demethyl apixaban: enzyme identification and species comparison. Drug Metab Dispos.

[CR265] Warwick E, Cassidy A, Hanley B, Jouni ZE, Bao Y (2012). Effect of phytochemicals on phase II enzyme expression in infant human primary skin fibroblast cells. Br J Nutr.

[CR266] Wattenberg LW, Leong JL (1962). Histochemical studies of polycyclic hydrocarbon metabolizing systems. J Histochem Soc..

[CR267] Westerlund M, Galter D, Carmine A, Olson L (2005). Tissue- and species-specific expression patterns of class I, III, and IV Adh and Aldh 1 mRNAs in rodent embryos. Cell Tissue Res.

[CR268] Whitlock JP (1987). The regulation of cytochrome P450 gene expression. Annu Rev Pharmacol Toxicol.

[CR269] Wiegand C, Reisinger K, Schroder K, Merk HF, Eschrich D (2008). Metabolism studies of the PHENION (R) full thickness skin model compared to other in vitro models. Naunyn-Schmied Arch Pharmacol.

[CR270] Wilkin LM, Stewart RF (1987). Substrate specificity of human cutaneous alcohol deyhdrogenase and erythema provoked by lower aliphatic alcohols. J Invest Dermatol.

[CR271] Williams D, Woodhouse K (1995). Age related changes in NADPH cytochrome c reductase activity in mouse skin and liver microsomes. Arch Gerontol Geriatr.

[CR272] Williams D, Woodhouse K (1996). Age-related changes in O-deethylase and aldrin epoxidase activity in mouse skin and liver microsomes. Age Ageing.

[CR273] Wong KO, Tan AY, Lim BG, Wong KP (1993). Sulphate conjugation of minoxidil in rat skin. Biochem Pharmacol.

[CR274] Wu ZL, Sohl CD, Shimada T, Guengerich FP (2006). Recombinant enzymes overexpressed in bacteria show broad catalytic specificity of human cytochrome P450 2W1 and limited activity of human cytochrome P450 2S1. Mol Pharmacol.

[CR275] Yarat A, Tunali T, Yanardag R, Gürsoy F, Sacan Ö, Emekli N, Üstüner A, Ergenekon G (2001). The effect of Glurenorm (gliquidone) on lenses and skin in experimental diabetes. Free Radic Biol Med.

[CR276] Yengi LG, Xiang Q, Pan J, Scatina J, Kao J, Ball SE, Fruncillo R, Ferron G, Wolf RC (2003). Quantitation of cytochrome P450 mRNA levels in human skin. Anal Biochem.

[CR277] Young HM, O’Brien AJ, Furness JB, Ciampoli D, Hardwick JP, McCabe TJ, Narayanasami R, Masters BS, Tracey WR (1997). Relationships between NADPH diaphorase staining and neuronal, endothelial, and inducible nitric oxide synthase and cytochrome P450 reductase immunoreactivities in guinea-pig tissues. Histochem Cell Biol.

[CR278] Yourick JJ, Bronaugh RL (1997). Percutaneous absorption and metabolism of Coumarin in human and rat skin. J Appl Toxicol.

[CR279] Yourick JJ, Bronaugh RL (2000). Percutaneous penetration and metabolism of 2-nitro-p-phenylenediamine in human and fuzzy rat skin. Toxicol Appl Pharmacol.

[CR280] Zalko D, Jacques C, Duplan H, Bruel S, Perdu E (2011). Viable skin efficiently absorbs and metabolizes bisphenol A. Chemosphere.

[CR281] Zhu Z, Hotchkiss SA, Boobis AR, Edwards RJ (2002). Expression of P450 enzymes in rat whole skin and cultured epidermal keratinocytes. Biochem Biophys Res Commun.

[CR282] Zhu QG, Hu JH, Liu JY, Lu SW, Liu YX, Wang J (2007). Stereoselective characteristics and mechanisms of epidermal carboxylesterase metabolism observed in HaCaT keratinocytes. Biol Pharm Bull.

